# Evolutionary and molecular basis of ADP-ribosylation reversal by zinc-dependent macrodomains

**DOI:** 10.1016/j.jbc.2024.107770

**Published:** 2024-09-11

**Authors:** Antonio Ariza, Qiang Liu, Nathan P. Cowieson, Ivan Ahel, Dmitri V. Filippov, Johannes Gregor Matthias Rack

**Affiliations:** 1 School of Biosciences, University of Sheffield, Sheffield, UK; 2 Sir William Dunn School of Pathology, University of Oxford, Oxford, UK; 3 Bio-Organic Synthesis, Leiden Institute of Chemistry, Leiden University, Leiden, The Netherlands; 4 Zhongshan Institute for Drug Discovery, Shanghai Institute of Materia Medica, Chinese Academy of Sciences, Beijing, China; 5 Chinese Academy of Sciences, Shanghai Institute of Materia Medica, Beijing, China; 6 Harwell Science and Innovation Campus, Diamond Light Source, Didcot, Oxfordshire, UK; 7 Medical Research Council Centre for Medical Mycology at the University of Exeter, University of Exeter, Exeter, UK

**Keywords:** lipoylation, Post-translational modification (PTM), Metalloenzyme, X-ray crystallography, small-angle X-ray scattering (SAXS)

## Abstract

Dynamic ADP-ribosylation signaling is a crucial pathway that controls fundamental cellular processes, in particular, the response to cellular stresses such as DNA damage, reactive oxygen species, and infection. In some pathogenic microbes, the response to oxidative stress is controlled by a SirTM/zinc-containing macrodomain (Zn-Macro) pair responsible for establishment and removal of the modification, respectively. Targeting this defence mechanism against the host’s innate immune response may lead to novel approaches to support the fight against emerging antimicrobial resistance. Earlier studies suggested that Zn-Macros play a key role in the activation of this defence. Therefore, we used phylogenetic, biochemical, and structural approaches to elucidate the functional properties of these enzymes. Using the substrate mimetic asparagine-ADP-ribose as well as the ADP-ribose product, we characterize the catalytic role of the zinc ion in the removal of the ADP-ribosyl modification. Furthermore, we determined structural properties that contribute to substrate selectivity within the different Zn-Macro branches. Together, our data not only give new insights into the Zn-Macro family but also highlight their distinct features that may be exploited for the development of future therapies.

Bacterial and fungal infections pose a significant risk to human health (1, 2, 3). The problem is amplified by the development of antimicrobial resistance (AMR) and emergence of multidrug resistant strains associated with the loss of treatment options. To address this issue, the World Health Organization has published lists of priority pathogens that pose the greatest risk (4, 5). Overcoming AMR will require a multipronged approach, including the identification and characterization of new or neglected antimicrobial targets as well as evaluation of their therapeutic potential for novel (co-)treatment strategies.

We previously described an operon-encoded system present in major human pathogens, including *Staphylococcus aureus* and *Streptococcus pyogenes*, that relies on the crosstalk between two pathways of high therapeutic interest: lipoic acid metabolism and ADP-ribosylation signaling (Fig. 1). Lipoic acid is a small organosulfur cofactor that, when covalently attached to multicomponent dehydrogenases, participates in the intermediate metabolism of free-living cells (6). In addition, lipoylated proteins play a role in other crucial cellular processes including bacterial sporulation, gene expression, and oxidative defence (6, 7, 8, 9, 10). It is therefore not surprising that disruption of bacterial lipoic acid acquisition has been shown to reduce virulence in several models of infection (11, 12, 13). Similarly, ADP-ribosylation signaling plays a major role in processes such as the regulation of the host immune response or microbial immune evasion and host adaptation (14, 15, 16). ADP-ribosylation is a reversible, posttranslational modification involving the transfer of an ADP-ribose (ADPr) moiety from β-NAD^+^ onto a target residue within a protein (17, 18, 19). ADP-ribosylation “writers” and “erasers” can be identified in all kingdoms of life as well as several viruses. Although the signaling mechanism is better understood in higher eukaryotes (17, 19, 20, 21), where it affects genome stability, host immune response, transcription regulation, among others (16, 22, 23), extensive evidence is emerging for its crucial role in microorganisms including microbial immune evasion and host-adaptation, growth regulation, antiphage response, and intermicrobial warfare among others (14, 24, 25, 26, 27, 28, 29). The modification can either involve the transfer of a single ADPr unit (mono-ADP-ribosylation, MARylation) or the extension of the initial modification into linear or branched ADPr homopolymers (poly-ADP-ribosylation, PARylation). The enzymes responsible for establishing the modification, termed (ADP-ribosyl)transferases, belong usually to the PARP or ARTC family (17, 18, 30). However, certain sirtuins, which are more commonly associated with NAD^+^-dependent deacylation, have been reported to harbor transferase activity, too (19, 25, 31, 32).

**Figure 1:**
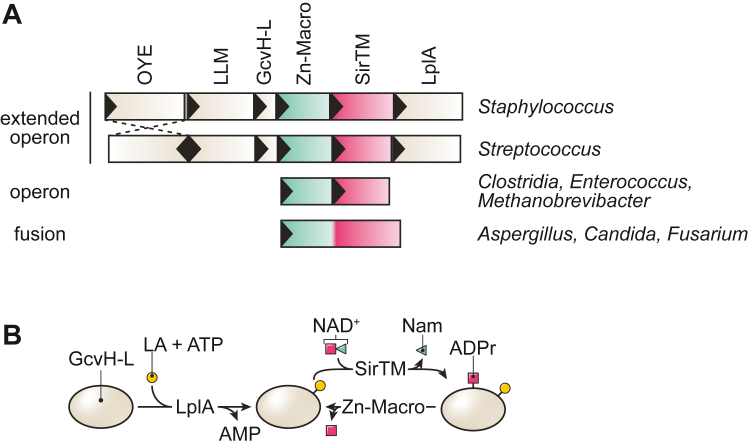
**Genomic organization and functional role of Zn-Macros.***A*, genomic architecture of the Zn-Macro (*green*)/SirTM (*red*) pair. The GcvH-L, Zn-Macro, SirTM, and LplA region contains only coding nucleotides with some genes partially overlapping, whereas the OYE/LMM pair is slightly removed and OYE is, in some species, encoded on the opposing strand relative to the rest of the operon (indicated by *dashed lines*). *B*, functional schematic showing lipoylation by LplA in a lipoic acid (LA)- and ATP-dependent manner. This is a prerequisite for the ADP-ribosylation by SirTM. The latter reaction is reversible by Zn-Macros. The functional relevance for LLM and OYE remains so far elusive but may be linked to the reversible oxidation of the lipoyl moiety (see discussion).

Recently, microbial and viral macrodomains, which can reverse ADP-ribosylation signaling, have come into focus as potential therapeutic targets (15, 33, 34, 35). Macrodomains are ancient, evolutionary conserved structural modules that can be found in all kingdoms of life as well as some viruses (36, 37). Structurally, they exhibit an α/β/α sandwich fold of typically 130 to 190 amino acids with a deep ligand-binding cleft on the crest of the domain (38, 39, 40, 41). Macrodomains can bind ADP-ribosylated ligands *via* this cleft and act either as signal “readers” or “erasers.” Phylogenetically, the macrodomain superfamily can be subdivided into at least six evolutionary distinct families with differences in their mode of ADPr recognition and hydrolysis (36, 37). Among these, the MacroD-type family contains several members of pharmacological interest, including viral macrodomains important for alphavirus and coronavirus replication as well as the human immune response (16, 33, 34, 42, 43, 44). Furthermore, it was observed that these viral as well as homologous, PARP-associated macrodomains are under ongoing positive selection, which further supports their importance in the host-pathogen arms race (45, 46). As aforementioned, the operon-encoded system we described earlier contains an unusual, genetically linked macrodomain-sirtuin pair and plays a role in the oxidative stress response of bacterial and fungal pathogens (31). This pair can be identified either alone, in the context of an extended operon, or fused into a single polypeptide chain (Fig. 1*A*). In the context of the extended operon, ADP-ribosylation occurs in a sequential order: the encoded carrier protein, glycine cleavage system H-like (GcvH-L), is first lipoylated by a lipoyl protein ligase (LplA) and can subsequently be ADP-ribosylated by the macrodomain-linked sirtuin (SirTM) (Fig. 1*B*). The MARylation can be reversed by a zinc-containing macrodomain (Zn-Macro). SirTMs appear to be the only sirtuins that have only (ADP-ribosyl)transferase activity due to an exchange of a key histidine residue with glutamine, while a glycine-rich stretch in the catalytic loop of the macrodomain was replaced by a zinc-binding motif (31, 47). While it was suggested that the zinc ion contributes to the catalytic activity of the macrodomain and is responsible for the observed ability to cleave the *S*-glycolytic bond in ADP-ribosylated cysteine residues (47, 48), experimental evidence is so far lacking. Moreover, GcvH-L is at present the only known protein ADP-ribosylated by a SirTM: whether other targets outside the extended operon exist, what the nature of targets in other SirTM/Zn-Macro systems is, and what the physiological role of SirTM/Zn-Macro signalling is, remains elusive.

In this study, we describe the evolutionary, biochemical, and structural basis of the (ADP-ribosyl)hydrolase activity of Zn-Macros. We highlight their unique enzymatic properties and demonstrate that their function is strictly dependent on a catalytic zinc within the active site. Furthermore, we identified structural features that are important for lipoyl recognition as well as GcvH-L demodification. Members of the Zn-Macro subfamily play an important role in the defence against oxidative stress response, a potent host defence mechanism.

## Results

### Zinc-containing macrodomains are of the MacroD-type

**Figure 2:**
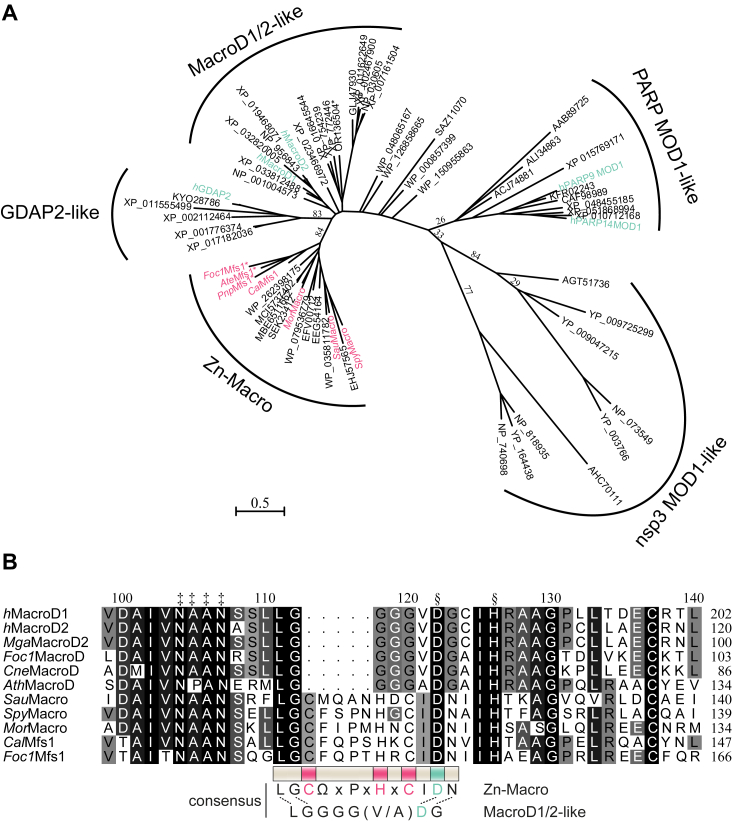
**Phylogenetic analysis of the MacroD-type family.***A*, evolutionary phylogenetic tree analysis of MacroD-type domain: the tree was constructed with amino acid sequences isolated from their whole protein context by multiple sequence alignment. The evolutionary history was inferred by using the maximum likelihood method and LG model of amino acid substitution as implemented in MEGA 11. The tree with the highest log likelihood (−14087.41) is shown. The tree is drawn to scale, with branch lengths measured in the number of substitutions per site. Subclasses are indicated and named according to prominent members. Zn-Macros used in this study are indicated in *red*, human proteins in *green*, and asterisks mark sequences identified in this study. *B*, multiple sequence alignment of the ‘catalytic/Zn-loop’ region of representative MacroD1/2-like and Zn-Macros. Consensus sequences [ELM nomenclature (108)] for ‘classic’ and zinc coordination loop are given below the alignment with Zn^2+^-coordinating residues highlighted in *red*. Isostructural residues are indicated by dashed lines and the NAAN (‡) as well as catalytic (§) motifs above the alignment. A full alignment with all MacroD-type subclasses is shown in Fig. S1 and sequence information are given in Tables. S1 and S2.

We showed previously that zinc-containing macrodomains (Zn-Macros) play a role in the oxidative stress response of bacterial and fungal pathogens (31). These macrodomains have structural features that place them within the MacroD-type family, but closer phylogenetic assessment was still outstanding (31, 47). Therefore, we analyzed the relationship of Zn-Macros to known members of the MacroD-type class. Focusing on sequence position with more than 95% site coverage, we found that Zn-Macros cluster with GDAP2- and MacroD1/2-like macrodomains, whereas they are less closely related to viral and PARP9/14 macrodomain 1–like macrodomains (Fig. 2*A* and Table S1). As yet, no catalytic activity has been reported for GDAP2-like macrodomains (36, 49) and they have lost all previously described features, such as potential catalytic residues, the active site arene, and NAAN motif (Fig, S1 and Table S2), associated with catalytic activity (36, 37). Zn-Macros contain the active site arene, NAAN motif, as well as Asp/His catalytic dyad found in MacroD1/2like macrodomains. Strikingly, however, the “catalytic loop” found in all other MacroD-type enzymes (also termed “loop 1”) is replaced with and extended loop containing the zinc-coordinating Cx_(__4__)_HxC motif, here termed the Zn-loop (Figs. 2*B* and S1). In addition, all members of the subfamily carry an N-terminal extension, which structurally resolved into a three-alpha helical bundle (3α-bundle) (37, 47).

### Ligand coordination by Zn-Macros

**Figure 3:**
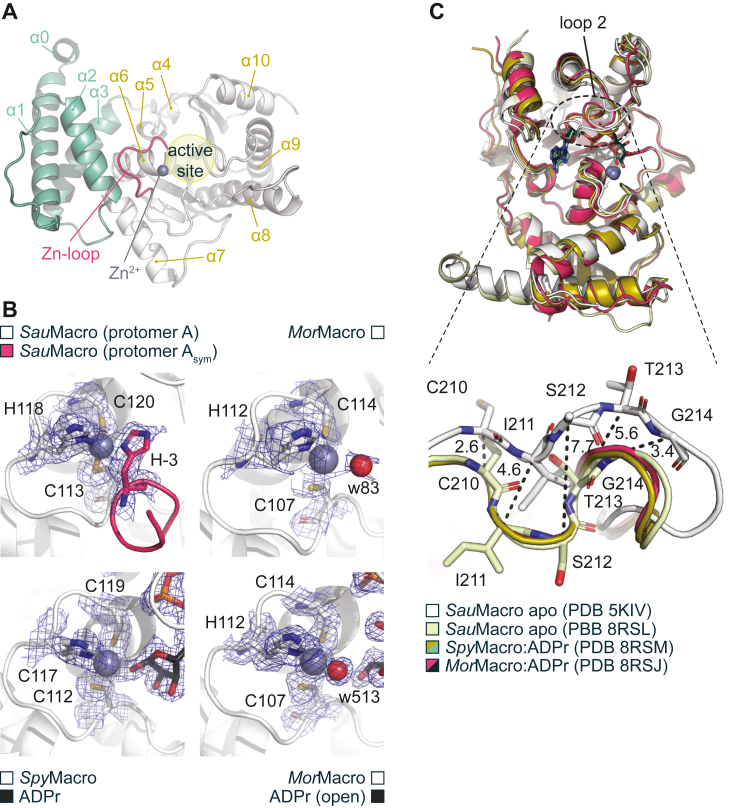
**Zn-Macros from the extended operon contain no lipoyl-binding pocket.***A*, ribbon representation of *Sau*Macro with the 3α-bundle in *green* and the macrodomain in *white*. The Zn-loop is highlighted in *red* and helices numbered. Note, helix α0 is part of the nonphysiological expression tag. *B*, electron density omit maps (2Fo-Fc contoured at 1 σ) refined in the absence of any ligand. The final refined protein–ligand structures are shown as reference. *C*, overlay of the previously solved apo *Sau*Macro structure (*white*) with the here reported structures of *Sau*Macro (*pale yellow*), *Spy*Macro:ADPr (*yellow*), and *Mor*Macro:ADPr (*red*). Conformational differences in the loop 2 region are indicated by the *dotted circle* (*upper* panel). *Lower* panel: Comparison of loop 2 conformation across Zn-Macro structures. Residues of the two *Sau*Macro structures are highlighted and C^α^ distances (in Ångström) between the here reported (PDB 8RSL) and previously solved (PDB 5KIV) apo *Sau*Macro shown as *dotted* lines.

To gain a closer understanding of the functional role of the 3α-bundle and coordinated Zn^2+^ ion, we solved structures from the three sub-branches of Zn-Macros namely from *S. aureus* (*Sau*Macro), *Methanobrevibacter oralis* (*Mor*Macro), and macrodomain-fused SirTM protein (Mfs1) from *Fusarium oxysporum* f.sp. *cubense* race 1 (*Foc1*Mfs1) in their apo form as well as of *S. pyogenes* (*Spy*Macro) and *Mor*Macro bound to ADP-ribose (ADPr) (Figs. 3*A*, S2 and Table 1). The overall fold of our apo structures resembles closely an earlier reported *Sau*Macro structure (PDB 5KIV) with an RMSD of 0.382 Å over 196 C^α^ (*Sau*Macro), 0.754 Å over 201 C^α^ (*Mor*Macro), and 1.125 Å over 153 C^α^ (*Foc1*Mfs1), respectively. Ligand binding marginally increased the RMSD value to 0.884 Å over 183 C^α^ (*Spy*Macro:ADPr) and 1.029 Å over 211 C^α^ (*Mor*Macro:ADPr) (Fig. S2, *A* and *B*).

**Table 1:** Crystallographic data collection, phasing, and refinement statistics

PDB accession code	*Sau*Macro (apo)	*Spy*Macro ADPr	*Mor*Macro (apo)	*Mor*Macro ADPr	*Mor*Macro Asn-ADPr	*Foc1*Mfs1 (apo)
	8RSL	8RSM	8RSI	8RSJ	8RSK	8RSN
Data collection						
Synchrotron/beamline	DLS/I04-1	DLS/I03	DLS/I03	ESRF/MASSIF-1	DLS/I04-1	DLS/I03
Detector	PILATUS 2M	PILATUS3 6M	PILATUS3 6M	PILATUS3 6M	PILATUS3 6M	PILATUS3 6M
Wavelength (Å)	0.92000	01.28220	1.28229	0.96598	0.92819	1.28229
Space group	*P*2_1_	*P*4_1_	*P*2_1_ 2_1_ 2	*P*2_1_	*C*2	*P*2_1_ 2_1_ 2_1_
a (Å)	40.19	41.70	70.28	75.99	104.18	61.75
b (Å)	139.40	41.70	146.39	58.10	110.37	135.16
c (Å)	49.19	137.90	58.35	76.27	104.62	147.60
α (°)	90.00	90.00	90.00	90.00	90.00	90.00
β (°)	93.76	90.00	90.00	117.66	119.63	90.00
γ (°)	90.00	90.00	90.00	90.00	90.00	90.00
Content of AU	2	1	2	2	3	2
Resolution (Å)^a^	49.08–1.94 (1.99–1.98)	68.95–1.87 (1.91–1.87)	146.39–2.06 (2.11–2.06)	67.30–1.66 (1.69–1.66)	90.94–2.36 (2.42–2.36)	147.60–2.22 (2.28–2.22)
R_sym_ (%)^a^^,^^b^	4.0 (46.4)	9.1 (109.7)	24.2 (707.9)	5.3 (34.3)	8.6 (125.4)	10.6 (170.4)
I/σ(I)	18.8 (2.1)	12.6 (2.7)	6.7 (0.9)	18.7 (3.3)	7.3 (0.9)	13.2 (1.4)
Completeness (%)^a^	97.9 (96.7)	99.8 (97.2)	100.0 (100.0)	98.5 (85.6)	99.9 (99.8)	100.0 (100.0)
Redundancy^a^	3.4 (3.0)	6.6 (4.8)	12.1 (11.6)	6.0 (4.0)	3.1 (2.5)	11.9 (6.9)
CC_1/2_ (%)^a^	99.9 (70.2)	99.8 (53.6)	99.2 (45.7)	99.9 (87.3)	99.3 (49.4)	99.9 (51.2)
Unique reflections^a^	39,007 (2881)	19,406 (1211)	38,093 (2762)	68,604 (2906)	42,263 (3109)	61,954 (4501)
Wilson B factor	26.1	23.4	31.2	16.1	36.8	38.4
Refinement						
R_cryst_ (%)^c^	16.4	15.3	19.3	19.2	21.3	20.3
R_free_ (%)^d^	21.3	20.1	24.4	23.2	26.1	25.3
RMSD bond length (Å)	0.011	0.010	0.015	0.010	0.015	0.010
RMSD bond angle (°)	1.70	1.64	1.73	1.66	2.21	1.75
Molprobity score	1.43	1.52	1.80	1.49	1.99	2.11
No. of atoms [Average B factor (Å2)]^e^						
Protein	4369 [35.7]	2001 [28.2]	4408 [47.8]	4462 [26.9]	6288 [61.4]	8349 [57.9]
Water	346 [39.3]	127 [34.8]	169 [45.7]	622 [36.9]	174 [48.6]	252 [46.5]
Zn	2 [24.03]	1 [26.4]	2 [53.0]	2 [16.0]	9 [38.13]	2 [50.5]
ADP-ribose	-	36 [20.8]	-	72 [17.5]	-	-
Asn-ADP-ribose	-	-	-	-	132 [34.7]	-
Ramachandran plot						
Favoured (%)	97.4	96.4	96.9	97.3	96.3	96.1
Allowed (%)	2.2	3.6	3.1	2.5	3.2	3.4
Disallowed (%)	0.4	0.0	0.0	0.2	0.5	0.5

a Data for the highest resolution shell are given in parentheses.

b R_sym_ = Σ|/-</>|/Σ/, where / is measured density for reflections with indices *hkl*.

c R_cryst_ = Σ||Fobs| - |Fcalc||/Σ|Fobs|.

d R_free_ has the same formula as R_cryst_, except that calculation was made with the structure factors from the test set.

e Data for the average B factor are given in brackets.

In all structures, the Zn^2+^ ion is tetrahedrally coordinated with three metal-coordination residues within the Zn-loop, while the fourth coordination-contact is exchangeable and is provided by either ADPr, a water molecule, or a symmetry-related protomer within the crystal (Fig. 3*B*). In contrast to the typical pattern of two closely spaced amino acids (functioning as stable coordination base) followed by a zinc-coordinating residue some distance away (50, 51), Zn-Macros have a very short coordination motif of only eight amino acids and hence present an uncommonly compact zinc coordination geometry. His118 (*Sau*Macro) coordinates the Zn^2+^ ion *via* the sterically more demanding, but tighter coordinating, N^δ1^ nitrogen. Interestingly, despite differences in the crystal packing between our (*P*2_1_; PDB 8RSL) and the earlier reported *Sau*Macro apo (*P*2_1_2_1_2_1_; PDB 5KIV) structure, the zinc ions in both structures form crystal contacts with a symmetry-related molecule (Figs. 3*B* and S3*A*). These symmetry contacts complete the first coordination sphere of the Zn^2+^ ion presumably displacing a water molecule, which can be observed in the *Mor*Macro apo structure, where symmetry contacts are absent from the active site (w491; Figs. 3*B* and S3*A*). Note that the observed contacts only occur during crystallization and Zn-Macros and Mfs1 proteins are monomers in solution (see analytical size exclusion chromatography [SEC] and small angle X-ray scattering [SAXS] data below).

ADPr is tightly coordinated within the active site. Within the *Spy*Macro structure, the adenosine base is coordinated *via* an Asp92-adenine C6 amino group contact and Phe251 π-stacks with the base (Fig. S2, *B* and *C*). The proximal ribose is rotated out of the active side and the C3′ position makes a conserved contact with Thr213. The C2′ OH moiety is solvent exposed, but the local environment makes it unlikely that a poly-ADPr chain could be accommodated, thus suggesting that Zn-Macros can only interact with terminal ADPr or mono-ADPr moieties. The ADPr diphosphate is primarily coordinated *via* residues of the—in MacroD-type enzymes—identified diphosphate-binding loop (also termed ‘loop 2’), which is located on the opposite side of the binding cleft relative to the catalytic loop. Comparison with the earlier reported structure of *Sau*Macro revealed distinct differences in loop 2: the symmetry interactions are not equivalent in either structure and takes place *via* His(-3), which is part of a vector-derived sequence (this study; PDB 8RSL) or the side chain of Asp55 (PDB 5KIV), respectively (Fig. S3). These differences have an impact on residues Cys209-Phe218, which overlap with the diphosphate-binding loop region (Thr213-Ala217, *Sau*Macro; Thr269-Gly273, *h*MacroD1; Fig. S2*D*). The diphosphate-binding loop supports ligand binding and can transition between an open and closed confirmation. In contrast, the preceding residues are isostructural between our Zn-Macro and other MacroD-type structures (Figs. 3*C* and S2*D*) but up to 7.7 Å distorted in *Sau*Macro apo (PDB 5KIV). The latter conformation is incompatible with the binding of ADPr, thus indicating that our structure shows a more physiological relevant conformation. This is also confirmed by comparison with the structures of the *Spy*MacroD:ADPr and *Mor*Macro:ADPr complexes, whose loop 2’s are in the closed position and isostructural to earlier reported MacroD-type structures (Figs. 3*C* and S2*D*).

Interestingly, the major difference between the *Spy*Macro and *Mor*Macro complex with ADPr is the form of the distal ribose (Fig. S2, *B* and *C*). In *Spy*Macro, the ribose adopts a furanose ring with a 2′ endo pucker, while the distal ribose in *Mor*Macro is in the linear form. The linear ribose isomer is usually disfavored in solution (52) but has been observed in the Getah virus macrodomain (53). While in the closed sugar *Spy*Macro structure, the distal ribose is coordinated *via* contacts between the C2’’ OH and Asn110 and Asp125 as well as the C1’’ OH and Zn^2+^ ion, the ion contact is lost in the linear ADPr *Mor*Macro structure and C1’’ OH coordinates with Asn101 and Asp116 (isostructural to Asn110 and Asp125 in *Spy*Macro). The open form is further stabilized *via* new contacts with Ala100 as well as the short ^19^SES^21^ stretch in a symmetry-related molecule (Fig. S6).

**Figure 4:**
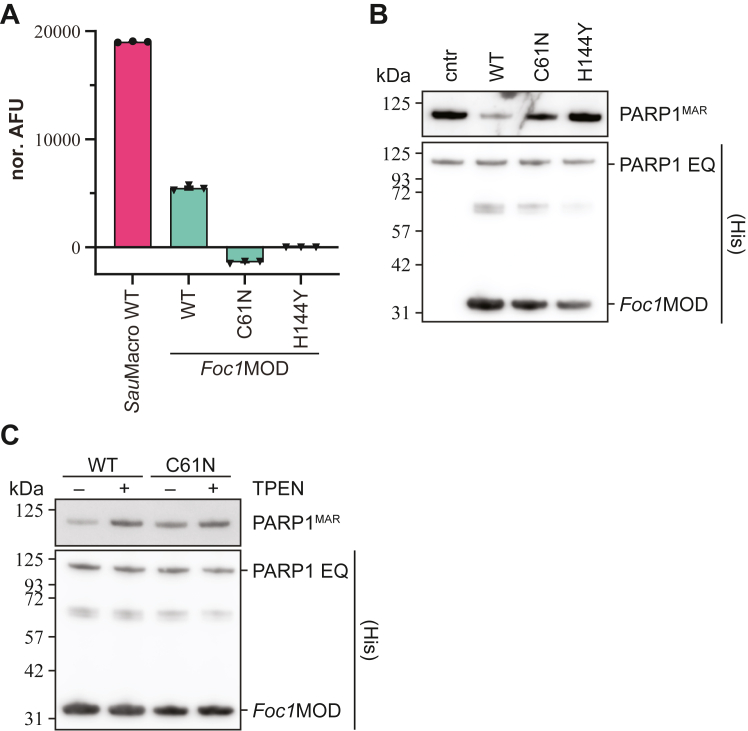
**Zinc-coordination by *Foc1*MOD.***A*, measurement of zinc-content of *Foc1*MOD WT and mutants. Data were background corrected, normalized to *Foc1*MOD H144Y, and represent triplicates ± SD. *Sau*Macro WT was measured as a positive control. *B*, removal of MARylation from human PARP1 E988Q. One micromolar of automodified PARP1 E988Q was incubated with indicated hydrolases. Control reactions were carried out with *Foc1*MOD WT, catalytic inactive H144Y mutant, as well as in the absence of macrodomain hydrolase (cntr). *C*, catalytic activity of *Foc1*MOD WT and C61 N was assessed in presence and absence of 100 μM TPEN (zinc chelator).

In the *Foc1*Mfs1 structure, the Zn^2+^ ion is absent from the macrodomain, while the structural Zn^2+^ ion coordinated within the small subdomain of the SirTM domain is still present (Fig. S4). This absence leads to increased flexibility and distortion of the Zn-loop within the crystal structure as indicated by the loss of density information for the region (residues Asn133 to Ile147). The Zn-loop appears to be partially stabilized by contacts with the 3α-bundle. Among these residues, Arg42, Asn46, and Asn123 (*Sau*Macro) are among the most conserved and make backbone contact to the loop and among each other, thus establishing a tight packing (Fig. S5). In particular, the contacts made by Asn46 seem to contribute to the overall stability of the Zn-loop and the exchange of this residue to cysteine (Cys61 in *Foc1*Mfs1) within the Mfs1 branch may contribute for the absence of the Zn^2+^ ion from the *Foc1*Mfs1 macrodomain structure (Fig. S5*B*). We therefore created macrodomain only construct (*Foc1*MOD; aa 1–305) and compared its zinc-binding ability to *Sau*Macro (Fig. 4*A*). In line with our structural observation, the amount of zinc copurified with the WT *Foc1*MOD protein was reduced by approx. 75% relative to *Sau*Macro. Surprisingly, C61N mutation further decreased zinc affinity to levels of H144Y, which is unable to bind Zn^2+^ ions. However, we observed a weak hydrolytic activity for C61N against Glu-/Asp-ADP-ribosylated PARP1 E988Q (49, 54), which is an established generic MARylation substrate, while H144Y is catalytically inactive (Fig. 4*B*). To determine whether the activity was inherent to the mutant or due to the presence of residual zinc, either copurified or as trace contamination of buffer components, we performed activity assays in the presence of TPEN, a strong zinc chelator (Fig. 4*C*). The presence of TPEN inhibited both WT and C61N, thus suggesting Zn^2+^ ion in the assay facilitate *Foc1*MOD C61N activity. Furthermore, these data suggest that Zn-loop stabilization is a complex and important feature of Zn-Macro activity.

### Zinc coordination is essential for Zn-Macro activity

**Figure 5:**
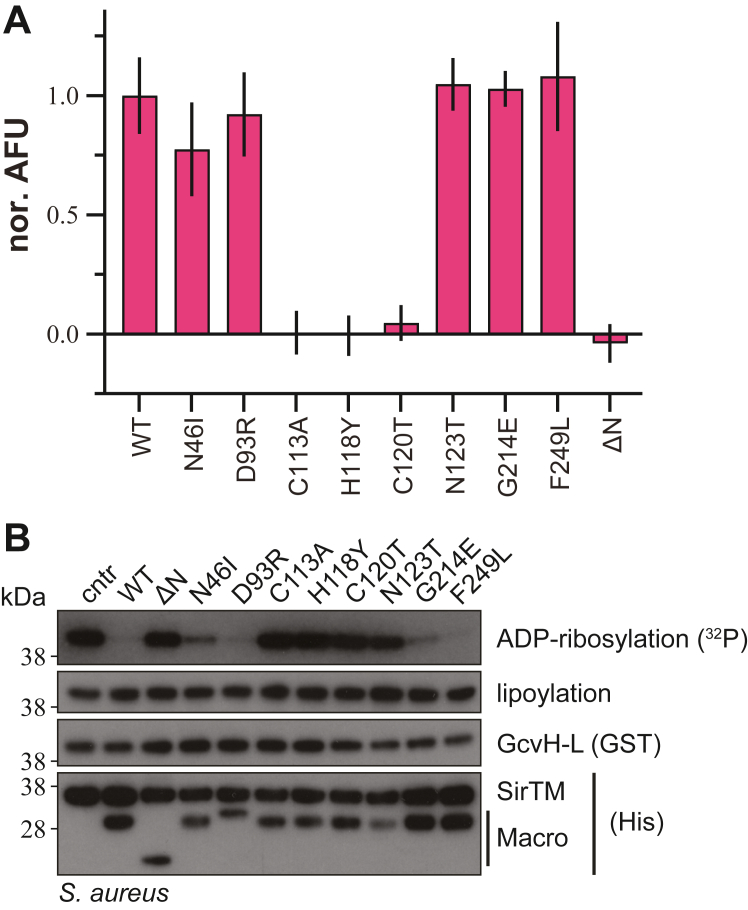
**Zn**^**2+**^**dependence of Zn-Macro activity.***A*, measurement of zinc-content of *Sau*Macro WT and mutants. Data were background corrected, normalized to WT, and represent triplicates ± SD. *B*, removal of ADP-ribosylation on *Sau*GcvH-L by *Sau*Macro. *In vitro* lipoylated *Sau*GcvH-L was MARylated using *Sau*SirTM. One micromolar of modified *Sau*GcvH-L was incubated with WT or mutant *Sau*Macro as indicated. Control reaction was carried out in the absence of macrodomain hydrolase (cntr).

While both the direct interaction between ADPr and the Zn^2+^ ion and our *Foc1*MOD observations are a very strong indications for a catalytic involvement of the zinc ion, we investigated the extent of its functional role by mutation of the adenosine and zinc-coordinating residues as well as deletion of the 3α-bundle using our earlier described *S. aureus* operon system (31). The mutants were analyzed for catalytic activity as well as zinc-incorporation (Fig. 5). Among these mutants, zinc coordination is strictly associated with catalytic activity, thus demonstrating a direct role of the Zn^2+^ ion in catalysis (Fig. 5). Deletion of the N-terminal 3α-bundle (ΔN, aa 1–70) leads also to the loss of zinc-coordination (Fig. 5), which is consistent with our structural observation that the Zn-loop is partially stabilized by the 3α-bundle.

### Differences in the Zn-Macro family

**Figure 6:**
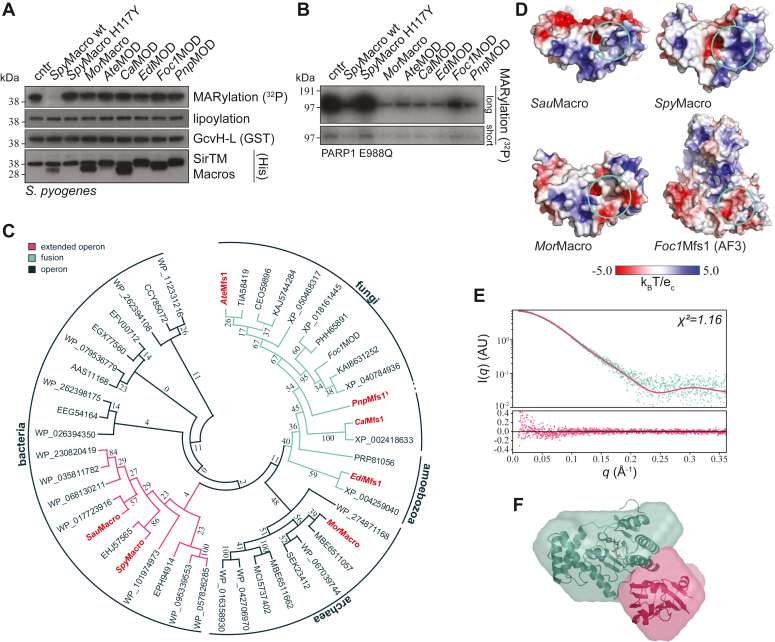
**Analysis of the Zn-Macro family.***A*, removal of ADP-ribosylation on *Spy*GcvH-L by evolutionary diverse Zn-Macros from extended operon, operon, and fusion genetic backgrounds (Fig. 1*A*). *In vitro* lipoylated *Spy*GcvH-L was MARylated using *Spy*SirTM. One micromolar of modified *Spy*GcvH-L was incubated with the indicated macrodomains. Control reactions were carried out with *Spy*Macro WT, catalytic inactive H117Y, as well as in the absence of macrodomain hydrolase (cntr). *B*, removal of MARylation from human PARP1 E988Q. One micromolar of automodified PARP1 E988Q was incubated with indicated hydrolases as well as controls as in (*A*). *C*, evolutionary analysis of Zn-Macros: for tree construction, full-length sequences from SirTM/Zn-Macro pairs or extended operon-encoded Zn-Macros were used. Zn-Macro sequences from fusion proteins were isolated from their whole protein context by multiple sequence alignment. The evolutionary history was inferred by using the maximum likelihood method and LG model of amino acid substitution. The bootstrap consensus tree inferred from 500 replicates is taken to represent the evolutionary history. Branches corresponding to partitions reproduced in less than 50% bootstrap replicates are collapsed. Zn-Macros used in this study are indicated in *red*. Further sequence information are given in Table S3. *D*, visualization of the electrostatic surface potential of *Sau*Macro, *Spy*Macro, *Mor*Macro, and *Foc1*Mfs1 (AlphaFold 3 model). Charge distribution was calculated using APBS (109) as implemented in PyMOL. Approximate position of surface charge conserved in extended operon Zn-Macros is indicated by *green circles*. *E*, SAXS data of *Spy*Macro:lipoyl–*Spy*GcvH-L complex. Experimental scatter overlaid with hypothetical scattering data generated from the predicted complex (*upper* panel) and residual analysis (*lower* panel). *F*, surface reconstructed from SAXS data overlaid with predicted complex structure. *Spy*Macro, *green*; *Spy*GcvH-L, *red*; predicted ligands shown for orientation: Zn^2+^ ion, *gray sphere*; ADP, *black sticks*. See Table S6 for summary of SAXS samples preparation, data collection, and analysis.

To establish whether all Zn-Macro family members have similar substrate specificity, we performed (ADP-ribosyl)hydrolase assays using ADP-ribosylated *Spy*GcvH-L, which is MARylated on Asp27 (31), and PARP1 E988Q, which automodifies on several Glu-/Asp-sites (49, 54), as substrates. As before, *Spy*Macro WT from the extended operon background could remove the ADP-ribosyl modification (Fig. 6, *A* and *B*). Surprisingly, neither *Mor*Macro, which arises from a minimal operon containing only the Zn-Macro/SirTM pair (Fig. 1*A*), nor Zn-Macros derived from Msf1 fusion proteins could demodify *Spy*GcvH-L (Fig. 6*A*). In contrast, all Zn-Macros could demodify Glu-/Asp-ADP-ribosylated PARP1 E988Q (Fig. 6*B*). To determine the molecular basis for this difference, we performed a phylogenetic analysis of the Zn-Macro subfamily and found that the macrodomain sequences cluster according to their taxonomic relationship, with archaeal and fungal sequences forming sister groups. In addition, we observed distinct clades depending on the genetic context of the sequences (extended operon, operon, or fusion protein; Figs. 1*A* and 6*C*, and Table S3). Despite this clustering, we were unable to identify underlying sequence motifs that would explain the observed substrate selectivity. Earlier we observed a strong interaction of the lipoylated GcvH-L protein with the macrodomain of the extended operon (31) and here confirmed it by analytical size-exclusion chromatography (Fig. S7). Apple et al. suggested that the lipoyl moiety would be bound inside a cavity adjacent to the ADPr-binding site (47). However, our data do not support this binding mode: first, the different orientation of the diphosphate-binding loop in our apo structures shortens the cavity, which would sterically hinder the binding of the dithiolane moiety. Second, upon ADPr bind, the cavity is closed due to the reorientation of Phe216 (*Spy*Macro) into the active site to help fix the orientation of the distal ribose within the catalytic centre (Figs. 3*C*, S2*D*, and S6*A* and *B*). Next, we investigated the surface charge distribution among the different macrodomains and found a positively charged surface patch adjacent to the active site present in macrodomains of the extended operon but absent in either fungal or archaeal Zn-Macros (Fig. 6*D*).

**Figure 7:**
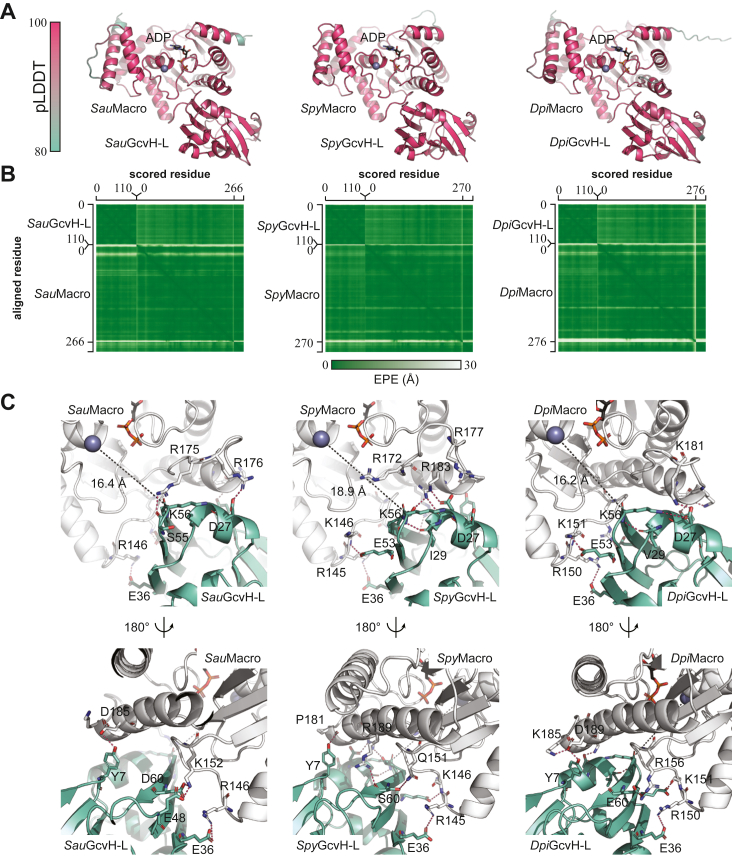
**Structural modeling of Zn-Macro:GcvH-L complexes.** The complexes of *Sau*Macro:*Sau*GcvH-L, *Spy*Macro:*Spy*GcvH-L, and *Dpi*Macro:*Dpi*GcvH-L were modeled using AlphaFold 3 and include one Zn^2+^ ion and one ADP ligand per model. Model quality indicators ipTM and pTM are given in Table S5. *A*, per-residue confidence estimate (pLDDT) mapped onto the predicted structures. The modeled ligands are given as follows: zinc (*gray sphere*) and ADP (*black sticks*), respectively. *B*, expected position error (EPE) of the predicted complexes. *C*, analysis of the interaction surface of the predicted complexes with Zn-Macros shown in *white* and GcvH-L proteins in *green*. Distances between the C^α^ of the lipoyl attachment residues (Lys56) on GcvH-L and the Zn^2+^ ions are given as *black dashed lines*. Selected polar contacts are shown as *dashed lines* (*red*).

Canonical GcvH proteins act—*via* the prosthetic lipoyl moiety—as reaction intermediate carrier proteins between the other components (T-, P-, and L-protein) of the glycine cleavage system (GCV), thus playing a pivotal role in the one-carbon (C1) metabolism (6, 55, 56). While fungal pathogens encode the GCV, not all bacteria do and some species, such as *S. pyogenes*, encode only the extended operon-derived GcvH-L protein (6, 31). Therefore, we analyzed whether surface charge differences between GcvH and GcvH-L proteins could contribute to the interaction, but such differences could not be identified (Fig. S8*A*). All analysed GcvH(-L) proteins show a comparable negative surface charge. However, as we reported earlier, GcvH-L lacks a C-terminal α-helix in comparison to canonical GcvH proteins (Fig. S8, *C* and *D* and Table S4). To investigate whether these features could influence complex formation, we used AlphaFold 3 to model Zn-Macro:GcvH(-L) complexes: (i) from the extended operon of *S. aureus*, *S. pyogenes*, and *Dolosigranulum pigrum*, (ii) Zn-Macro:GcvH complex from *S. aureus* and *D. pigrum*, which both carry a canonical GcvH in addition to the operonal GcvH-L, and (iii) Zn-Macro:*Spy*GcvH-L complexes, with Zn-Macros from *Enterococcus faecalis* and *Treponema pedis* (52.3% [40.7%] and 56.6% [39.5%] sequence similarity [identity], respectively), which are derived from lone SirTM/Zn-Macro pairs. The three Zn-Macro:GcvH-L pairs have very high pTM and ipTM values (>0.9) and show good model-to-model agreement (Figs. 7, S9, S10 and Table S5). In contrast, the prediction confidence of all other models is much lower (pTMs <0.78; ipTMs <0.72), indicating that the overall structures, but not relative subunit positions, are correctly predicted (Fig. S10 and Table S5). This is also reflected by an increased expected position error, decreased pLDDT around the protein:protein interface, and inconsistent GcvH(-L) placement (Figs. 7, *A* and *B*, and S9). We observed that the predicted protein–protein interface in the Zn-Macro:GcvH-L models of *S. aureus*, *S. pyogenes*, and *D. pigrum* involved the positive patches observed in our crystal structures (Figs. 6*D* and 7*C*). However, the GcvH-L placement is inconsistent with a catalytic complex as the residue identified as the ADP-ribosyl acceptor (Asp27) is ∼27 Å from the Zn^2+^ ion (31). On the other hand, Lys56, the lipoyl acceptor residue, is adjacent to the active side. While the interaction is close to the previously predicted site, the above-described cavity is closed in both models. Given the length of the modification and the preference of the Zn^2+^ ion to interact with thiol groups, a direct zinc–lipoyl interaction appears feasible but would involve a nearly linear stretched lipoyl-lysyl moiety. Furthermore, we observe that Lys56 does not contribute to the proposed interaction due to proximity with Arg172, Lys175, and Arg183 (*Spy*Macro) or Arg175 and Arg176 (*Sau*Macro, Fig. 7*C*). An isostructural placement was observed for three of the *Sau*Macro:*Sau*GcvH models. This placement brings the N-terminal helix of GcvH, which is absent in GcvH-L proteins, in close proximity to helix α8 in *Sau*Macro without requiring local conformational changes. In contrast, *Dpi*GcvH is rotated by approx. 90°, thus placing the N-terminal α-helix facing away from *Dpi*Macro (Fig. S10). Among the residues that contribute to the Zn-Macro:GcvH-L complex, we identified Tyr7, Asp27, and Glu36 as conserved among GcvH-L proteins (Figs. 7*C* and S8*D*). However, the contribution of Asp27 indicates that a similar complex might not be able to form when GcvH-L is ADP-ribosylated. To validate these findings, we enriched the lipoyl-modified form of *Spy*GcvH-L by anion exchange chromatography (Fig. S11, Experimental Procedures) and performed SAXS experiments using the *Spy*Macro:lipoyl-*Spy*GcvH-L complex as well as the corresponding monomers (Table S6). The predicted complex conformed well with the observed scatter (Figs. 6, *E* and *F*, and S12). Together our data suggest that (i) a well-defined lipoyl binding site is absent, (ii) the Zn-Macro:GcvH-L interaction occurs primarily *via* electrostatic surface interactions, (iii) the interaction is specific to the extended operon-encoded Zn-Macro:GcvH-L pair, and (iv) the interaction does not represent a catalytic complex.

### Synthesis of *N*-(ADP-d-ribosyl)-asparagine

To gain further insights into the catalytic complex, we endeavored to solve the Zn-Macro:substrate complex. Given the intrinsic linkage between Zn^2+^ ion coordination and catalytic activity, we were unable to crystallize our protein with Asp-ADPr–modified GcvH-L or a peptide containing the modification. Therefore, we synthesized asparagine-ADP-ribose (Asn-ADPr) as close, nonhydrolyzable isostere for structural studies.

**Figure 8:**
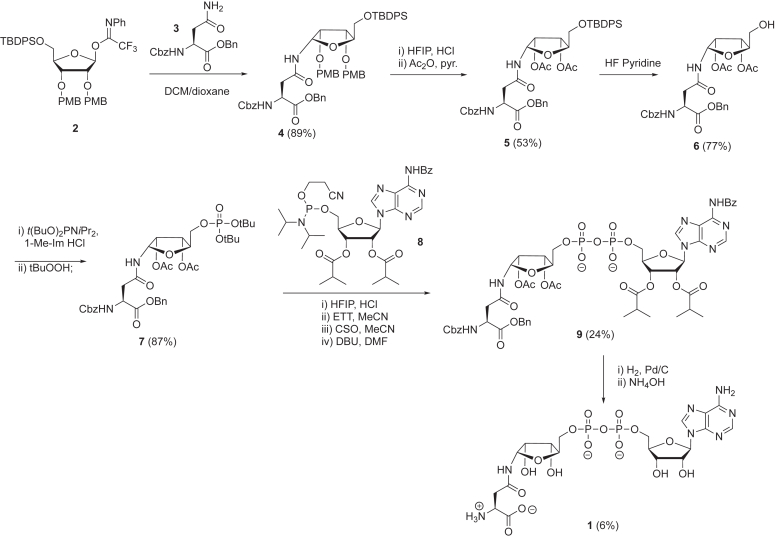
**Synthesis of Asn-ADPr** (1).

The synthesis of Asn-ADPr 1 started with the preparation of an orthogonally protected ribosylated Asn building block 6 and was followed by the introduction of pyrophosphate at the 5-OH of the ribose moiety in 6 using P(V)-P(III) chemistry (Fig. 8) (57, 58). First, compound 4 was prepared *via* coupling of known trifluoroacetimidate ribofuranose donor 2 (59) with the carboxamide in Cbz-Asn-OBn (3) under the influence of TBSOTf as an activator in a mixture of dichloromethane and 1,4-dioxane. The glycosylation of 3 with 2 proceeded in complete α-stereoselective fashion to furnish the desired *N*-ribosylasparagine derivative 4 in a good yield. Subjection of 4 to 0.1 equivalent HCl in HFIP (hexafluoro-2-propanol) (58, 60) removed both para-methoxybenzyl protections and subsequent acetylation of the resulting diol-furnished α-anomer 5. This acidolysis was accompanied by minimal epimerization to the β-anomer of 5 that was removed by silica gel column chromatography. Next, the 5-OH of compound 5 was liberated by HF-pyridine mediated desilylation to obtain 6. Compound 6 was converted into phosphotriester 7 in a high yield by the treatment with *tert-*butyl protected phosphoramidite [(*t*BuO)_2_PN*i*Pr_2_] and activator 1-methylimidazolium chloride (61) followed by oxidation with *t*BuOOH. Both tert-butyl (*t*Bu) protecting groups in phosphotriester 7 were rapidly cleaved by the treatment with HCl/HFIP. The obtained crude phosphomonoester was coupled with phosphoramidite 8 under the activation of 5-(Ethylthio)-1H-tetrazole (ETT), followed by oxidation mediated by (1*S*)-(+)-(10-camphorsulfonyl)-oxaziridine. Subsequent treatment with diazabicycloundecene (a strong organic base) removed cyanoethyl group to furnish partially protected pyrophosphate 9, which was purified by column chromatography and gel filtration on Sephadex LH-20. The complete removal of the benzyl (Bn) and benzyloxycarbonyl (Cbz) groups was achieved by Pd/C-catalyzed hydrogenolysis of 9 for 48 h, as evidenced by LC-MS analysis. The acetyl (Ac) and benzoyl (Bz) groups in the obtained crude intermediate were removed by treatment with NH_4_OH for 24 h to give highly hydrophilic final compound 1 in a 6% yield after purification by HW-40 gel filtration and, subsequently, ion exchange column chromatography.

### Substrate and ligand binding of Zn-Macros

**Figure 9:**
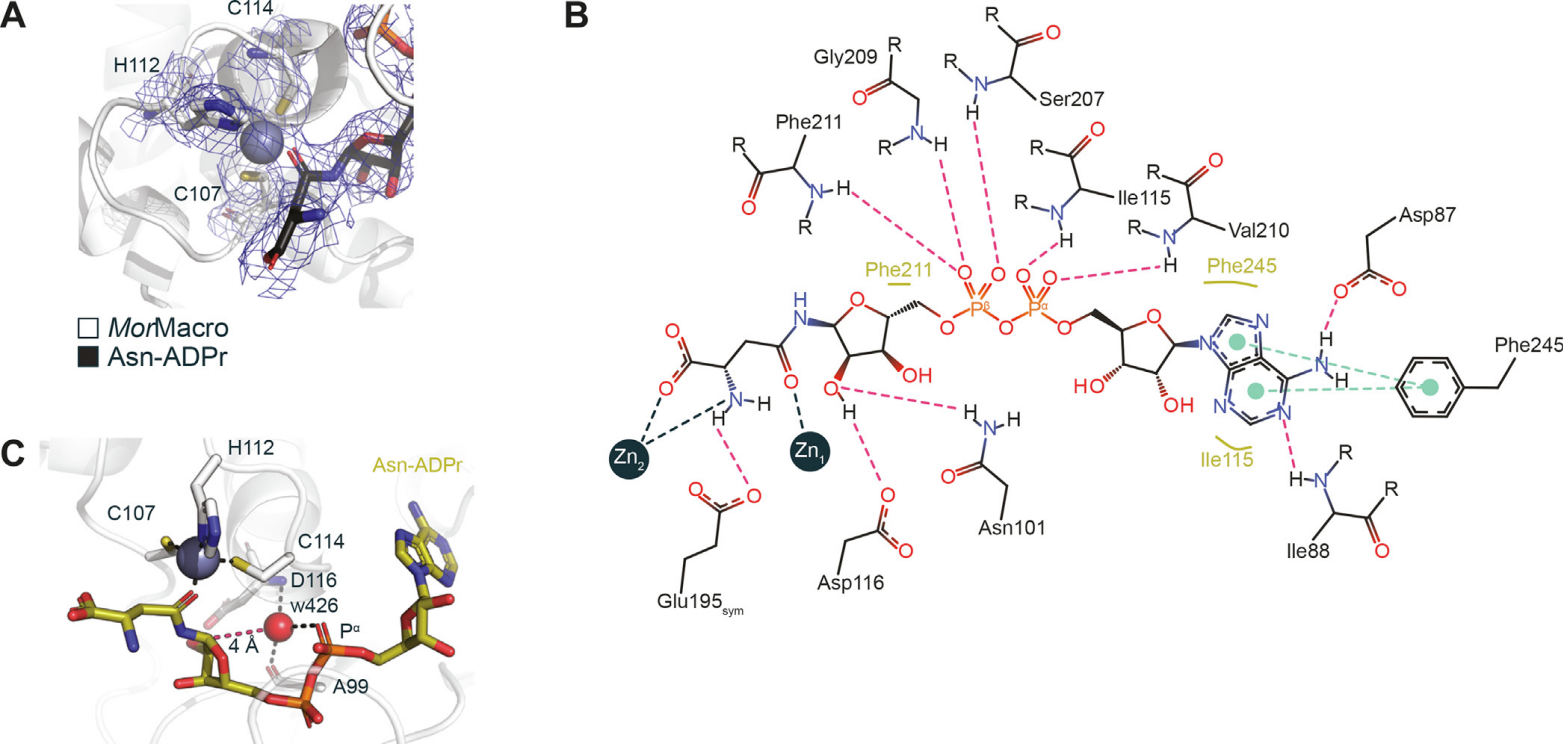
**Coordination of the substrate analog Asn-ADPr within the active site.***A*, electron density omit map (2Fo-Fc contoured at 1 σ) refined in the absence of any ligand. The final refined protein–ligand structure is shown as reference. *B*, 2D ligand interaction diagram of Asn-ADPr coordinated within the active site of *Mor*MOD. Polar and π-π interactions are indicated by *red* and *green dashed lines*, respectively, and hydrophobic contacts by *yellow lines*. Zn_1_ indicates the macrodomain bound zinc ion, while Zn_2_ refers to an additional Zn^2+^ ion only observed in this structure. The diagram was generated using PoseEdit (https://proteins.plus). *C*, ribbon-liquorice representation of w426 coordination within the active site of the *Mor*MOD:Asn-ADPr complex. Polar contacts are given as *black dashed lines* and the w426-C1’’ (Asn-ADPr) distance as *red dashed line*. Zn_1_ coordinated by the Zn-loop is given for orientation.

Having synthesized Asn-ADPr, we were able to crystallize the *Mor*Macro:Asn-ADPr complex (Table 1). The overall structure closely resembles the ADPr-bound form (RMSD 0.515 Å over 245 C^α^). The ADPr moiety placement is isostructural to our *Spy*Macro:ADPr complex with zinc(II) contact made by the N^δ2^ atom of the modified Asn side chain, which again leads to a tetrahedral coordination of the Zn^2+^ ion (Fig. 9*A*). Interestingly, the crystal packing appears to be stabilized by two additional, nonphysiological zinc ion complexes: (i) a protomer-protomer contact is created by an octahedrally coordinated zinc ion bound by Asn-ADPr and Glu195 from the neighbouring protomer (linking A-B, B-C, and C-A) and (ii) a symmetry contact is created by the tetrahedral coordination of a zinc ion *via* Glu72, His239, Glu72_sym_, and His239_sym_ (linking A-A_sym_, B-B_sym_, and C-C_sym_); Fig. 9*B* and S13). Comparison with the *Mor*Macro structures lacking these contacts shows that they have no discernible influence on the local or overall protein conformation.

Earlier studies on MacroD-type enzymes identified two well-ordered water molecules in the vicinity of the active side: w426 (*Mor*Macro) coordinated between the α-phosphate, distal ribose, and α-helix following the catalytic loop (helix α6 in human MacroD2 [PDB 4IQY] and helix α2 in SARS-CoV-2 macrodomain 1 [PDB 7KQP]) and the second, also termed w_PHOS_, between the α-phosphate and the distal ribose (Fig. 9*C*). In none of our structures, a water molecule isostructural to w_PHOS_ could be observed and no new close-distant water could be identified. The similarities of w426 coordination among known MacroD-type structures suggest either a structural or catalytic role. Recent ultra-high resolution and proton scattering data of the SARS-CoV-2 macrodomain 1 in complex with ADPr showed that w8 (isostructural to w426; PDB 7KQP) is ideally positioned for a colinear nucleophilic attack on the sp^3^ centre of the C1’’ (62). Moreover, the C1’’ centre appears to be strongly positively polarized due to the direct substrate coordination by the Zn^2+^ ion as well as the coordination of the 2’’ OH group by a conserved histidine-aspartate dyad (Asp116 and His120 in *Mor*Macro; Figs. 9, *B* and *C*).

## Discussion

ADP-ribosylation is, like most posttranslational modifications, a transient signaling event, and the intricate interplay of establishing transferases and erasing hydrolases is a crucial factor of the physiological outcome, determining parameters such as response intensity and signal duration.

### Evolution and function of Zn-Macros

**Figure 10:**
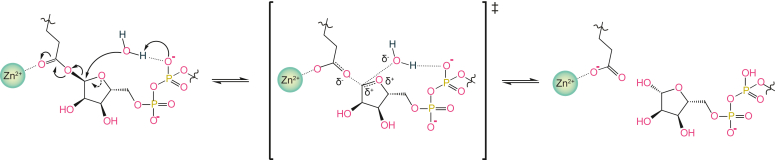
**Substrate-assisted reaction mechanism of Zn-Macros.** Upon substrate binding, the Zn^2+^ ion of the macrodomains can act as Lewis acid and induce polarization of the glycosidic bond *via* coordination of the acetal ester, while at the same time, the P^α^ phosphate acts as Lewis base to abstract a proton from a coordinated water molecule (w426; Fig. 9*C*). This concerted acid/base action allows a nucleophilic attack at the ribose anomeric centre. In the transition state, the oxygen adjacent to the reaction centre can stabilize the developing positive charge with one of its lone electron pairs leading to the formation of an oxocarbenium ion-like species.

To elucidate the physiological role of dynamic ADP-ribosylation signaling, a mechanistic understanding of the enzymes involved is required. Today, three superfamilies of ADPr reversal enzymes have been identified, (i) (ADP-ribosyl)hydrolases, (ii) macrodomains, and (iii) *NAD* and *A*DP-*r*ibose linked hydrolases (36, 37, 63, 64). These superfamilies are distinguished by their individual folds, modes of substrate binding, and catalytic mechanisms and present unique solution to perform partially overlapping function, for example, both macrodomains and ARHs evolved to hydrolyze a variety of different linkages on proteins and nucleic acids including Arg- and Asp/Glu-ADPr linkages on proteins (37, 65, 66, 67, 68, 69, 70, 71). This divergence can also be observed within superfamilies, especially the macrodomains, which serve both as hydrolases as well as nonenzymatic ADPr “reader” domains (36). However, even within the superfamilies, catalytic mechanisms can be divergent. Among the macrodomains, the MacroD-type family has come into particular focus as it is conserved in all branches of life as well as several viruses, shows mechanistic plasticity, and members have high potential as therapeutic targets (14, 15, 25, 44, 72, 73, 74, 75). We previously showed that viral and human MacroD-type macrodomains differ in key catalytic residues with human MacroD1/2 showing a GGGxDx_3_H, coronavirus nsp3 MOD1 a HGGG, and alphavirus nsp3 MOD1 a GxGxC motif (Fig. S1) (44). The here described Zn-Macro subfamily also carries a conserved Dx_3_H motif, however, has replaced the preceding catalytic loop with a catalytic zinc-binding motif (Figs. 2*B* and S1). This is indicative for an evolution from a MacroD1/2-like ancestor. While the exact mechanism of ADPr removal remains elusive, the substrate and water coordination exhibits communalities with *β*-glycosidase employing an inverting mechanism (76, 77, 78). These glycosidases have two carboxyl groups within their active site, which act as general acid and base during the reaction. The acid can donate a proton to the leaving group, while the base abstracts a proton from a water molecule, which in turn attacks the anomeric center of the sugar. The steps are carried out in a concerted manner, which leads to the formation of a transition state with strong oxocarbenium ion-like character. The absence of a free oxocarbenium ion species is interesting as oxocarbenium ions are believed to be too unstable to exist as a free intermediate without further stabilization (79). In contrast to glycosidases, Zn-Macros have only the carboxyl group of the classic Asp/His dyad within the active site (Figs. 2*B* and S1). While the aspartate induces polarization at the anomeric C1’’ center, this occurs *via* coordination of the 2’’OH moiety and not the acetal ester. However, the Zn^2+^ ion interacts with the latter and, due to its filled *d* orbital (d^10^) and thus stable oxidation state, can act as Lewis acid during catalysis (80, 81). With regard to the catalytic base, extensive structural studies on MacroD-type hydrolases in their ligand-bound state revealed consistently the presence of a water molecule interaction with the ADP-ribose α-phosphate, which was suggested to participate in achieving a strained substrate conformation (36, 44, 69). Recently, a detailed investigation of the SARS-CoV-2 nsp3 MOD1 macrodomain showed that this water molecule is ideally orientated for a nucleophilic attack on the C1’’ center, which suggest a dual—structural and catalytic—role (62). We could observe an isostructural water molecule in our structures of *Spy*Macro and *Mor*Macro in complex with ADPr and Asn-ADPr, respectively, thus suggesting that the P^α^ phosphate group could act as Lewis base in the reaction (Figs. 9*C* and 10). Note, Zn-Macros lack residues involved in the stabilization of an oxocarbenium ion intermediate at the distal ribose, hence suggesting either a mechanism without formation of such an intermediate or—like in glycosidases—a transition state with an oxocarbenium ion-like character without forming the free species. While further investigations are needed to establish the detailed mechanism, the here described features strongly suggest a substrate-assisted, acid-base–catalyzed mechanism with inversion at the anomeric centre and oxocarbenium-like transition state (Fig. 10). The unique mode of substrate activation and presence of the zinc(II) ion has also a profound impact on the substrate range. Where other MacroD-type macrodomains are limited to *O*-glycosidic bonds involving acidic sidechains, Zn-Macros are the only known hydrolases able to cleave the *S*-glycosidic bond of modified cysteines (48). The latter reaction is likely supported by the readiness of the Zn^2+^ center to form thiolate complexes, whereas in other MacroD-type macrodomains, the thiolate is a poorer leaving group than a carboxylate.

It is interesting to note that beyond the bond specificity, Zn-Macros appear to differ in their protein substrate selectivity. While Zn-Macros encoded by the extended operon can remove the ADP-ribosyl modification from GcvH-L, Zn-Macros encoded as Zn-Macro/SirTM pairs or fusion proteins cannot, thus suggesting that the microenvironment of the ADP-ribosyl modification contributes to the hydrolysis reaction. Our data support the idea that the lipoyl modification contributes to the Macro:GcvH-L interaction *via* the removal of the positive lysine charge. However, the interaction in the absence of the ADP-ribosyl modification does not position the potentially modified aspartate (Asp27) close to the active site and GcvH-L can still be demodified in the absence of the lipoylation mark. This suggests that the lipoylation contributes to the overall binding energy, for example, by removing the positive lysine charge from the interaction surface but is not the only determinant. On the other hand, the absence of positive interaction surfaces in the other family members of the Zn-Macro family (having neutral to negative surfaces) might provide an energetic barrier to GcvH-L binding and explain the absence of activity from these macrodomains. In addition, our assays utilizing MARylated human PARP1 E988Q, which is primarily modified on glutamate site with only minor aspartate contribution (49, 54), may suggest that glutamate residues with their slightly longer side chain are more easily accommodated within the active site. Identifying the physiological substrates for example, for the Mfs1 fusion protein would not only elucidate their substrate specificity in terms of attachment residue and sequential context but also reveal how these proteins contribute to the oxidative stress response of pathogenic fungi.

### Physiological role and therapeutic potential

The different substrate specificities among the Zn-Macros hint at distinct roles in regulatory pathways. That said, our previous findings suggest that SirTM/Zn-Macro–regulated ADP-ribosylation signaling plays a role in the oxidative stress response of species carrying the extended operon and fusion enzyme varieties (31). Support for this idea comes from transcriptomic and proteomic data showing a strong upregulation of the operon or fusion protein upon oxidative stress (82, 83, 84) as well as the association of this modification system with two oxidoreductases (one luciferase-like monooxygenase and one old yellow enzyme-type (OYE); Fig. 1*A*). Moreover, a recent study showed that *S. aureus* OrfA, a close homolog to the operon-encoded OYE (also termed OrfB), is important for thiol-dependent redox homeostasis (85). Together with our earlier observation that GcvH-L and OYE interact in a lipoylation-dependent manner (31), this hints at the possibility that the lipoyl modification can act as a redox scavenger. In this model, ADP-ribosylation of GcvH-L would prevent its participation in reactive oxygen species (ROS) detoxification, while removal of this modification by the macrodomain allows contribution of the target protein in ROS scavenging (31). The generation of ROS is one of the main defence mechanisms of the innate immune response and crucial for the early clearance of pathogens. As Zn-Macros have structurally distinct features and are primarily encountered in pathogenic microorganisms, they might, therefore, present a novel therapeutic target for infections caused by these organisms. The continued rise in AMR makes it paramount to explore novel strategies to combat both bacterial and fungal infections. Investigation into the exact physiological role of the Zn-Macro family could help to better understand microbial ROS evasion/detoxification and lead to new therapeutic strategies.

## Experimental procedures

### Plasmid construction

Expression vectors for *Sau*GcvH-L, *Sau*LplA2, *Sau*SirtM, *Sau*Macro, *Spy*GcvH-L, *Spy*LplA, *Spy*SirTM, *Spy*Macro, and PARP1 E988Q were described previously (31, 86). For crystallization, *Spy*Macro was cloned into pET9H_3_ (31) *via* NcoI/BamHI (vector) and PciI/BamHI (*Spy*Macro) restriction sites. Protein sequence of *M. oralis* macrodomain (WP_042691995) was transformed into the coding sequence and codon optimized for expression in *Escherichia coli* (K12 strain) using the JCat web tool (87). The resulting coding sequence including PagI and BamHI restriction sides was gene synthesized in pUC58 (Biomatik). Subsequently, the gene was transferred into pET9H_3_ for expression using NcoI/BamHI (vector) and PagI/BamHI (*Mor*Macro) restriction sites. Coding sequences for Mfs1 fusion proteins were amplified from genomic DNA (strains: *Aspergillus terreus*, IMI35576; *Candida albicans*, SC5314; *Entamoeba dispar*, SAW760; *F. oxysporum* f.sp. *cubense* race 1, IMI141109, and *Phytophthora nicotinae* var. *parasitica*, IMI403522) and cloned into pET28a (*Cal*Mfs1), pET21a (*Edi*Mfs1), or pET9H_3_ (*Ate*Mfs1, *Foc1*Mfs1, and *Pnp*Mfs1). Differences in codon usage in the *Cal*Mfs1 sequence were corrected for expression in *E. coli* by site-directed mutagenesis. The macrodomain coding sequences (*Ate*Macro [aa 1–305], *Cal*Macro [aa 1–281], *Edi*Macro [aa 1–304], *Foc1*Macro [aa 1–305], and *Pnp*Macro [aa 1–297]) were PCR-amplified and cloned into pET21a using NdeI/BamHI restriction sites. Point mutations, deletions, and sequence exchanges were introduced using site-directed mutagenesis.

### Protein expression and purification

#### For biochemistry

Recombinant proteins were expressed in Rosetta (DE3) cells grown in lysogeny broth medium supplemented with 2 mM MgSO_4_ and appropriate antibiotics at 37 °C to A_600_ 0.6. Expression was induced with 0.4 mM IPTG and 5 μM zinc acetate in case of zinc-containing enzymes. Cells were grown at 30 °C and harvested 4 h postinduction by centrifugation (4500×*g* for 15 min at 4 °C). Cell pellets were resuspended in lysis buffer (50 mM TrisHCl [pH 8], 500 mM NaCl, 10 mM imidazole) and stored at −20 °C until use. Recombinant His-tagged proteins were purified by Ni^2+^-NTA chromatography (Serva Electrophoresis GmbH) according to the manufacturer's protocol using the following buffers: all buffer contained 50 mM TrisHCl [pH 8] and 500 mM NaCl; additionally, the lysis/binding buffer contained 10 mM imidazole, the washing buffer contained 30 mM imidazole, and the elution buffer contained 500 mM imidazole. Eluted proteins were dialyzed against storage buffer (50 mM TrisHCl [pH 8], 200 mM NaCl, 1 mM DTT, 5% (v/v) glycerol) overnight at 4 °C and stored at −80 °C until use. For interaction study, *Sau*GcvH-L was lipoylated *in vivo* as described before (31). Briefly, Rosetta (DE3) cells were grown as described above and upon induction, supplemented with 100 μM lipoic acid and grown for 3 h at 30 °C. Further protein synthesis was inhibited by the addition of 150 μg/ml kanamycin and cells were incubated for an additional 60 min at 30 °C before harvesting and processing as described above.

PARP1 E988Q was transformed in Rosetta (DE3) cells grown in 2xYT medium supplemented with 10 mM benzamide and expression was induced at A_600_ 0.6 with 0.4 mM IPTG. Cells were grown overnight at 17 °C and harvested by centrifugation (4500×*g* for 15 min at 4 °C). Pellets were resuspended in lysis buffer (25 mM Hepes [pH 8], 500 mM NaCl, 0.5 mM TCEP) and lysed by high-pressure homogenization. The protein was purified using an ÄKTA FPLC system (Cytiva) at 4 °C *via* affinity chromatography using a HisTrap HP column (Cytiva). After lysate application, the column was washed with lysis buffer supplemented with, first, 50 mM imidazole and, second, NaCl to a total concentration of 1 M, followed by the elution of bound protein with the addition of 250 mM imidazole. The eluate was diluted fivefold with 25 mM TrisHCl [pH 7], 100 mM NaCl, 0.5 mM TCEP and applied to a HiTrap Heparin column (Cytiva) equilibrated in the same buffer; the protein was eluted with a linear NaCl gradient from 100 mM to 1000 mM. Fractions containing PARP1 E988Q were pooled and loaded on a Superdex 200 Increase 10/300 Gl and eluted with 25 mM Hepes [pH 8], 100 mM NaCl, 0.2 mM TCEP. Fraction containing PARP1 E988Q were pooled and stored at −80 °C until use.

#### For structural analysis

Proteins for crystallization were expressed as described above. Cell pellets were thawed overnight on ice, supplemented with benzonase and lysozyme, and incubated for 1 h at 4 °C on a rotating wheel. Cells were subsequently lysed by high-pressure homogenization. Lysate was clarified by centrifugation (35,000×*g*, 50 min, 4 °C) and purified using an ÄKTA FPLC system (Cytiva) at 4 °C *via* affinity chromatography using a HisTrap HP column (Cytiva). Affinity-tags of *Foc1*Mfs1, *Mor*Macro, and *Spy*Macro were removed by proteolytic cleavage using HRV3C protease during dialysis overnight against lysis buffer at 4 °C. Uncleaved proteins and protease were removed by passing the protein over a GSTrap HP (Cytiva) and HisTrap HP affinity-column (Cytiva). His-affinity purified *Sau*Macro as well as cleaved *Foc1*Mfs1, *Mor*Macro, and *Spy*Macro were further purified by SEC on a HiLoad Superdex 75 pg and eluted with crystallization buffer (*Foc1*Mfs1: 10 mM Pipes [pH 7], 75 mM NaCl, 1 mM TCEP; *Mor*Macro: 10 mM TrisHCl [pH 8], 100 mM NaCl, 1 mM DTT; *Spy*Macro: as *Mor*Macro containing 1 mM aspartic acid; *Sau*Macro: 15 mM TrisHCl [pH 8], 125 mM NaCl, 1.5 mM DTT) or SAXS buffer (25 mM TrisHCl [pH 8], 150 mM NaCl, 2.5 mM TCEP, 3% (v/v) glycerol). Fraction containing target proteins were concentrated and stored at −80 °C until use.

*Sau*GcvH-L and *Spy*GcvH-L for interaction study and SAXS analysis was lipoylated *in vivo* as described for *Sau*GcvH-L above. Cell pellets were resuspended in ice cold lysis buffer containing benzonase and lysozyme and incubated for 1 h at 4 °C on a rotating wheel. GcvH-L containing fraction were pooled and diluted to approximately 30 mM NaCl content using buffer A (50 mM TrisHCl [pH 8]) and loaded onto a HiTrap Q column (Cytiva). Protein was eluted using a 3 to 100% gradient of buffer A and B (50 mM TrisHCl [pH 8], 1 M NaCl). Gradient was manually interrupted at approximately 12.5% B to allow for better separation of nonmodified and lipoylated GcvH-L. Finally, lipoylation status of the eluted proteins was verified by immunoblot and lipoyl-GcvH-L was pooled and dialyzed against SAXS buffer and stored at −80 °C until use.

### Immunoblot

Enzymatic reactions stopped with NuPAGE LDS sample buffer (Invitrogen) containing 1 mM DTT (see below) were electrophoretically separated on NuPAGE Novex 4 to 12% Bis-Tris gels (Invitrogen) and transferred to nitrocellulose membranes (Bio-Rad) for 10 min using Trans-Blot Turbo Transfer System (Bio-Rad). The blotted membranes were blocked with PBS buffer containing 0.1% (v/v) Tween 20 and 4% (w/v) skimmed milk powder (Marvel, Premier Foods plc) for 1 h at RT and then incubated with mouse monoclonal anti-6xHis antibody (Clontech, 631212; RRID: AB_2721905), HRP-conjugated goat polyclonal anti-GST antibody (ab58626, Abcam, RRID: AB_880249), rabbit polyclonal anti-lipoic acid antibody (437695, Calbiochem; RRID: AB_212120), or rabbit anti-monoADPr anti reagent (MABE1076, Millipore, RRID: AB_2665469) over night at 4 °C. After washing with PBS containing 0.1% (v/v) Tween 20, the blots were incubated with a horseradish peroxidase–labeled anti-rabbit IgG (P0399, Dako, RRID: AB_2617141) or anti-mouse IgG (P0447, Dako, RRID: AB_2617137) for 1 h at RT. Detection was performed using Pierce ECL Western blotting substrate (Thermo Fisher Scientific) and analysed by luminography using either Hyperfilm ECL (Amersham) or ChemiDoc MP (Bio-Rad).

### Enzymatic assay

Lipoylation of GcvH-L was carried out in lipoylation buffer (50 mM TrisHCl [pH 8], 200 mM NaCl, 5 mM ATP, 2.4 mM lipoic acid, 1 mM MgCl_2_, 1 mM DTT) using 2 μM LplA and 4 μM GcvH-L. Reactions were incubated for 30 min at 30 °C. Subsequent, ADP-ribosylation was carried out by the addition of 2 μM SirTM in MARylation buffer (50 mM TrisHCl [pH 8], 200 mM NaCl, 1 mM MgCl_2_, 1 mM DTT) containing 1 μCi ^32^P-NAD^+^ and 5 μM unlabeled NAD^+^ so that GcvH-L concentration was decreased to 2 μM. Reactions were incubated at 30 °C for 60 min. For de-ADP-ribosylation, 1 μM radiolabeled GcvH-L was incubated with 1 μM macrodomain in MARylation buffer for 1 h at 30 °C. Reaction were stopped by the addition of LDS sample buffer and analyzed by immunoblot and autoradiography.

Auto-MARylation of PARP1 E988Q was carried out in PARP buffer (50 mM TrisHCl [7.5], 50 mM NaCl, 4 mM MgCl_2_, 0.2 mM DTT) containing 0.5 μCi ^32^P-NAD^+^, 10 μM unlabelled NAD^+^, and activated DNA (Trevigen) using 1 μM enzyme for 30 min at 30 °C. The reaction was stopped by the addition of 10 μM olaparib. De-modification was carried out by incubation 0.5 μM radiolabeled PARP1 E988Q with 1 μM macrodomain in MARylation buffer for 1 h at 30 °C. Reactions were stopped by the addition of LDS sample buffer and analyzed by immunoblot and autoradiography.

### Zinc content analysis

To measure the release of Zn^2+^ ions from the recombinant protein, concentrations of WT and mutants proteins were equalized to 20 μM with dilution buffer (50 mM TrisHCl [7.5], 200 mM NaCl, 1 mM DTT). To 100 μl protein dilution were added 80 μl of denaturation buffer (50 mM TrisHCl [pH 7.5], 200 mM NaCl, 1 mM DTT, 5% (w/v) SDS) and 20 μl diluted zinpyr-1 (100 μM in 10% (v/v) DMSO). Proteins were denatured by incubation at 95 °C for 10 min, before rapid cooling on ice. Samples were transferred in to a black 96-well plate and fluorescence signals were recorded on a SpectraMax M5 plate reader (Molecular Devices) and data analyzed with GraphPad Prism (v10.0.2). All samples were measured in triplicates and background corrected against a buffer-only control.

### Analysis of *Sau*Macro:*Sau*GcvH-L interaction by SEC

To analyze the *Sau*Macro:*Sau*GcvH-L complex, proteins were diluted either alone or as 1:1.2 M mixture to 50 μM *Sau*Macro and 60 μM *Sau*GcvH-L with TZNK/D buffer (50 mM TrisHCl [pH 8], 150 mM KCl, 12 mM NaCl, 100 μM zinc acetate, 2 mM MgCl_2_, 5 mM DTT) and loaded onto an Superdex 200 Increase 10/300 Gl (Cytiva) equilibrated with the same buffer. Eluted fractions were analysed by immunoblot.

### Chemical synthesis of Asn-ADPr

The synthesis methodology utilized for synthesizing *N*-(ADP-d-ribosyl)-asparagine (Asn-ADPr) was developed on the basis of previously described chemistries (59). Further details of the synthesis, variations to the original protocol, as well as analytical data are given in the Supporting Information.

### Small angle X-ray scattering

Experimental and analysis parameters are summarized in Table S6. Briefly, *Spy*Macro and *Spy*GcvH-L were purified as described above and analyzed either alone or as a 1:1.2 M complexes. All samples were filtered using a 0.22 μm filter column (Ultrafree, Durapore polyvinylidene fluoride membrane) before SAXS measurements were performed using the SEC configuration: capture of the elution peak in a 1.5 mm quartz capillary flow cell (1.6 mm path length) and data collected on an Eiger 4M detector (Dectris). The X-ray wavelength and sample-to-detector distance were 1.024 Å and 4.04 m, respectively, corresponding to an accessible *q*-range of 0.0045 to 0.34 Å^-1^. SEC was achieved with an Agilent 1200 series high-pressure liquid chromatography and a Shodex silica resin KW402.5-4F column equilibrated with three column volumes of SAXS buffer before each injection. During the elution, SAXS measurements were made using 3-s exposure frames. Data were acquired and reduced using the general data acquisition software (DLS) and DAWN Science [DLS; (88)], respectively. Data were analyzed using ScÅtter (Bioisis) and MULCh [University of Sidney; (89)], and *ab initio* shapes were determined using MONSA as integrated into the ATSAS program suit [EMBL; (90, 91)].

### Crystallization and X-ray data collection

Crystallization procedures for all here reported structures are summarized in Table S7. All proteins were purified as described above and set up in sitting drop SwissCi (MRC) 96-well 2-drop plates (SPT Labtech) using a 1:1 mother liquor (ML) to protein ration. Crystals were grown at 292 K within 7 days if not stated otherwise. For crystal seeding, initial crystals were crushed using the Seed Bead kit (Hampton Research) according to manufacturer’s recommendations. Crystals were grown from seed using a 5:4:1 ratio of ML:protein:crystal seed. Crystals of *Mor*Macro and *Spy*Macro were cryoprotected by submersion in 18% (v/v) ethylene glycol in ML for 5 s. Similarly, *Sau*Macro was cryoprotected with 18% (v/v) glycerol in ML. All crystals were vitrified in liquid nitrogen.

### Structure determination and analysis

X-ray diffraction data were collected using synchrotron radiation at Diamond Light Source and at the European Synchrotron Radiation Facility (Table 1). Diffraction images were processed using the XIA2 platform (92). All subsequent crystallographic calculations were performed with the CCP4 software package (93). Phase information were determined using the molecular replacement method as implemented in PHASER (94). Density modification was carried out in PARROT (95), and initial models were constructed using the automated building program BUCCANEER (95). To refine the atomic models, successive cycles of manual building were undertaken in COOT (96) with structure refinement carried out using REFMAC5 (97). Structures were validated using MolProbity (98) and Ramachandran statistics. Detailed processing and refinement statistics can be found in Table 1. Structural alignments, analyses, and figure preparation were conducted using PyMol (Molecular Graphics System, Version 2.3.3, Schrödinger, LLC) and 2D ligand interaction diagrams were created with PoseEdit as implemented in Proteins*Plus* (https://proteins.plus) (99, 100).

### AlphaFold 3: structural modeling

Zn-Macro:GcvH(-L) complexes as well as the full-length *Foc1*Mfs1 structure were modeled using the AlphaFold 3 server (https://golgi.sandbox.google.com) (101). The input sequences were obtained from GenBank *Dpi*GcvH-L (EHR34890), *Sau*GcvH-L (WP_000731878), *Spy*GcvH-L (WP_002984553), *Dpi*Macro (EHR34889), *Efa*Macro (EPH94914), *Sau*Macro (WP_000449069), *Spy*Macro (WP_011888850), *Tpe*Macro (WP_024467805), *Dpi*GcvH (EHR32225), and *Sau*GcvH (WP_000290491) or directly determined in this study *Foc1*Mfs1 (OR133608). In addition, the following ligands were included into the models (i) Zn-Macro containing complexes: one Zn^2+^ ion and one ADP ligand and (ii) full-length *Foc1*Mfs1 structure: two Zn^2+^ ions, one NAD^+^ and one ADP ligand. Modeling outputs were analyzed by quality parameters (pLDDT, EAE, piTM, and pTM) as well as by comparison to our obtained experimental data (Figs. 7, S9, S14, and Table S5).

### Inference of phylogenetic relationships and sequence similarities

Amino acid sequences of MacroD-type macrodomains from all kingdoms of life and viruses (Tables S1 and S2), Zn-Macros (Table S3), and GcvH(-L) (Table S4) proteins were identified by BlastP searches using known class members. Sequences were imported into JalView v2.11.2.7 (102) and aligned using Muscle (103). Sequences were manually screened for quality and sequences with incomplete catalytic domains (based on crystallographic data to determine domain boundaries) rejected. From this sub-set, sequences were selected ensuring appropriate reflection of sequence diversity and macrodomain sequences extracted. Final alignments were generated using the Mafft L-INS-I algorithm (104). Note, the alignment of all MacroD-type macrodomains excludes the 3α-bundle of the Zn-dependent macrodomains, whereas this region is included in the Zn-dependent macrodomain-only alignment. The evolutionary history was inferred by using the Maximum Likelihood method and Le_Gascuel_2008 model (105). Initial tree(s) for the heuristic search were obtained automatically by applying the Maximum Parsimony method. A discrete Gamma distribution was used to model evolutionary rate differences among sites. All positions with less than 95% site coverage were eliminated, that is, fewer than 5% alignment gaps, missing data, and ambiguous bases were allowed at any position (partial deletion option). Confidence levels were estimated using 500 cycles of bootstrap method. Evolutionary analyses were conducted in MEGA11 (106).

Alignment representation were created with JalView v2.11.2.7 (102) and ALINE v1.0.025 (107).

## Data availability

All collected atomic coordinates and structure factors have been deposited in the Protein Data Bank under accession codes 8RSI (*Mor*Macro), 8RSJ (*Mor*Macro:ADPr), 8RSK (*Mor*Macro:Asn-ADPr), 8RSL (*Sau*Macro), 8RSM (*Spy*Macro:ADPr), and 8RSN (*Foc1*Mfs1).

SAXS profiles and pair distribution functions were uploaded to the SASBDB and are available under the accession codes SASDTX8 (lipoyl-*Spy*GcvH-L), SASDTY8 (*Spy*Macro), and SASDTZ8 (*Spy*Macro:lipoyl-*Spy*GcvH-L).

Genomic sequences obtained in this study were deposited in GenBank under the accession numbers OR133610 (*Ate*Mfs1), OR133608 (*Foc1*Mfs1), OR133609 (*Pnp*Mfs1), and OR136504 (*Foc1*MacroD2).

## Supporting information

This article contains supporting information.

## Conflicts of interest

The authors declare that they have no conflicts of interest with the contents of this article.
