# Supplementary material for: Evolutionary and molecular basis of ADP-ribosylation reversal by zinc-dependent macrodomains
Source: J Biol Chem. 2024 Sep 11;300(10):107770. doi: 10.1016/j.jbc.2024.107770 (PMC11490716; doi:10.1016/j.jbc.2024.107770)

# Supporting Information

# Evolutionary and molecular basis of ADP-ribosylation reversal by zinc-dependent macrodomains

Antonio Ariza[1,2,#], Qiang Liu[3,4,5,#], Nathan Cowieson[6], Ivan Ahel[2], Dmitri V. Filippov[3], Johannes Gregor Matthias Rack[7]

[1]School of Biosciences, University of Sheffield, Alfred Denny Building, Western Bank, Sheffield, S10 2TN, UK

[2]Sir William Dunn School of Pathology, University of Oxford, South Parks Road, Oxford, OX1 3RE, UK

[3]Bio-Organic Synthesis, Leiden Institute of Chemistry, Leiden University, Leiden 2300 RA, The Netherlands

[4]Zhongshan Institute for Drug Discovery, Shanghai Institute of Materia Medica, Chinese Academy of Sciences, China.

[5]Shanghai Institute of Materia Medica, Chinese Academy of Sciences, China

[6]Harwell Science and Innovation Campus, Diamond Light Source, Didcot, Oxfordshire, OX11 0DE, UK

[7]Medical Research Council Centre for Medical Mycology at the University of Exeter, University of Exeter, Geoffrey Pope Building, Stocker Road, Exeter, EX4 4QD, UK

[#]These authors contributed equally to this work.

**Running title:** Study on the function of Zn-Macros

**Correspondence to:** Johannes G. M. Rack, Dmitri V. Filippov, and Ivan Ahel

**Content:**

- Experimental procedure for Asn-ADPr chemical synthesis
- Figures S1-S14
- Tables S1-S7
- NMR spectra

# Experimental procedure for Asn-ADPr chemical synthesis

## General procedure

All chemicals were used as received unless stated otherwise. All solvents used in the reactions (including solid phase synthesis) were dried over 3 Å molecular sieves. Solvent removal by rotary evaporation was under reduced pressure at 40 °C. Reactions were monitored by TLC-analysis using Merk 25 DC 60 $F_{254}$ plastic-supported sheets and compound detection by spraying with 20% $H_2SO_4$ in MeOH or $(NH_4)_6Mo_7O_{24}$ • 4 $H_2O$ (25 g/L) and $(NH_4)_4Ce(SO_4)_4$ • 2 $H_2O$ in 10% sulfuric acid, followed by charring at approx. 150 °C. LC-MS analysis was performed on a Thermo Finnigan LCQ Advantage MAX ion-trap mass spectrometer with an electrospray ion source coupled to Surveyor HPLC system (Thermo Finnegan) using an analytical Gemini C18 column (Phenomex, 50 x 4.60 mm, 3 micron) in combination with eluents A: $H_2O$; B: MeCN and C: 1% aq. TFA as the solvent system. High resolution mass spectra were recorded by direct injection (2 µL of a 2 µM solution in water/acetonitrile; 50/50 [v/v] and 0.1% formic acid) on a mass spectrometer (Thermo Finnigan LTQ Orbitrap) equipped with an electrospray ion source in positive mode with resolution R = 60000 at m/z 400 (mass range m/z = 150-2000) and dioctylpthalate (m/z = 391.2842) as a "lock mass". The high-resolution mass spectrometer was calibrated prior to measurements with a calibration mixture (Thermo Finnigan). $^{1}$H-, $^{13}$C- and $^{31}$P-NMR spectra were measured on Brüker DPX-300, Brüker AV-400/500/600/850 and all individual signal was assigned using 2D-NMR spectroscopy. Chemical shifts were given in ppm (δ) relative to TMS (0 ppm) or indirectly referenced to $H_3PO_4$ (0.00 ppm) in $D_2O$ via the solvent residual signal and coupling constants were given in Hz. Infrared (IR) spectra were record on a Shimadzu FT-IR 8300. Optical rotation was measured by MCP 100 Modular Circular Polarimeter using methanol as solvent.

## Building blocks and final products synthesis

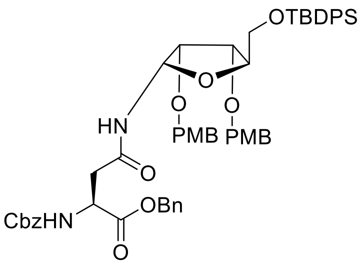


***N*^γ-(2,3-di-*O*-(4-methoxylbenzyl)-5-*O*-*tert*-butyldiphenylsilyl-α-ᴅ-ribosyl)-*N*^α-benzyloxycarbonyl asparagine benzyl ester (4)**

The trifluoroacetimidate donor (**2**) (2.30 g, 2.88 mmol) and Cbz-Asn-OBn (0.85g, 2.40 mmol) were co-evaporated with 1,4-dioxane (3x), dissolved in dry DCM/1,4-dioxane (24 mL, 1/1 [v/v]) and stirred with freshly activated 3 Å molecular sieves at room temperature for 1 h under $N_2$ to remove traces of water. The reaction mixture was cooled to -10 °C and TBSOTf (33 µL, 0.14 mmol) was added to the reaction mixture. The mixture was allowed to reach room temperature and stirred for 30 min. The reaction was quenched by the addition of triethylamine (0.2 mL), filtered through a pad of celite and concentrated. The mixture was purified by silica gel chromatography (pentane/EtOAc, 80/20 – 70/30 – 60/40) to obtain **4** as a white foam (2.0 g, 2.14 mmol, 89%). $^{1}$H NMR (400 MHz, Chloroform-*d*) δ 7.67 – 7.51 (m, 4H, arom.), 7.47 – 7.13 (m, 20H, arom.), 6.96 (d, *J* = 9.2 Hz, 1H, NH), 6.84 (ddd, *J* = 12.1, 6.1, 2.5 Hz, 4H, arom.), 6.05 (d, *J* = 8.8 Hz, 1H, NH), 5.82 (dd, *J* = 9.2, 5.4 Hz, 1H, H1'), 5.25 – 5.00 (m, 4H, $CH_2$ PMB), 4.65 – 4.57 (m, 2H, CH Asn, $CH_2$ Bn), 4.54 – 4.40 (m, 3H, $CH_2$ Bn), 4.19 – 4.00 (m, 3H, H2, H3, H4), 3.84 – 3.71 (m, 6H, OMe PMB), 3.55 (d, *J* = 3.7 Hz, 2H,

H5), 2.96 (AB, $J$ = 16.1, 4.4 Hz, 1H, $CH_2$ Asn), 2.68 (dd, $J$ = 16.2, 4.4 Hz, 1H, $CH_2$ Asn), 0.97 (s, 9H, $CH_3$ TBDPS). $^{13}C$ NMR (101 MHz, $CDCl_3$) δ 170.99, 170.18, 159.61, 159.56 (CO), 135.74, 135.63 (arom.), 133.19, 132.81 (Cq. arom.), 130.31, 129.98 (arom.), 129.86 (Cq. arom.), 129.65, 129.55 (arom.), 129.42 (Cq. arom.), 128.66, 128.64, 128.34, 128.30, 128.19, 128.09, 127.93, 114.10, 114.07 (arom.), 83.20 (C4), 78.73 (C1), 77.69 (C3), 76.67 (C2), 72.42, 72.37 ($CH_2$ Bn), 67.57, 67.08 ($CH_2$ PMB), 64.15 (C5), 55.43 (OMe PMB), 50.85 (CH Asn), 38.15 ($CH_2$ Asn), 26.91 ($CH_3$ TBDPS), 19.29 (Cq. TBDPS). IR (film): 2939, 1723, 1612, 1511, 1507, 1302, 1247, 1212, 1173, 1113, 1030, 822, 701 $cm^{-1}$. HRMS ($ESI^+$) calcd for $C_{56}H_{63}N_2O_{11}Si$ (M+H) 967.4196. Found 967.4201. $[\alpha]_D^{20}$ +25.1 (c = 1, in MeOH)

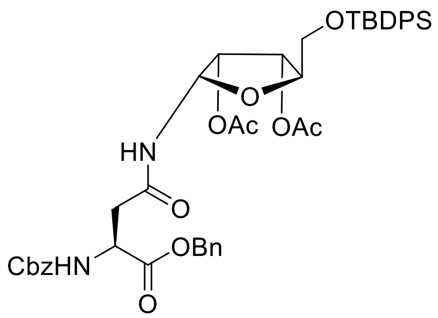


**$N^{\gamma}$-(2,3-di-*O*-acetyl-5-*O*-*tert*-butyldiphenylsilyl-α-ᴅ-ribosyl)-$N^{\alpha}$-benzyloxycarbonyl asparagine benzyl ester (5)**

HCl solution (0.74 mL, 0.1M in HFIP) was added into HFIP (15 mL) then the solution was added into **4** (720 mg, 0.74 mmol). The reaction was stirred for 25 minutes at room temperature and quenched with pyridine (0.2 mL). The mixture was co-evaporated under reduced pressure with toluene (3x) and dissolved in pyridine (7.5 mL). The mixture was cooled to 0 °C and $Ac_2O$ (1.4 mL, 20 eq.) was added, and the reaction was stirred for 16 h at room temperature. The reaction mixture was concentrated *in vacuo*, dissolved in DCM and extracted with aq. $NaHCO_3$ (sat.). The organic layer was dried over $MgSO_4$, concentrated under reduced pressure, and purified by silica gel chromatography (pentane/EtOAc, 100/0 – 80/20 – 70/30 – 60/40) to obtain **5** as a white foam (318 mg, 0.39 mmol, 53%). $^1H$ NMR (400 MHz, Chloroform-*d*) δ 7.67 (ddd, $J$ = 7.9, 3.9, 1.8 Hz, 4H, arom.), 7.46 – 7.35 (m, 6H, arom.), 7.35 – 7.25 (m, 10H, arom.), 6.42 (d, $J$ = 9.5 Hz, 1H, NH), 6.07 (dd, $J$ = 9.5, 5.6 Hz, 1H, H1), 5.99 (d, $J$ = 8.2 Hz, 1H, NH), 5.56 (dd, $J$ = 5.3, 2.7 Hz, 1H, H3'), 5.50 (t, $J$ = 5.5 Hz, 1H, H2'), 5.24 – 5.13 (m, 2H, $CH_2$ Cbz), 5.09 (s, 2H, $CH_2$ Bn), 4.63 (dt, $J$ = 8.6, 4.6 Hz, 1H, CH Asn), 4.09 (dd, $J$ = 5.1, 2.3 Hz, 1H, H4), 3.71 (d, $J$ = 2.9 Hz, 2H, H5), 3.03 (AB, $J$ = 16.0, 4.6 Hz, 1H, $CH_2$ Asn), 2.88 – 2.76 (m, 1H, $CH_2$ Asn), 2.13 – 1.97 (m, 6H, $CH_3$ Ac), 1.06 (s, 9H, $CH_3$ TBDPS). $^{13}C$ NMR (101 MHz, $CDCl_3$) δ 170.74, 169.74, 169.48, 169.17, 156.26 (CO), 136.08 (Cq. arom.), 135.72, 135.66 (arom.), 135.29, 132.83, 132.54 (Cq. arom.), 129.95, 129.91, 128.63, 128.60, 128.43, 128.29, 128.27, 128.07, 127.90 (arom.), 82.34 (C4), 78.66 (C1), 72.56 (C3), 70.11 (C2), 67.69 ($CH_2$ Bn), 67.13 ($CH_2$ Cbz), 63.74 (C5), 50.86 (CH Asn), 38.25 ($CH_2$ Asn), 26.80 ($CH_3$ TBDPS), 20.86, 20.50 ($CH_3$ Ac), 19.20 (Cq. TBDPS).

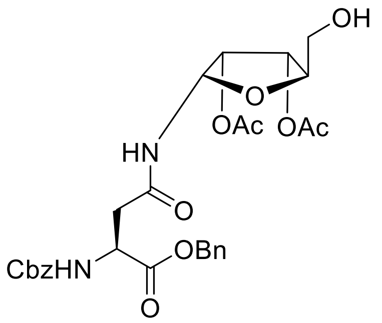


**$N^{\gamma}$-(2,3-di-*O*-acetyl-α-ᴅ-ribosyl)-$N^{\alpha}$-benzyloxycarbonyl asparagine benzyl ester (6)**

Compounds **5** (318 mg, 0.39mmol) were dissolved in pyridine (4 mL) and HF•pyridine (0.15 mL, 5.89 mmol) was added. The reaction was stirred for 2.5 h at 0 °C and TLC showed incomplete reversion. Additional HF•pyridine (0.15 mL, 5.89 mmol) was added and 2 h later the reaction was quenched by aq. $NaHCO_3$ (sat.). The reaction mixture was extracted with EtOAc, the organic layer dried over $MgSO_4$, concentrated under reduced pressure and purified by silica gel chromatography (pentane/acetone, 75/25 – 60/40 – 70/30 – 60/40) to obtain **6** as a white foam (173 mg, 0.30 mmol, 77%). $^1$H NMR (400 MHz, Chloroform-*d*) δ 7.39 – 7.20 (m, 10H, arom.), 6.63 (d, *J* = 9.5 Hz, 1H, NH), 6.09 (d, *J* = 8.2 Hz, 1H, NH), 6.01 (dd, *J* = 9.5, 4.8 Hz, 1H, H1), 5.37 – 5.31 (m, 2H, H2, H3), 5.23 – 4.98 (m, 4H, $CH_2$ Bn, Cbz), 4.60 (dt, *J* = 8.2, 4.9 Hz, 1H, CH Asn), 4.07 (q, *J* = 3.2 Hz, 1H, H4), 3.74 (AB, *J* = 12.4, 2.9 Hz, 1H, H5), 3.59 (AB, *J* = 12.5, 3.4 Hz, 1H, H5), 2.99 (AB, *J* = 16.1, 5.2 Hz, 1H, $CH_2$ Asn), 2.81 (AB, *J* = 16.1, 4.7 Hz, 2H, $CH_2$ Asn), 2.07 (s, 6H, $CH_3$ Ac), 1.25 (s, 1H, OH). $^{13}$C NMR (101 MHz, $CDCl_3$) δ 170.86, 170.36, 169.74, 169.36, 156.32 (CO), 136.14, 135.32 (Cq arom.), 128.65, 128.61, 128.47, 128.29, 128.26, 128.09 (arom.), 81.47 (C4), 78.64 (C1), 71.53 (C3), 70.47 (C2), 67.67 ($CH_2$ Bn), 67.13 ($CH_2$ Cbz), 61.89 (C5), 50.84 (CH Asn), 38.12 ($CH_2$ Asn), 20.75, 20.55 ($CH_3$ Ac). IR (film): 3364, 1734, 1715, 1507, 1374, 1238, 1214, 1194, 1179, 1043, 1028 cm$^{-1}$. HRMS (ESI$^+$) calcd for $C_{28}H_{32}N_2O_{11}$ (M+H) 573.2079. Found 573.2077. $[\alpha]_D^{20}$ +47.1 (c = 1, in $CHCl_3$)

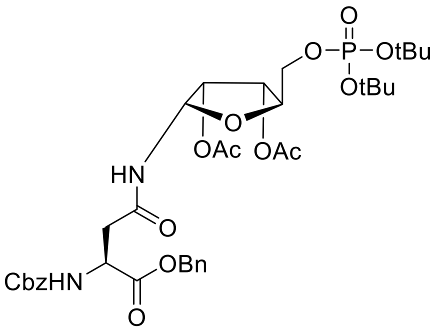


**$N^{\gamma}$-(2,3-di-*O*-acetyl-5-*O*-(di-*tert*-butyl)-phosphoryl-α-ᴅ-ribosyl)-$N^{\alpha}$-benzyloxycarbonyl asparagine benzyl ester (7)**

Firstly, co-evaporating 1-methylimidazolium chloride (162 mg, 1.36 mmol) and 1-methyl-imidazole (72 µL, 0.91 mmol) with dry $CH_3CN$ (3 x), then $N_2$ was applied. To the mixture, freshly activated molecular sieves and dry DMF (2.3 mL) were added and the activator solution was stirred at room temperature for 1 h under $N_2$. Secondly, co-evaporating **6** (130 mg, 0.23 mmol) with dry 1,4-dioxane (3x), then activator solution was added after which Di-tert-butyl-N,N-diisopropylphosphoramidite (0.11 mL, 0.34 mmol) was added and the reaction was stirred at room temperature for 30 min. Then *t*BuOOH in decane (0.41 mL, 5.5 M, 2.27 mmol) was added at 0 ℃ and the reaction mixture was stirred for 1 h at room temperature. The reaction was quenched upon addition of aq. $NaHCO_3$ (sat.) and extracted with EtOAc (3x), dried over $MgSO_4$, concentrated under reduced pressure. Purification by silica gel chromatography (pentane/acetone, 100/0 – 80/20 – 75/25) to obtain **7** as a colourless oil (153 mg, 0.20 mmol, 87%). $^1$H NMR (400 MHz, Chloroform-*d*) δ 7.48 – 7.16 (m, 10H, arom.), 6.43 (d, *J* = 9.4 Hz, 1H, NH), 6.02 – 5.96 (m, 2H, H1, NH), 5.39 (dd, *J* = 5.3, 3.5 Hz, 1H, H3), 5.30 (t, *J* = 5.4 Hz, 1H, H2), 5.25 – 5.04 (m, 4H, $CH_2$ Cbz, Bn), 4.61 (dt, *J* = 8.7, 4.6 Hz, 1H, CH Asn), 4.23 – 4.13 (m, 1H, H4), 4.04 (dd, *J* = 5.6, 3.3 Hz, 2H, H5), 3.01 (AB, *J* = 16.1, 4.6 Hz, 1H, $CH_2$ Asn), 2.81 (dd, *J* = 16.1, 4.6 Hz, 1H, $CH_2$ Asn), 2.10 (s, 3H,

$CH_3$ Ac), 2.06 (s, 3H, $CH_3$ Ac), 1.48 (s, 18H, $CH_3$ *t*Bu). $^{13}C$ NMR (101 MHz, $CDCl_3$) δ 170.65, 169.70, 169.34, 168.99, 156.20 (CO), 136.04, 135.24 (Cq. arom.), 128.60, 128.55, 128.41, 128.24, 128.21, 128.02 (arom.), 83.00, 82.98, 82.93, 82.91 (Cq. *t*Bu), 80.06, 79.98 (C4), 78.52 (C1), 71.89 (C3), 69.73 (C2), 67.63 ($CH_2$ Cbz), 67.08 ($CH_2$ Bn), 65.75, 65.69 (C5), 50.76 (CH Asn), 38.15 ($CH_2$ Asn), 29.84, 29.80 ($CH_3$ *t*Bu), 20.71, 20.42 ($CH_3$ Ac). $^{31}P$ NMR (162 MHz, $CDCl_3$) δ -9.93. HRMS (ESI$^+$) calcd for $C_{36}H_{49}N_2O_{14}PNa$ (M+Na) 787.2814. Found 787.2809.

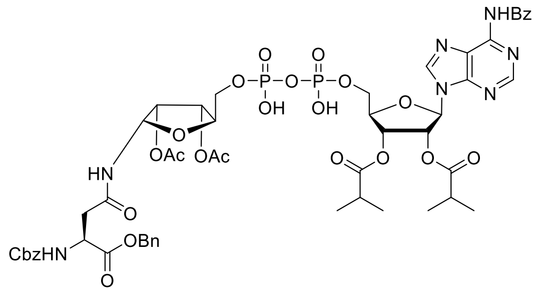

**$N^γ$-($N^6$-benzoyl-2'',3''-di-*O*-acetyl-2'3'-di-O-isobutyryl-adenosine diphosphate-α-ᴅ-ribosyl)-$N^α$-benzyloxycarbonyl asparagine benzyl ester (9)**

1 mL HCl solution (0.1 M in HFIP) was added into HFIP (1 mL) then the solution was added into **7** (70 mg, 0.09 mmol). The reaction was stirred for 30 min at room temperature and quenched with TEA (0.13 mL). The mixture was concentrated under reduced pressure and followed by co-evaporation with toluene (2x), pyridine/$H_2O$ (9/1 [v/v]) (1x), pyridine (1x) and toluene (5x). ETT (2.19 mL, 0.25 M in solution, 0.55 mmol) in $CH_3CN$ and molecular sieves were added, and the mixture was left to stand for 16 h under $N_2$ atmosphere. Compound **8** was co-evaporated with dioxane (1x), $CH_3CN$ (3x), dissolved in $CH_3CN$ (1 mL) and added to the mixture of **7** and ETT. The reaction was stirred for 10 min and analysed by $^{31}P$-NMR spectroscopy. CSO (2 mL, 0.5 M, 1.0 mmol) in $CH_3CN$ was added and after stirring for 20 min analysed by $^{31}P$-NMR. Dry DBU (2 mL, 0.5 M, 1.0 mmol) in DMF was added and after stirring for 5 min pyridinium chloride (116 mg, 1.0 mmol) was added to quench the reaction. The mixture was filtrated and washed by MeOH and then concentrated. Purification by silica gel chromatography (DCM/MeOH, 100/0 – 95/5 –90/0 – 85/15) and then LH-20 (DCM/MeOH, 50/50) to get to obtain **9** as a colourless oil (27 mg, 22 µmol, 24%). $^{31}P$ NMR (162 MHz, $CDCl_3$) δ br. -12.77. LC-MS: Rt = 7.27 min. 10-90% TFA. ESI MS+ calc. 1226.3 found 1226.1 [M+1]$^+$.

**$N^γ$-(adenosine diphosphate-α-ᴅ-ribosyl)-asparagine (1)**

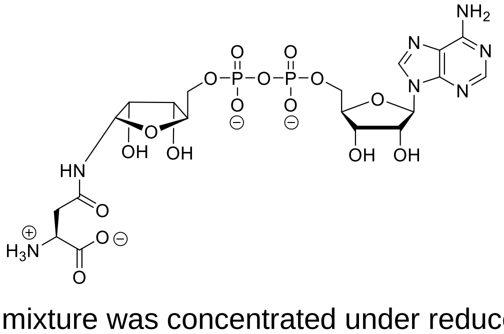

Compound **9** (20 mg, 16 µmol) was added into a flask and then *t*BuOH/Dioxane/$H_2O$ (2 mL, 4:4:1 [v/v]), Pd/C (25 mg, 10% loading) and few drops of AcOH were added. The mixture was sonicated for 5 min under $N_2$. $H_2$ was bubbled through for 48 h and LC-MS showed completely conversion. The reaction was filtrated over celite and washed by MeOH. The mixture was concentrated under reduced pressure and then co-evaporated with dioxane (2x) then dissolved in aq. $NH_4OH$ (2 mL). The reaction was stirred for 24 h after which concentrated under reduced pressure. Purification by HW-40 ($NH_4OAc$ in $H_2O$, 0.15 M), ion exchange (10 mmol – 0.5 mmol $NH_4OAc$ in $H_2O$) and repeat lyophilization obtained **1** as a white solid (0.6 mg, 0.89 µmol, 6%). $^1H$ NMR (400 MHz, Deuterium Oxide) δ 8.47 (d, $J$ = 2.8 Hz, 1H, H2), 8.22 (s, 1H, H8), 6.11 (d, $J$ = 5.9 Hz, 1H, H1'), 5.70 – 5.59 (m, 1H, H1''), 4.74 (t, $J$ = 5.6 Hz, 1H, H2'), 4.50 (dd, $J$ = 5.2, 3.5 Hz, 1H, H3'), 4.36 (t, $J$ = 2.8 Hz, 1H, H4'), 4.19 (td, $J$ = 4.6, 4.1, 2.3

Hz, 3H, H2'', H5'), 4.11 – 3.89 (m, 4H, H3'', H4'', H5''), 3.02 (AB, $J$ = 17.2, 3.9 Hz, 1H, $CH_2$ Asn), 2.81 (AB, $J$ = 17.2, 9.4 Hz, 1H, $CH_2$ Asn). $^{13}C$ NMR (101 MHz, $D_2O$) δ 86.74 (C1'), 83.91, 83.83 (C4'), 80.63 (C4''), 80.19 (C1''), 74.18 (C2'), 70.59 (C3'), 70.33 (C2''), 69.94 (C3''), 65.32 (C5'), 65.17 (C5''), 51.04 (CH Asn), 35.42 ($CH_2$ Asn). $^{31}P$ NMR (202 MHz, $D_2O$) δ -10.35, -10.45, -10.60, -10.71. HRMS ($ESI^+$) calcd for $C_{19}H_{30}N_7O_{16}P_2$ (M+H) 674.1219. Found 674.1238. LC-MS: Rt = 2.80 min. 0 - 50% $NH_4OAc$. ESI MS+ calc. 674.1 found 674.1 [M+1]

## Supplemental Figures

**Figure S1. Multiple sequence alignment of MacroD-type macrodomains.** The core macrodomain sequences, excluding e.g. 3α-bundle of Zn-Macros, were extracted from the full-length protein context and aligned using the Mafft L-INS-I algorithm. The NAAN motif (‡), proposed catalytic residues (§) and active site arene (Ω) are indicated above the alignment, whereas their occurrence within the sequences is indicated by coloured boxes (blue, green, and yellow, respectively). The zinc-coordinating residues of Zn-Macros are indicated by red boxes. Note, while viral nsp3 MOD1-like macrodomains cluster together phylogenetically, viral family specific catalytic residues were proposed.

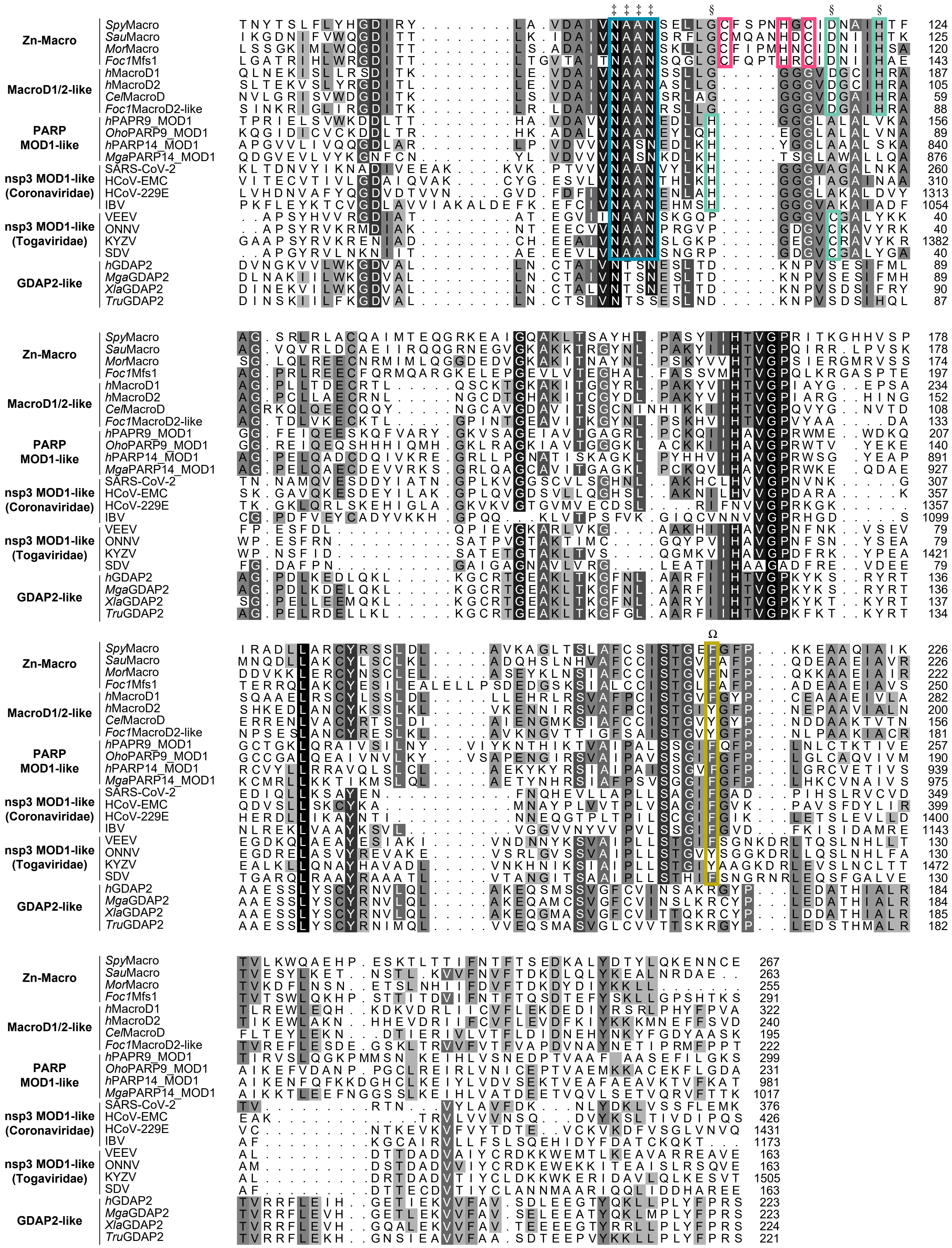

Zn-Macro
SpyMacro
SauMacro
MorMacro
Foc1Mfs1
MacroD1/2-like
hMacroD1
hMacroD2
CelMacroD
Foc1MacroD2-like
PARP MOD1-like
hPAPR9_MOD1
OhoPARP9_MOD1
hPARP14_MOD1
MgaPARP14_MOD1
nsp3 MOD1-like (Coronaviridae)
SARS-CoV-2
HCoV-EMC
HCoV-229E
IBV
nsp3 MOD1-like (Togaviridae)
VEEV
ONNV
KYZV
SDV
GDAP2-like
hGDAP2
MgaGDAP2
XlaGDAP2
TruGDAP2
‡ ‡ ‡ ‡
§
Ω
124 125 120 143 187 105 59 88 156 89 840 876 260 310 1313 1054 40 40 1382 40 89 89 90 87
178 178 174 197 234 152 108 133 207 140 891 927 307 357 1357 1099 79 79 1421 79 136 136 137 134
226 226 222 250 282 200 156 181 257 190 939 975 349 399 1400 1143 130 130 1472 130 184 184 185 182
267 263 255 291 322 240 195 222 299 231 981 1017 376 426 1431 1173 163 163 1505 163 223 223 224 221

**Figure S2. Structural comparison of Zn-Macros.** (a) Ribbon-liquorice representation of structural alignment of all here determined Zn-Macros in comparison to the earlier solved *Sau*Macro apo (PDB 5KIV). The SirTM domain of *Foc1*Mfs1 is shown in white. (b) Ribbon-liquorice representation of ligand coordination by *Spy*Macro and *Mor*Macro coloured as in (a). Selected residues important for ligand coordination and catalysis are given for *Spy*Macro and are conserved in *Mor*Macro (see also Fig. S1). (c) Electron density omit maps (2Fo-Fc contoured at 1 σ) refined in the absence of any ligand. The final refined protein–ligand structures are shown as reference. (d) Ribbon-liquorice representation of structural alignment of Zn-Macros with human MacroD1. The conformational change of loop 2 upon ligand binding is illustrated in the close-up (right panel).

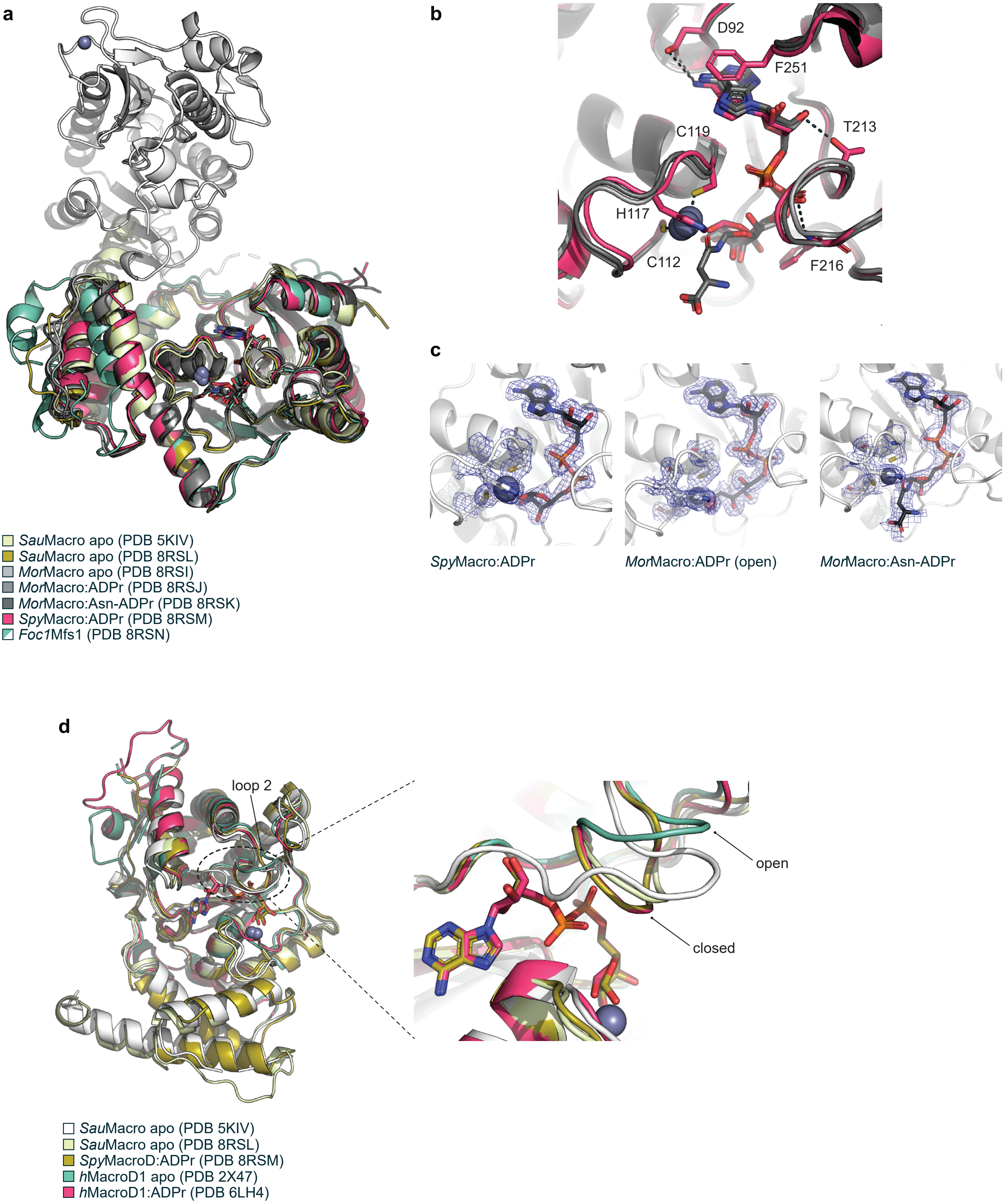

a
b
D92
F251
T213
C119
H117
C112
F216
c
SpyMacro:ADPr
MorMacro:ADPr (open)
MorMacro:Asn-ADPr
SauMacro apo (PDB 5KIV)
SauMacro apo (PDB 8RSL)
MorMacro apo (PDB 8RSI)
MorMacro:ADPr (PDB 8RSJ)
MorMacro:Asn-ADPr (PDB 8RSK)
SpyMacro:ADPr (PDB 8RSM)
Foc1Mfs1 (PDB 8RSN)
d
loop 2
open
closed
SauMacro apo (PDB 5KIV)
SauMacro apo (PDB 8RSL)
SpyMacroD:ADPr (PDB 8RSM)
hMacroD1 apo (PDB 2X47)
hMacroD1:ADPr (PDB 6LH4)

**Figure S3. Structural insights into active site zinc-coordination in absence of ligand.** Ribbon-liquorice representation of different modes of zinc-coordination in the apo structures of *Sau*Macro and *Mor*Macro. Promoters deriving from symmetry molecules are highlighted in green (indicates as $A_{sym}$) and $Zn^{2+}$ ion as grey sphere. Coordination bonds are given as black dashed lines.

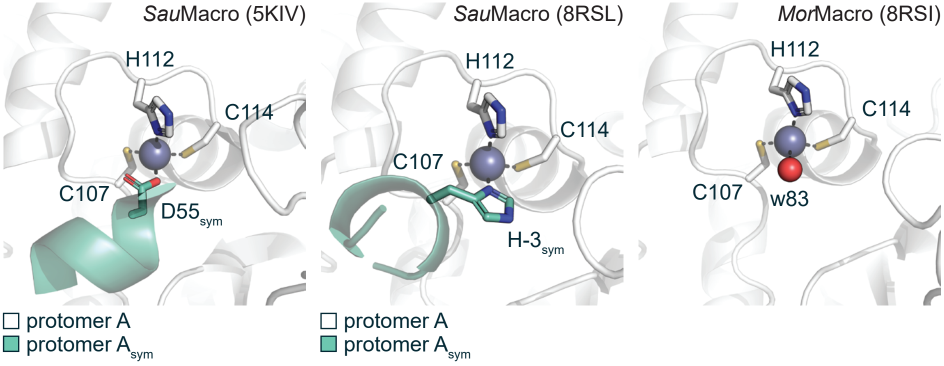
SauMacro (5KIV)
H112
C114
C107
D55sym
protomer A
protomer Asym
SauMacro (8RSL)
H112
C114
C107
H-3sym
protomer A
protomer Asym
MorMacro (8RSI)
H112
C114
C107
w83

**Figure S4. Zinc coordination by *Foc1*Mfs1.** Electron density omit maps (2Fo-Fc contoured at 1 σ) refined in the absence of any ligand. The final refined protein–ligand structures are shown as reference. (a) The Zn-loop has no associated electron density and therefore could not be modelled. The AlphaFold 3-derived Zn-loop and $Zn^{2+}$ ion are shown for reference. (b) The structural $Zn^{2+}$ ion of the small subdomain of the SirTM domain in *Foc1*Mfs1 is associated with well-defined electron density.

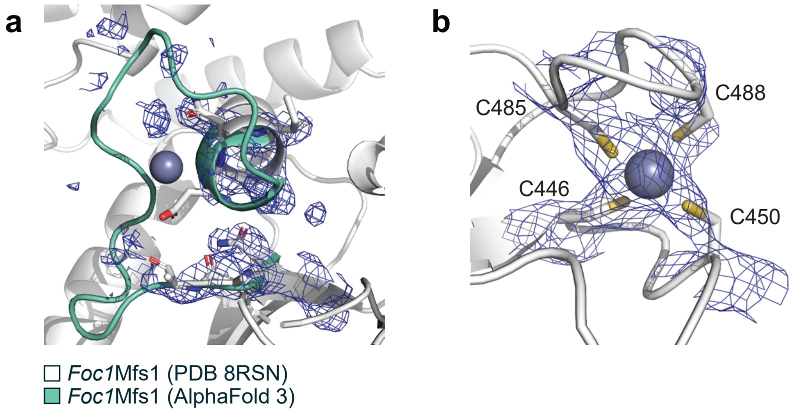
a
b
C485
C488
C446
C450
Foc1Mfs1 (PDB 8RSN)
Foc1Mfs1 (AlphaFold 3)

**Figure S5. Interaction between 3α-bundle and macrodomain in Zn-Macros.** (a) Ribbon-liquorice representation of polar contact (black dashed lines) between the 3α-bundle and macrodomain in *Sau*Macro. (b) Multiple sequence alignment of the 3α-bundle:macrodomain interaction region of representative Zn-Macros. Conserved residues participating in the interactions are indicated by down-pointing triangle (▼), non-conserved residues participating in interaction in *Sau*Macro by empty circles (○), the NAAN motif by double daggers (‡), zinc-coordinating residues by koppa (ϟ), and the catalytic DH dyad by section sign (§). Sequences used in this study are highlighted in red. and Further sequence information are given in Tab. S3.

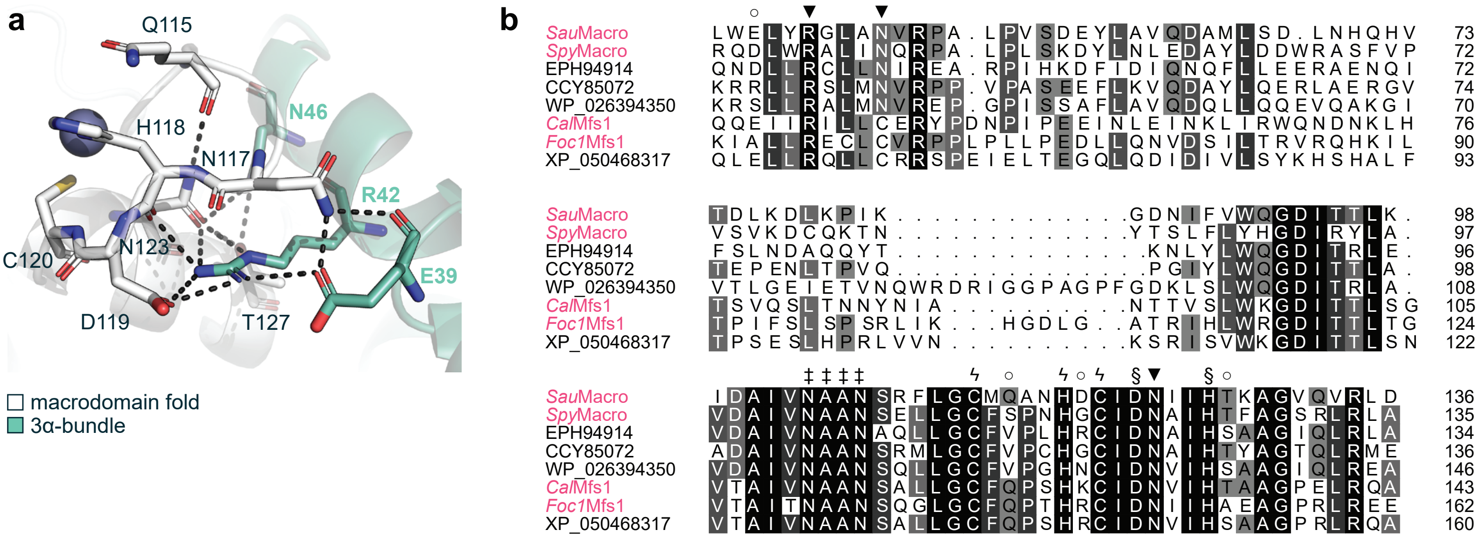
a
Q115
H118
N46
N117
R42
C120
N123
E39
D119
T127
macrodomain fold
3α-bundle
b
○ ▼ ▼
SauMacro LWELYRGLANVRPA.LPVSDEYLAVQDAMLSD.LNHQHV 73
SpyMacro RQDLWRALINQRPA.LPLSKDYLNLEDAYLDDWRASFVP 72
EPH94914 QNDLLRCLLNIREA.RPIHKDFIDIQNQFLLEERAENQI 72
CCY85072 KRRLLRSLMNVRPP.VPASEEFLKVQDAYLQERLAERGV 74
WP_026394350 KRSLLRALMNVREP.GPISSAFLAVQDQLLQQEVQAKGI 70
CalMfs1 QQEIIRILLCERYPDNPIPEEINLEINKLIRWQNDNKLH 76
Foc1Mfs1 KIALLRECLCVRPPLPLLPEDLLQNVDSILTRVRQHKIL 90
XP_050468317 QLELLRQLLCRRSPEIELTEGQLQDIDIVLSYKHSHALF 93
SauMacro TDLKDLKPIK.............GDNIFVWQGDITTLK. 98
SpyMacro VSVKDCQKTN.............YTSLFLYHGDIRYLA. 97
EPH94914 FSLNDAQQYT.............KNLYLWQGDITRLE. 96
CCY85072 TEPENLTPVQ.............PGIYLWQGDITTLA. 98
WP_026394350 VTLGEIETVNQWRDRIGGPAGPFGDKLSLWQGDITRLA. 108
CalMfs1 TSVQSLTNNYNIA.........NTTVSLWKGDITTLSG 105
Foc1Mfs1 TPIFSLSPSRLIK...HGDLG..ATRIHLWRGDITTLTG 124
XP_050468317 TPSESLHPRLVVN..........KSRISVWKGDITTLSN 122
‡ ‡ ‡ ‡ ϟ ○ ϟ ○ ϟ § ▼ § ○
SauMacro IDAIVNAANSRFLGCMQANHDCIDNIIHTKAGVQVRLD 136
SpyMacro VDAIVNAANSELLGCFSPNHGCIDNAIHTFAGSRLRLA 135
EPH94914 VDAIVNAANAQLLGCFVPLHRCIDNAIHSAAGIQLRLA 134
CCY85072 ADAIVNAANSRMLGCFVPCHGCIDNAIHTYAGTQLRME 136
WP_026394350 VDAIVNAANSQLLGCFVPGHNCIDNVIHSAAGIQLREA 146
CalMfs1 VTAIVNAANSALLGCFQPSHKCIDNVIHTAAGPELRQA 143
Foc1Mfs1 VTAITNAANSQGLGCFQPTHRCIDNIIHAEAGPRLREE 162
XP_050468317 VTAIVNAANSALLGCFQPSHRCIDNVIHSAAGPRLRQA 160

**Figure S6. ADPr coordination by Zn-Macros.** (a) Ribbon-liquorice representation of the distal ribose coordination in its closed form within the *Spy*Macro crystal structure. ADPr is shown in yellow and polar contacts are indicated by dashed black lines. (b) 2D ligand interaction diagram showing the coordination of ADPr within the active site of *Spy*Macro. Polar and π-π are indicated by red and turquoise dashes lines, respectively, and hydrophobic by yellow lines. The diagram was generated using PoseEdit (https://proteins.plus). (c) Ribbon-liquorice representation of the distal ribose coordination in its open form within the *Mor*Macro crystal structure. Promtomer A is shown in white, residues from the symmetry related protomer B ($B_{sym}$) are shown in turquoise, ADPr in yellow and polar contacts as black dashed lines. (d) 2D ligand interaction diagram showing the coordination of ADPr within the active site of *Mor*Macro. Colouring scheme is as indicated in (b).

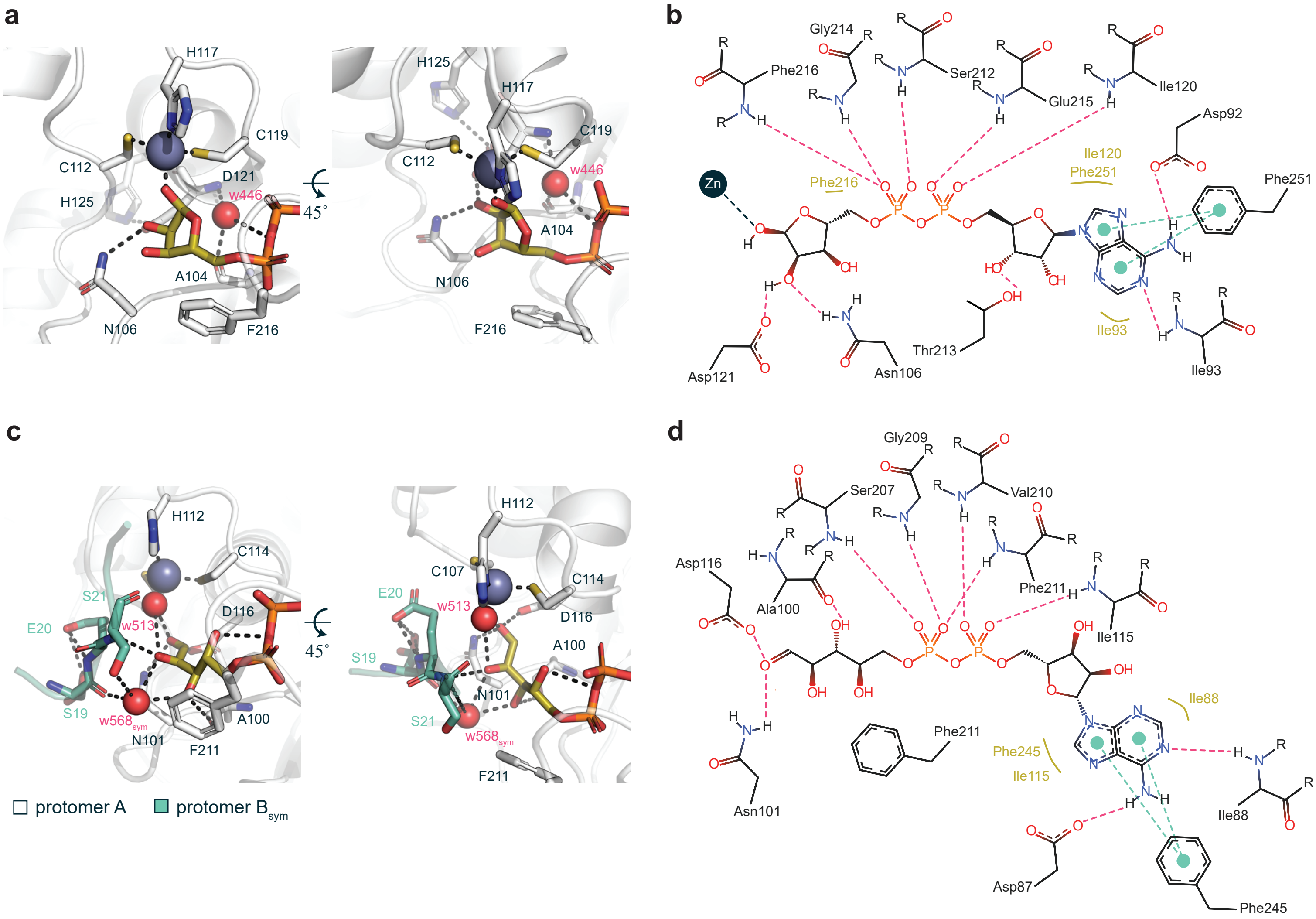
a
H117
C112
C119
D121
w446
H125
45°
A104
N106
F216
H125
H117
C119
C112
w446
A104
N106
F216
b
Gly214
Phe216
Ser212
Glu215
Ile120
Asp92
Zn
Phe216
Ile120
Phe251
Phe251
Asp121
Asn106
Thr213
Ile93
Ile93
c
H112
C114
S21
w513
D116
E20
45°
S19
w568sym
N101
A100
F211
H112
C107
C114
E20
w513
D116
A100
S19
N101
S21
w568sym
F211
protomer A
protomer Bsym
d
Gly209
Ser207
Val210
Asp116
Ala100
Phe211
Ile115
Phe211
Ile88
Phe245
Ile115
Asn101
Ile88
Asp87
Phe245

**Figure S7. Analysis of *Sau*Macro:*Sau*GcvH-L interaction by size exclusion chromatography.** Proteins were separated on a Superdex S200 increase column either alone or as 1:1.2 molar ration mixture (*Sau*Macro:*Sau*GcvH-L). The $UV_{280}$ elution profiles are given in the upper panel. The elations were fractionated and analysed by SDS-PAGE and immunoblot. Representative results for the complex are given in the lower panel.

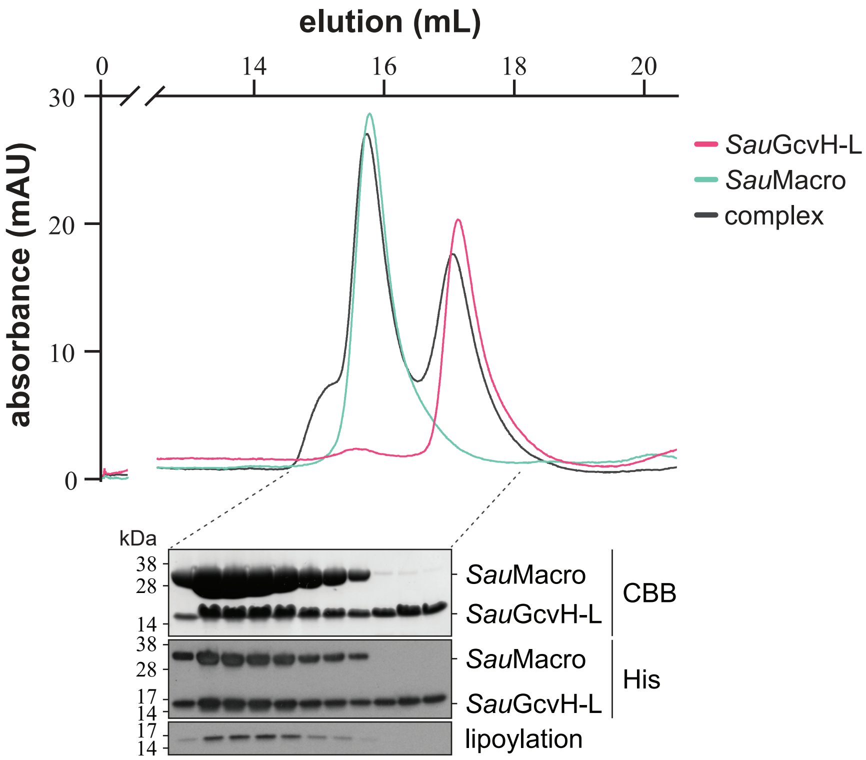

elution (mL)
0
14
16
18
20
30
20
10
0
absorbance (mAU)
SauGcvH-L
SauMacro
complex
kDa
38
28
14
38
28
17
14
17
14
SauMacro
SauGcvH-L
CBB
SauMacro
SauGcvH-L
His
lipoylation

**Figure S8. Comparison of GcvH-L and GcvH proteins.** (a) Visualisation of the electrostatic surface potential of *Sau*Macro, *Spy*Macro, *Mor*Macro, and *Foc1*Mfs1 (AlphaFold 3 model). Charge distribution was calculated using APBS as implemented in PyMOL. Approximate position of the lipoyl acceptor residue (Lys56 in *Spy*GcvH-L) is indicated by circle. Shown canonical GcvH proteins are from *Mycobacterium tuberculosis* (*Mtu*GcvH), *Pisum sativum* (garden pea; *Psa*GcvH), and *Bos taurus* (cattle; *Bta*GcvH) (b) Ribbon representation of *Spy*Macro:*Spy*GcvH-L complex showing orientation of GcvH(-L) proteins in (a) and (c). (c) Ribbon-liquorice representation of structural alignment of GcvH(-L) proteins. The ‘GcvH helix’ is absent in GcvH-L proteins. Lipoyl acceptor lysine is given for all structures and ADP-ribosyl acceptor Asp27 for *Spy*GcvH-L. (d) Multiple sequence alignment of GcvH(-L) proteins. The lipoyl (#) and ADP-ribosyl (⥽) acceptor sites are indicated above the alignment, conserved residues involved in the Zn-Macro:GcvH-L interaction as yellow boxes, and secondary structure elements for *Spy*GcvH-L (green) and *Bta*GcvH (red) below the alignment.

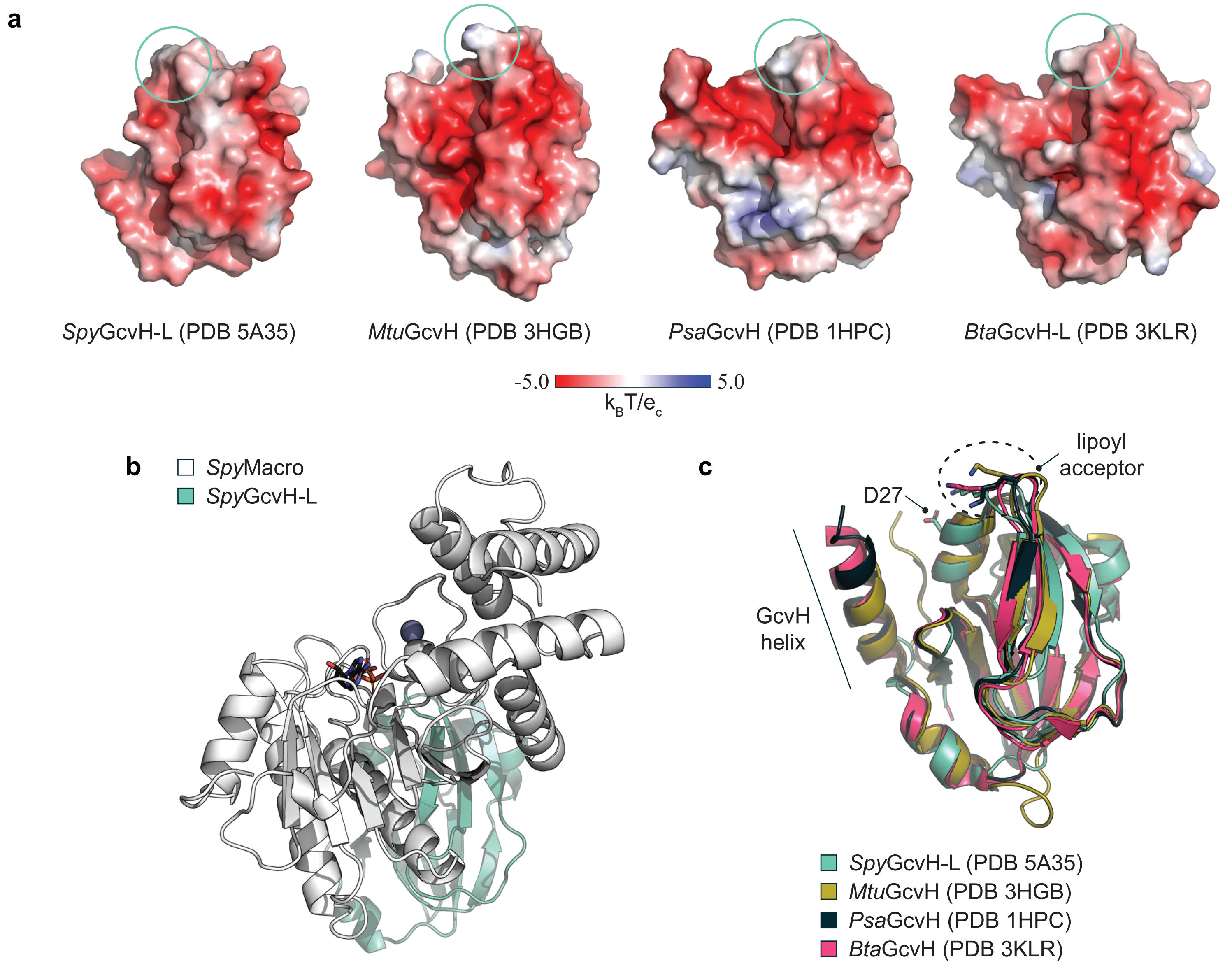


**d**

```
SauGcvH-L  ........MKKLANYLWVEKVG.DLYVFSMTPELQDDIGTVGYVEFVS.PDEVKV   45
SpyGcvH-L  ........MKKIANYLLIEKTD.DRYTISMTPELQDDIGTIGYAEFTD.NDHLAV   45
DpiGcvH-L  ........MRKIGNYLFVEKQD.DIYTIRMTPELQDDVGTVAFVEYNF.EDTLKK   45
LhiGcvH-L  ........MKKRGDYLFVDENN.GVYTISLTPELQDDIGTVGYVEFKN.EKAVSK   45
AurGcvH    .....MEKVYTDNGLWLTQTADKQVIIGLSKAGQEEFGDISFINYLV.DQDLVA   48
DpiGcvH    .....MTVKYHVNGLWIEKKD.DQIIIGLSEKGQDDAGDIMFANLMIDSDYLEA   48
GelGcvH    ......MIKYSENGLWIKQEE.NRYTVGISPKGQDDIGDVSFVELLI.QETLTT   46
SauGcvH    .MAVPNELKYSKEHEWVKVEG.NVATIGITEYAQSELGDIVFVELPETDDEINE   52
MtuGcvH    MSDIPSDLHYTAEHEWIRRSGDDTVRVGITDYAQSALGDVVFVQLPVIGTAVTA   54
PsaGcvH    .SNVLDGLKYAPSHEWVKHEG.SVATIGITDHAQDHLGEVVFVELPEPGVSVTK   86
BtaGcvH    .....SVRKFTEKHEWVTTEN.GVGTVGISNFAQEALGDVVYCSLPEVGTKLNK   96

                      #
SauGcvH-L  DDEIVSIEASKTVIDVQTPLSGTIIERNTKAEEEPTILNSEKPEENWLFKLDDV   99
SpyGcvH-L  DDIILNLEASKTVMSVLSPLAGAVVERNEAATLTPTLLNSEKAEENWIVVLTDV   99
DpiGcvH-L  GDFILFLEASKTVIELKTPISGTVHAKNEAAVDQPNLLNSADEAENWLIQLTDV   99
LhiGcvH-L  GDALAKLEASKTVLDVPSPLAGKVVEKNTKLQDDPKLLNSPDDSQNWLVKLKDV   99
AurGcvH    GEAFVSIEAEKAVTDILAPLSGKVVGKNEKAEGTPTDLNSDKDNLNWLVAVEPS  102
DpiGcvH    GDTLIGVEGAKAVTDFTTPFAGNVLAIHEAIEEDFDLLNSTDRADNWIVKLRPK  102
GelGcvH    EDSFMSVEAAKAVTEFVSPLSGKVVAWHTALEDNPEMLNQVDTTVNWIYTLEEV  100
SauGcvH    GDTFGSVESVKTVSELYAPISGKVVEVNEELEDSPEFVNESPYEKAWMVKVEIS  106
MtuGcvH    GETFGEVESTKSVSDLYAPISGKVSEVNSDLDGTPQLVNSDPYGAGWLLDIQVD  108
PsaGcvH    GKGFGAVESVKATSDVNSPISGEVIEVNTGLTGKPGLINSSPYEDGWMIKIKPT  140
BtaGcvH    QEEFGALESVKAASELYSPLSGEVTEINKALAENPGLVNKSCYEDGWLIKMTFS  150

SauGcvH-L  ...DKE.........AFLALPEA...................  110
SpyGcvH-L  ...DQA.........AFDALEDA...................  110
DpiGcvH-L  ...DEA.........EFAALEDE...................  110
LhiGcvH-L  ...DES.........AFNQLKGA...................  110
AurGcvH    SDFDPT.........AFKSEETK...................  116
DpiGcvH    ...DTS.........LFSQLLDKPFP.....SEDSVG.  122
GelGcvH    ...DEA.........TFASLLDEDLPI.VCEKKEGCGN  125
SauGcvH    ...DES.........QLEALLTAEKYSEMIGE.......  126
MtuGcvH    ...SSDVAALESALTTLLDAEAYRGTLTE......  134
PsaGcvH    ...SPD.........ELESLLGAKEYTKFCEEEDAAH.  165
BtaGcvH    ...NPS.........ELDELMSEEAYEKYIKSIEE...  173
```

GcvH helix

**Figure S9. Structural modelling of non-physiological Zn-Macro:GcvH(-L) complexes**. The complexes of *Dpi*Macro:*Dpi*GcvH, *Sau*Macro:*Sau*GcvH, *Efa*Macro:*Spy*GcvH-L, and *Tpe*Macro:*Spy*GcvH-L were modelled using AlphaFold 3 and include one $Zn^{2+}$ ion and one ADP ligand per model. Model quality indicators ipTM and pTM are given in Tab. S5. (a) Per-residue confidence estimate (pLDDT) mapped onto the predicted structures. The modelled ligands are given as: zinc (grey sphere) and ADP (black sticks), respectively. (b) Expected position error (EPE) of the predicted complexes.

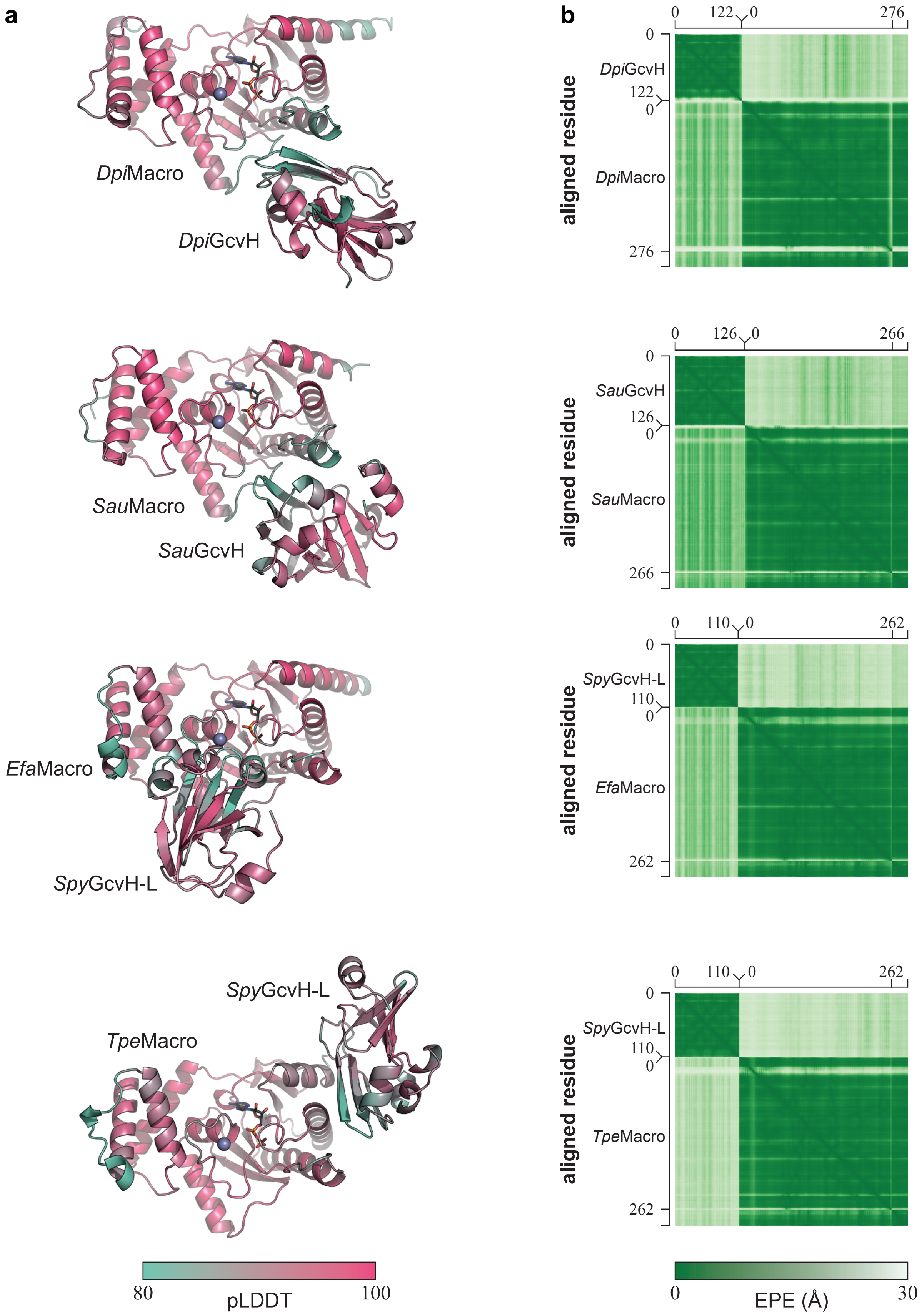
a
b
DpiMacro
DpiGcvH
SauMacro
SauGcvH
EfaMacro
SpyGcvH-L
TpeMacro
SpyGcvH-L
80
pLDDT
100
aligned residue
0
122
0
276
DpiGcvH
DpiMacro
0
126
0
266
SauGcvH
SauMacro
0
110
0
262
SpyGcvH-L
EfaMacro
TpeMacro
EPE (Å)
30

**Figure S10. AlphaFold 3 model comparisons of predicted Zn-Macro:GcvH(-L) complexes.** The five models of each AlphaFold 3 run were structurally aligned to the macrodomain of model 1 in PyMOL and show high model-to-model reproducibility (RMSD values ≤0.13). Placement of GcvH(-L) proteins relative to model 1 Zn-Macro are shown and modelled Zn2+ ion and ADP are given as reference.

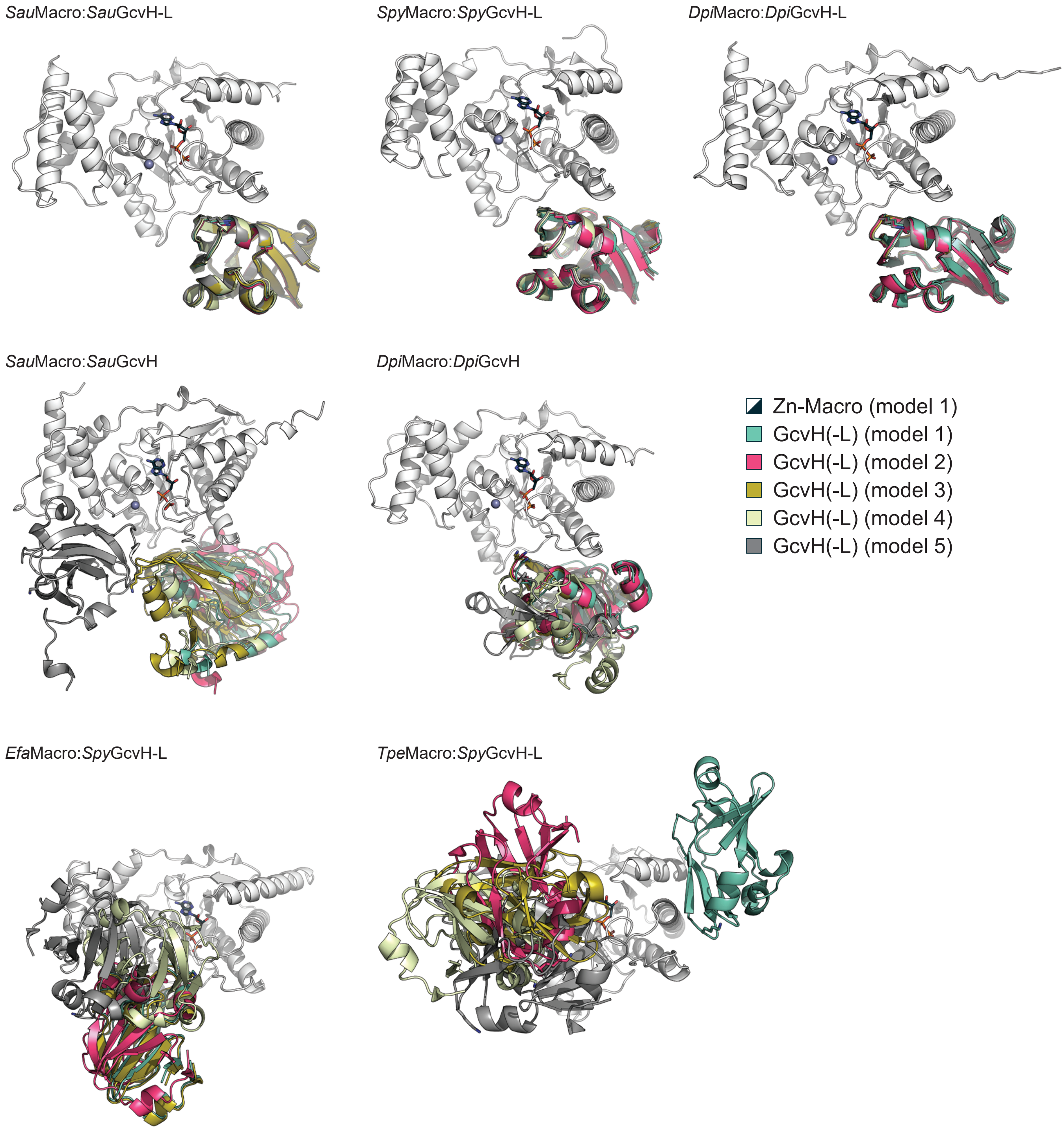
SauMacro:SauGcvH-L
SpyMacro:SpyGcvH-L
DpiMacro:DpiGcvH-L
SauMacro:SauGcvH
DpiMacro:DpiGcvH
Zn-Macro (model 1)
GcvH(-L) (model 1)
GcvH(-L) (model 2)
GcvH(-L) (model 3)
GcvH(-L) (model 4)
GcvH(-L) (model 5)
EfaMacro:SpyGcvH-L
TpeMacro:SpyGcvH-L

**Figure S11. Representative anion exchange chromatography for the enrichment of lipoyl-*Spy*GcvH-L.** In vivo lipoylated and affinity purified *Spy*GcvH-L was loaded onto a HiTrap Q anion exchange column (Cytiva) and eluted using a salt gradient (upper panel). Eluted fractions were analysed by immunoblot for the lipoyl modification.

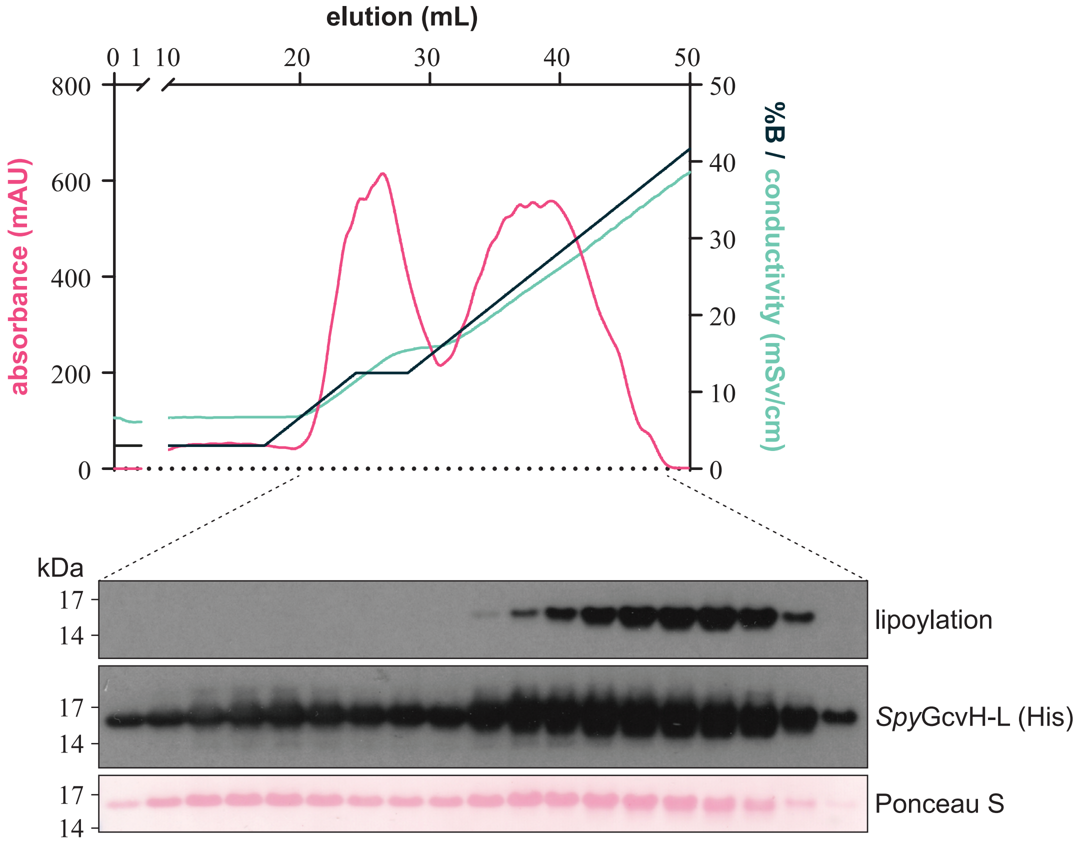

elution (mL)
0
1
10
20
30
40
50
800
600
400
200
0
absorbance (mAU)
50
40
30
20
10
0
%B / conductivity (mSv/cm)
kDa
17
14
lipoylation
17
14
SpyGcvH-L (His)
17
14
Ponceau S

**Figure S12. SAXS data of lipoyl-*Spy*GcvH-L and *Spy*Macro.** Experimental scatter overlaid with hypothetical scattering data generated from solved structures (PDB 5A35, SpyGcvH-L; PDB 8RSM, *Spy*Macro; upper panel) and residual analysis (lower panel).

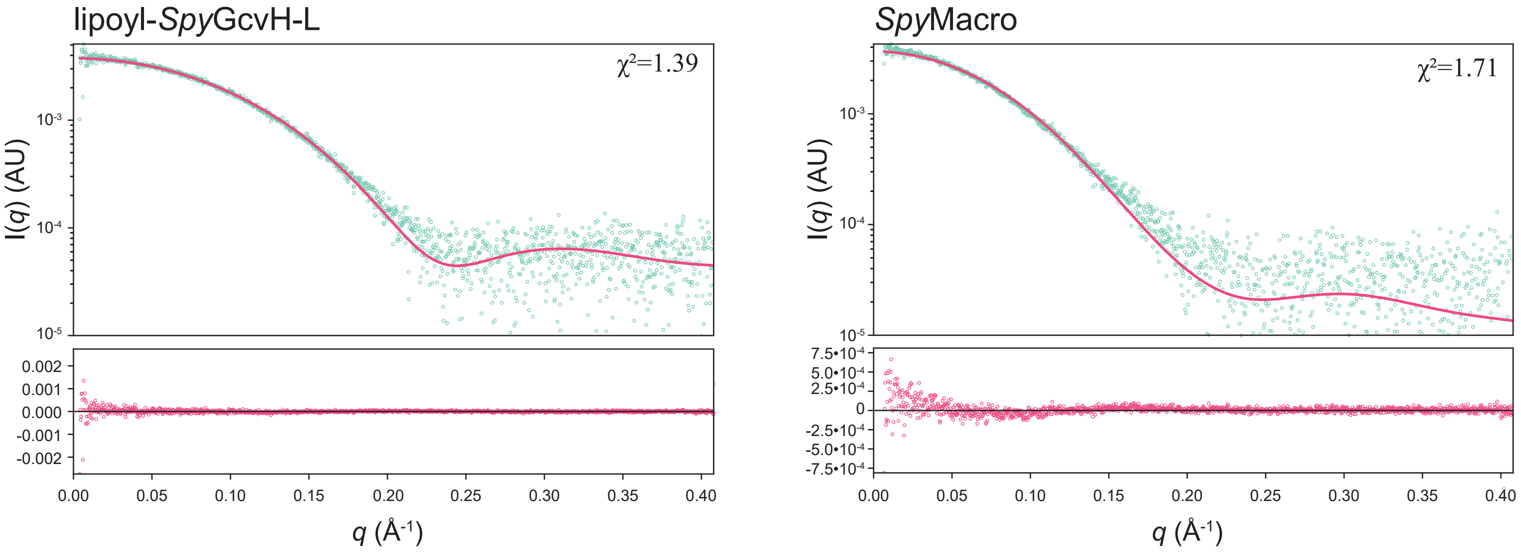

lipoyl-*Spy*GcvH-L
$\chi^2$=1.39
I(q) (AU)
q (Å⁻¹)
*Spy*Macro
$\chi^2$=1.71
I(q) (AU)
q (Å⁻¹)

**Figure S13. Non-physiological $Zn^{2+}$ ions involved in crystal packing of *Mor*Macro:Asn-ADPr.** Electron density omit maps (2Fo-Fc contoured at 1 σ) refined in the absence of any ligand. The final refined protein–ligand structures are shown as reference. (a) $Zn_2$ is octahedrally coordinated at the protomer-protomer interface with the amino-carboxylate ‘backbone’ of the Asn-ADPr from protomer A (black/green) occupying two (bidentate) and Glu195 from protomer B (white) one coordination site. The remaining three sites are occupied by water molecules. (b) $Zn_3$ is tetrahedrally coordinated and creates symmetry contacts using the same residues (Glu72/$Glu72_{sym}$ and H239/$H239_{sym}$).

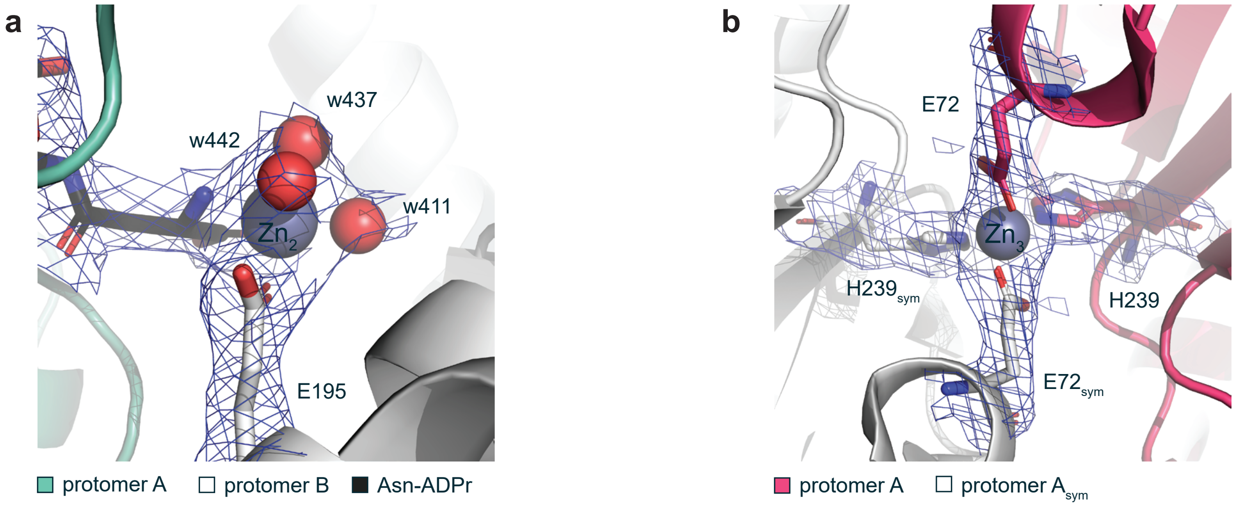
a
w437
w442
w411
Zn2
E195
protomer A
protomer B
Asn-ADPr
b
E72
Zn3
H239sym
H239
E72sym
protomer A
protomer Asym

**Figure S14. Structural model of *Foc1*Mfs1**. *Foc1*Mfs1 was modelled using AlphaFold 3 and include two $Zn^{2+}$ ion, one $NAD^{+}$, and one ADP ligand. Model quality indicators ipTM and pTM are given in Tab. S5. (a) Per-residue confidence estimate (pLDDT) mapped onto the predicted structure. The modelled ligands are given as: zinc (grey sphere) and $NAD^{+}$/ADP (black sticks), respectively. (b) Expected position error (EPE) of the predicted structure. (c) Structural alignment of the five model outputs (RMSD values 0.28 – 0.69).

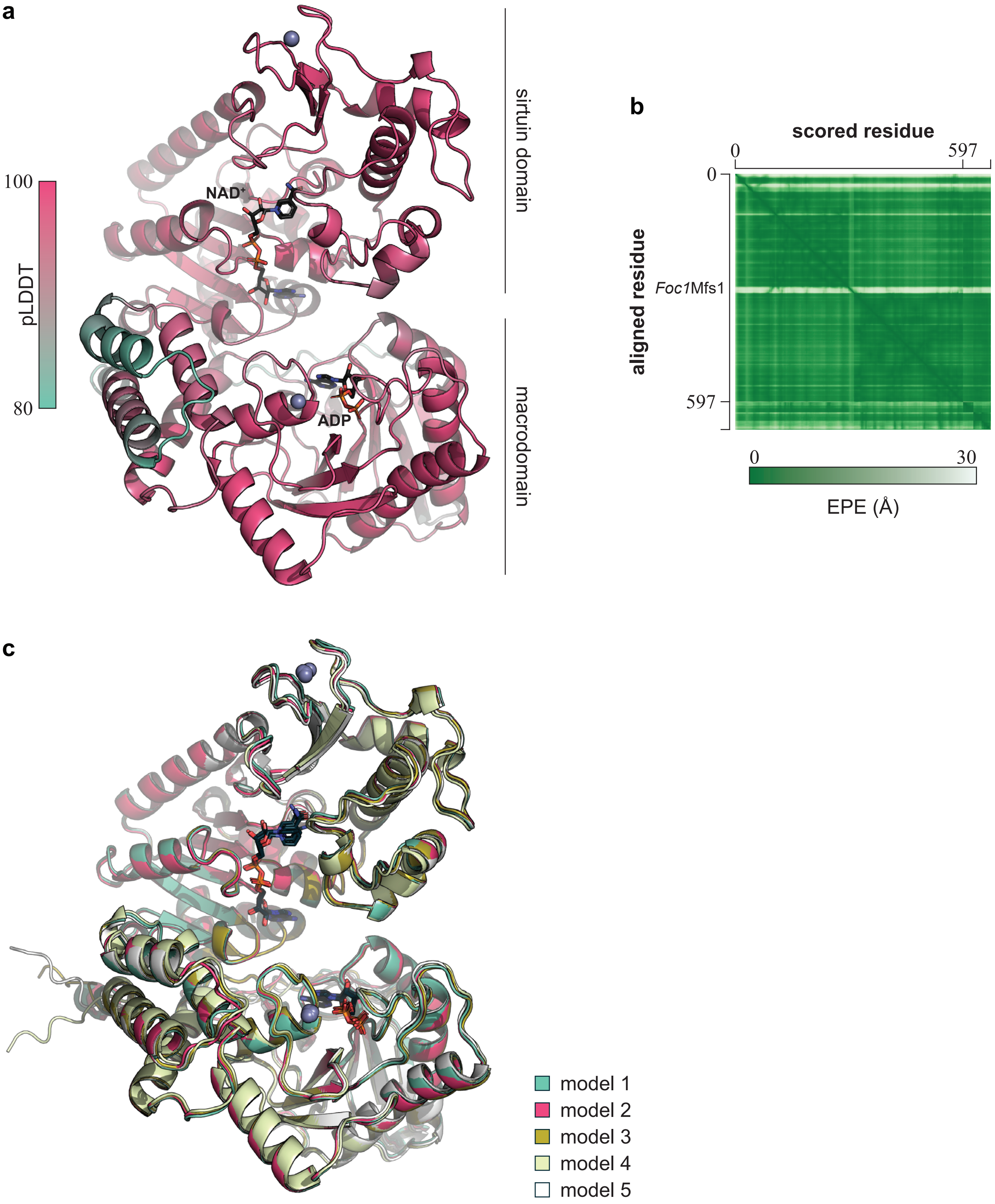

a
100
pLDDT
80
NAD+
ADP
sirtuin domain
macrodomain
b
scored residue
0
597
aligned residue
Foc1Mfs1
0
597
0
30
EPE (Å)
c
model 1
model 2
model 3
model 4
model 5

# Supplemental Tables

**Table S1.** Amino acid sequences used for phylogenetic analysis of the MacroD-type class.

| species | MacroD subclass | protein | abbreviation | accession number[i] |
|---|---|---|---|---|
| *Alligator mississippiensis* | GDAP2-like | GDAP2-like | | KYO28786 |
| *Homo sapiens* | GDAP2-like | GDAP2 | *h*GDAP2 | AAH13132 |
| *Malus domestica* | GDAP2-like | GDAP2-like | | XP_017182036 |
| *Physcomitrella patens* | GDAP2-like | GDAP2-like | | XP_001776374 |
| *Plutella xylostella* | GDAP2-like | GDAP2-like | | XP_011555499 |
| *Trichoplax adhaerens* | GDAP2-like | GDAP2-like | | XP_002112464 |
| *Amborella trichopoda* | MacroD1/2-like | MacroD1/2-like | | XP_011622649 |
| *Arabidopsis thaliana* | MacroD1/2-like | MacroD1/2-like | | NP_030605 |
| *Aspergillus fumigatus* | MacroD1/2-like | MacroD1/2-like | | XP_754239 |
| *Cryptococcus neoformans* var. *neoformans* | MacroD1/2-like | MacroD1/2-like | | XP_772446 |
| *Cryptomeria japonica* | MacroD1/2-like | MacroD1/2-like | | GLJ47930 |
| *Danio rerio* | MacroD1/2-like | MacroD1 | | NP_001004573 |
| *Danio rerio* | MacroD1/2-like | MacroD2 | | NP_956843 |
| *Fusarium oxysporum* f. sp. *cubense* race1 | MacroD1/2-like | MacroD1/2-like | | OR136504 |
| *Geotrypetes seraphini* | MacroD1/2-like | MacroD1 | | XP_033812488 |
| *Homo sapiens* | MacroD1/2-like | MacroD1 | *h*MacroD1 | NP_054786 |
| *Homo sapiens* | MacroD1/2-like | MacroD2 | *h*MacroD2 | NP_542407 |
| *Meleagris gallopavo* | MacroD1/2-like | MacroD2 | | XP_019468071 |
| *Petromyzon marinus* | MacroD1/2-like | MacroD1 | | XP_032820005 |
| *Phaseolus vulgaris* | MacroD1/2-like | MacroD1/2-like | | XP_007161504 |
| *Rhizopus microsporus* | MacroD1/2-like | MacroD1/2-like | | XP_023466972 |
| *Scedosporium apiospermum* | MacroD1/2-like | MacroD1/2-like | | XP_016645544 |
| *Sorghum bicolor* | MacroD1/2-like | MacroD1/2-like | | XP_002467900 |
| Getah Virus | nsp3 MOD1-like | nsp3 | | YP_164438 |
| Hepatitis E virus | nsp3 MOD1-like | pORF1 | | AHC70111 |
| Human coronavirus 229E | nsp3 MOD1-like | nsp3 | | NP_073549 |
| Human coronavirus NL63 | nsp3 MOD1-like | nsp3 | | YP_003766 |
| Human coronavirus OC43 | nsp3 MOD1-like | nsp3 | | AGT51736 |
| Middle East respiratory syndrome-related coronavirus | nsp3 MOD1-like | nsp3 | | YP_009047215 |
| Severe acute respiratory syndrome coronavirus 2 | nsp3 MOD1-like | nsp3 | | YP_009725299 |
| Venezuelan equine encephalitis virus | nsp3 MOD1-like | nsp3 | | NP_740698 |
| Western equine encephalitis virus | nsp3 MOD1-like | nsp3 | | NP_818935 |
| *Acropora digitifera* | PARP MOD1-like | | | XP_015769171 |
| *Archaeoglobus fulgidus* | PARP MOD1-like | AF1521 | | AAB89725 |
| *Homo sapiens* | PARP MOD1-like | PARP14 | *h*PARP14 MOD1 | XP_011511231 |
| *Homo sapiens* | PARP MOD1-like | PARP9 | *h*PARP9 MOD1 | NP_001139574 |
| *Meleagris gallopavo* | PARP MOD1-like | PARP14-like | | XP_010712168 |
| *Opisthocomus hoazin* | PARP MOD1-like | PARP9-like | | KFR02243 |
| *Pristis pectinata* | PARP MOD1-like | PARP14-like | | XP_051868994 |
| *Rhincodon typus* | PARP MOD1-like | PARP9-like | | XP_048455185 |
| *Tetraodon nigroviridis* | PARP MOD1-like | PARP9-like | | CAF98989 |
| *Thaumarchaeota archaeon* MY3 | PARP MOD1-like | | | ALI34863 |
| *Thermosipho africanus* | PARP MOD1-like | | | ACJ74881 |
| *[Clostridium] asparagiforme* | Zn-Macro | Zn-Macro | | EEG54164 |
| *Aspergillus terreus* | Zn-Macro | Mfs1 | *Ate*Mfs1 | OR133610 |

| species | MacroD subclass | protein | abbreviation | accession number[i] |
|---|---|---|---|---|
| *Candida albicans* | Zn-Macro | Mfs1 | *Cal*Mfs1 | EEQ47258 |
| *Fusarium oxysporum* f. sp. *cubense* race1 | Zn-Macro | Mfs1 | *Foc1*Mfs1 | OR133608 |
| *Jeotgalicoccus saudimassiliensis* | Zn-Macro | Zn-Macro | | WP_035811782 |
| *Methanobrevibacter gottschalkii* | Zn-Macro | Zn-Macro | | SEK23412 |
| *Methanobrevibacter olleyae* | Zn-Macro | Zn-Macro | | MBE6511662 |
| *Methanobrevibacter oralis* | Zn-Macro | Zn-Macro | *Mor*Macro | WP_042691995 |
| *Methanobrevibacter ruminantium* | Zn-Macro | Zn-Macro | | MCI5737402 |
| *Phoenicibacter congonensis* | Zn-Macro | Zn-Macro | | WP_079536779 |
| *Phytophthora nicotianae* var. *parasitica* | Zn-Macro | Mfs1 | *Pnp*Mfs1 | OR133609 |
| *Pseudoramibacter alactolyticus* | Zn-Macro | Zn-Macro | | EFV00712 |
| *Staphylococcus aureus* subsp. *aureus* | Zn-Macro | Zn-Macro | *Sau*Macro | BAB56487 |
| *Streptococcus pyogenes* | Zn-Macro | Zn-Macro | *Spy*Macro | AAK34075 |
| *Streptococcus urinalis* | Zn-Macro | Zn-Macro | | EHJ57565 |
| *Zongyangia hominis* | Zn-Macro | Zn-Macro | | WP_262398175 |
| *Acinetobacter baumannii* | | YmdB-like | | WP_150955863 |
| *Enterococcus faecium* | | YmdB-like | | SAZ11070 |
| *Escherichia coli* | | YmdB-like | | WP_000857399 |
| *Methanosarcina acetivorans* | | | | WP_048065167 |
| *Pseudomonas aeruginosa* | | YmdB-like | | WP_126858665 |

(i) GenBank accession numbers

**Table S2.** Amino acid sequences used for the alignment of MacroD-type class.

| species | protein | abbreviation | accession number[i] |
|---|---|---|---|
| *Homo sapiens* | GDAP2 | *h*GDAP2 | AAH13132 |
| *Meleagris gallopavo* | GDAP2 | *Mga*GDAP2 | XP_010717028 |
| *Xenopus laevis* | GDAP2 | *Xla*GDAP2 | NP_001088345 |
| *Takifugu rubripes* | GDAP2 | *Tru*GDAP2 | XP_003962291 |
| *Fusarium oxysporum* f. sp. *cubense* race1 | MacroD1/2-like | *Foc1*MacroD2-like | OR136504 |
| *Homo sapiens* | MacroD1 | *h*MacroD1 | NP_054786 |
| *Homo sapiens* | MacroD2 | *h*MacroD2 | NP_542407 |
| *Caenorhabditis elegans* | MacroD1/2-like | *Ce*MacroD | NP_502127 |
| *Arabidopsis thaliana*[ii] | MacroD1/2-like | *Ath*MacroD | NP_030605 |
| *Cryptococcus neoformans* var. *neoformans*[ii] | MacroD1/2-like | *Cne*MacroD | XP_772446 |
| Human coronavirus 229E | nsp3 | HCoV-229E | NP_073549 |
| Middle East respiratory syndrome-related coronavirus | nsp3 | HCoV-EMC | YP_009047215 |
| Severe acute respiratory syndrome coronavirus 2 | nsp3 | SARS-CoV-2 | YP_009725299 |
| Venezuelan equine encephalitis virus | nsp3 | VEEV | NP_740698 |
| Infectious bronchitis virus | nsp3 | IBV | ADA83575 |
| Onyong-nyong virus | nsp3 | ONNV | NP_740705 |
| Kyzylagach virus | nsp3 | KYZV | AHL27150 |
| Sleeping disease virus | nsp3 | SDV | NP_740655 |
| *Homo sapiens* | PARP14 | *h*PARP14 MOD1 | XP_011511231 |
| *Homo sapiens* | PARP9 | *h*PARP9 MOD1 | NP_001139574 |
| *Meleagris gallopavo* | PARP14-like | *Mga*PARP14 MOD1 | XP_010712168 |
| *Opisthocomus hoazin* | PARP9-like | *Oho*PARP9 MOD1 | KFR02243 |
| *Fusarium oxysporum* f. sp. *cubense* race1 | Mfs1 | *Foc1*Mfs1 | OR133608 |
| *Candida albicans*[ii] | Mfs1 | *Cal*Mfs1 | EEQ47258 |
| *Methanobrevibacter oralis* | Zn-Macro | *Mor*Macro | WP_042691995 |
| *Staphylococcus aureus* subsp. *aureus* | Zn-Macro | *Sau*Macro | BAB56487 |
| *Streptococcus pyogenes* | Zn-Macro | *Spy*Macro | AAK34075 |

(i) GenBank accession numbers
(ii) Fig. 2b only

**Table S3.** Amino acid sequences used for the phylogenetic analysis of Zn-Macros.

| species | genomic organisation | taxonomic group | abbreviation | accession number[i] |
|---|---|---|---|---|
| *Enterococcus faecalis* | extended operon | Bacteria | | EPH94914 |
| *Facklamia hominis* | extended operon | Bacteria | | WP 101974973 |
| *Jeotgalicoccus saudimassiliensis* | extended operon | Bacteria | | WP 035811782 |
| *Lentilactobacillus parakefiri* | extended operon | Bacteria | | WP 095339553 |
| *Lentilactobacillus sunkii* | extended operon | Bacteria | | WP 057826285 |
| *Nosocomiicoccus ampullae* | extended operon | Bacteria | | WP 068130211 |
| *Salinicoccus halitifaciens* | extended operon | Bacteria | | WP 230820419 |
| *Staphylococcus aureus subsp. aureus* | extended operon | Bacteria | *Sau*Macro | BAB56487 |
| *Staphylococcus xylosus* | extended operon | Bacteria | | WP 017723916 |
| *Streptococcus pyogenes* | extended operon | Bacteria | *Spy*Macro | AAK34075 |
| *Streptococcus urinalis* | extended operon | Bacteria | | EHJ57565 |
| *Entamoeba dispar* | fusion | Amoebae | *Edi*Mfs1 | XP 001741521 |
| *Entamoeba invadens* | fusion | Amoebae | | XP 004259040 |
| *Planoprotostelium fungivorum* | fusion | Amoebae | | PRP81056 |
| *Aspergillus nidulans* | fusion | Fungi | | XP 050468317 |
| *Aspergillus terreus* | fusion | Fungi | *Ate*Mfs1 | OR133610 |
| *Aureobasidium pullulans* | fusion | Fungi | | TIA58419 |
| *Candida albicans* | fusion | Fungi | *Cal*Mfs1 | XP 714874 |
| *Candida dubliniensis* | fusion | Fungi | | XP 002418633 |
| *Colletotrichum higginsianum* | fusion | Fungi | | XP 018161445 |
| *Cucurbitaria berberidis* | fusion | Fungi | | XP 040784936 |
| *Fusarium oxysporum* f. sp. *cubense* race1 | fusion | Fungi | *Foc1*Mfs1 | OR133608 |
| *Ophiocordyceps australis* | fusion | Fungi | | PHH65891 |
| *Penicillium brasilianum* | fusion | Fungi | | CEO59896 |
| *Penicillium manginii* | fusion | Fungi | | KAJ5744284 |
| *Xylariaceae* sp. FL1651 | fusion | Fungi | | KAI8631252 |
| *Phytophthora nicotianae* var. *parasitica* | fusion | Oomycete | *Pnp*Mfs1 | OR133609 |
| *Methanobrevibacter gottschalkii* | operon | Archaea | | SEK23412 |
| *Methanobrevibacter millerae* | operon | Archaea | | MBE6511057 |
| *Methanobrevibacter olleyae* | operon | Archaea | | MBE6511662 |
| *Methanobrevibacter oralis* | operon | Archaea | *Mor*Macro | WP 042691995 |
| *Methanobrevibacter ruminantium* | operon | Archaea | | MCI5737402 |
| *Methanobrevibacter* sp. AbM4 | operon | Archaea | | WP 016358930 |
| *Methanobrevibacter* sp. YE315 | operon | Archaea | | WP 067039744 |
| *Methanobrevibacter wolinii* | operon | Archaea | | WP 042706970 |
| *Methanosphaera* sp. Vir-13MRS | operon | Archaea | | WP 274871168 |
| *Acetobacterium dehalogenans* | operon | Bacteria | | WP 026394350 |
| *Clostridium asparagiforme* | operon | Bacteria | | EEG54164 |
| *Clostridium* sp. CAG:149 | operon | Bacteria | | CCY85072 |
| *Dorea formicigenerans* | operon | Bacteria | | EGX77560 |
| *Hydrogeniiclostridium mannosilyticum* | operon | Bacteria | | WP 112331216 |
| *Phoenicibacter congonensis* | operon | Bacteria | | WP 079536779 |
| *Pseudoramibacter alactolyticus* | operon | Bacteria | | EFV00712 |
| *Treponema denticola* | operon | Bacteria | | AAS11168 |
| *Youxingia wuxianensis* | operon | Bacteria | | WP 262394108 |
| *Zongyangia hominis* | operon | Bacteria | | WP 262398175 |

(i) GenBank accession numbers

**Table S4.** Amino acid sequences used for the alignment of GcvH(-L) proteins.

| species | protein | abbreviation | accession number[i] |
|---|---|---|---|
| *Staphylococcus aureus* | GcvH-L | *Sau*GcvH-L | WP_000731878 |
| *Streptococcus pyogenes* | GcvH-L | *Spy*GcvH-L | WP_002984553 |
| *Dolosigranulum pigrum* | GcvH-L | *Dpi*GcvH-L | EHR34890 |
| *Lentilactobacillus hilgardii* | GcvH-L | *Lhi*GcvH-L | EEI70741 |
| *Aerococcus urinae* | GcvH | *Aur*GcvH | WP_284784177 |
| *Dolosigranulum pigrum* | GcvH | *Dpi*GcvH | WP_004636789 |
| *Granulicatella elegans* | GcvH | *Gel*GcvH | WP_227931594 |
| *Staphylococcus aureus* | GcvH | *Sau*GcvH | WP_000290491 |
| *Mycobacterium tuberculosis* | GcvH | *Mtu*GcvH | NP_216342 |
| *Pisum sativum* | GcvH | *Psa*GcvH | AAA33668 |
| *Bos taurus* | GcvH | *Bta*GcvH | NP_777269 |

(i) GenBank accession numbers

**Table S5.** Quality parameters for AlphaFold 3 models.

| model | Ligands | pTM | ipTM |
|---|---|---|---|
| *Foc1*Mfs1 | 2 $Zn^{2+}$, $NAD^{+}$, ADP | 0.9 | 0.92 |
| *Sau*Macro:*Sau*GcvH-L | 1 $Zn^{2+}$, ADP | 0.93 | 0.92 |
| *Spy*Macro:*Spy*GcvH-L | 1 $Zn^{2+}$, ADP | 0.93 | 0.91 |
| *Dpi*Macro:*Dpi*GcvH-L | 1 $Zn^{2+}$, ADP | 0.91 | 0.91 |
| *Sau*Macro:*Sau*GcvH | 1 $Zn^{2+}$, ADP | 0.78 | 0.72 |
| *Dpi*Macro:*Dpi*GcvH | 1 $Zn^{2+}$, ADP | 0.75 | 0.71 |
| *Efa*Macro:*Spy*GcvH-L | 1 $Zn^{2+}$, ADP | 0.7 | 0.76 |
| *Tpe*Macro:*Spy*GcvH-L | 1 $Zn^{2+}$, ADP | 0.72 | 0.66 |

**Table S6.** SAXS sample, data-collection, analysis, and 3D modelling details for *Spy*Macro, lipoyl-*Spy*GcvH-L, and their complex

| | | | |
|---|---|---|---|
| **(a) Sample details** | | | |
| Organism | *S. pyogenes* | | |
| Source | *E. coli* (Rosetta (DE3) recombinant expression) | | |
| Description of complex | *Spy*Macro:lipoyl-*Spy*GcvH-L | | |
| **Scattering particle composition** | | | |
| | Component 1 | Component 2 | Complex |
| Protein(s) | lipoyl-*Spy*GcvH-L | *Spy*Macro | AlphaFold model[i] |
| **Sample environment/configuration** | | | |
| Solvent composition | 25 mM TrisHCl (pH 8), 150 mM NaCl, 2.5 mM TCEP, 3% (v/v) glycerol | | |
| Sample temperature (K) | 288 | | |
| In-beam sample cell | 1.5 mm quartz capillary, sample flow at 1 µL/sec | | |
| **Size exclusion chromatography (SEC-SAS)** | | | |
| | Component 1 | Component 2 | Complex |
| Sample injection concentration (µM) | 577 | 210 | 186/155[ii] |
| Sample injection volume (µL) | 45 | 45 | 45 |
| SEC column type | KW402.5-4F (Shodex) | | |
| SEC flowrate (mL/min) | 0.075 | | |
| **(b) SAS data collection** | | | |
| | SAXS | | |
| Data acquisition/reduction software | General Data Acquisition (GDA), DAWN Science (Diamond Light Source, UK) | | |
| SAXS beamline | B21 High-throughput beamline (Diamond Light Source, UK) | | |
| Measured *q*-range ($q_{min} - q_{max}$; Å$^{-1}$) | 0.04 - 0.40 | | |
| Method for scaling intensities | Scaled to absolute units (cm$^{-1}$) relative to water | | |
| Exposure time (sec) / № of exposures [SEC-SAS frames used for averaging] | 3 / 600 [15] | | |
| **(c) SAS-derived structural parameters** | | | |
| Methods/software | Scatter (Bioisis) | | |
| **Guinier analysis** | | | |
| | Component 1 | Component 2 | Complex |
| $I(0) \pm \sigma$ (cm$^{-1}$) | $0.0038 \pm 1.1 \cdot 10^{-5}$ | $0.0038 \pm 1.5 \cdot 10^{-5}$ | $0.0075 \pm 2.0 \cdot 10^{-5}$ |
| $R_g \pm \sigma$ (Å) | 14.94 ± 1.13 | 20.61 ± 1.55 | 24.38 ± 1.76 |
| $min < qR_g < max$ limit | 0.010 – 0.087 | 0.012 – 0.062 | 0.087 – 0.053 |
| Linear fit assessment | 0.96 | 0.83 | 0.88 |
| **P(r) analysis** | | | |
| | Component 1 | Component 2 | Complex |
| $I(0) \pm \sigma$ (cm$^{-1}$) | $0.038 \pm 0.01 \cdot 10^{-4}$ | $0.0038 \pm 0.14 \cdot 10^{-4}$ | $0.0074 \pm 0.23 \cdot 10^{-4}$ |
| $R_g \pm \sigma$ (Å) | 14.93 ± 0.058 | 20.62 ± 0.11 | 24.6 ± 0.11 |
| $d_{max}$ (Å) | 50.0 | 73.0 | 84.0 |
| *q-range* (Å$^{-1}$) | 0.01 – 0.306 | 0.0123 – 0.2511 | 0.0087 – 0.2511 |
| *P(r) fit assessment*[iii] | 0.67 | 0.74 | 0.72 |
| **(d) Scattering particle size** | | | |
| Methods/software | Mulch (University of Sydney), Protparam (Expasy), Scatter (Bioisis) | | |
| **Molecular weight (MW) estimates** | | | |
| | Component 1 | Component 2 | Complex |
| MW from sequence (kDa) | 13.8[iv] | 30.3 | 43.8 |
| Volume from sequence (Å$^3$) | 16,506 | 37,060 | 53,506 |
| Volume from P(r) (Å$^3$) | 14,100 | 32,307 | 49,663 |
| **(e) Modelling** | | | |
| Method/software | MONSA | | |
| **Shape modelling** | | | |
| | Component 1 | Component 2 | Complex |
| Symmetry assumption | P1 | P1 | P1 |

| | | | |
|---|---|---|---|
| MONSA multiple phase model: $R_g$ (Å) / volume (Å$^3$) | 15 / 21,400 | 20.1 / 41,900 | 24.3 / 63,300 |
| № of individual model reconstructions | 10 | 10 | 10 |
| MONSA fit parameters ($\chi^2$) | 1.28 | 1.17 | 1.01 |
| *q*-range for fit (Å$^{-1}$) | 0.01 – 0.25 | 0.01 – 0.25 | 0.01 – 0.25 |
| Method/Software | X-ray structure/AlphaFold model compared to SAXS using FOXS (Sali Lab) | | |
| *q*-range for fit (Å$^{-1}$) | 0.008 – 0.36 | 0.008 – 0.36 | 0.008 – 0.36 |
| FOXS fit parameters ($\chi^2$) | 1.39 | 1.71 | 1.16 |
| **(f) Data and model deposition** | | | |
| | Component 1 | Component 2 | Complex |
| SASBDB IDs | SASDTX8 | SASDTY8 | SASDTZ8 |

(i) The complex was model with AlphFold-Multimer (v2.3.2; implemented on Colab)
(ii) lipoyl-*Spy*GcvH-L was used in 1.2-fold molar excess
(iii) CorMap p-value
(iv) Protparam value (13.6 kDa) plus lipoy modification (0.2 kDa)

**Table S7.** Summary of crystallisation procedures.

| PDB code | Method/component | Protein buffer | Final ML | Seed crystal ML |
|---|---|---|---|---|
| 8RSL | Crystallisation<br>*Sau*Macro: 450 µM | 15 mM TrisHCl [pH8]<br>125 mM NaCl<br>1.5 mM DTT | 18.5% (v/v) PEG400<br>12.5% (v/v) 1-propanol<br>4% (v/v) glycerol | |
| 8RSM | Co-crystallisation<br>*Spy*Macro: 200 µM<br>ADPr: 1 mM | 10 mM TrisHCl [pH 8]<br>100 mM NaCl<br>1 mM aspartic acid<br>1 mM DTT | 100 mM sodium cacodylate [pH 6.5]<br>27% (w/v) PEG2000MME | 0.12 M monosaccharides (20 mM D-Glucose, 20 mM D-Mannose, 20 mM D-Galactose, 20 mM L-Fucose, 20 mM D-Xylose, 20 mM *N*-Acetyl-D-Glucosamine)<br>100 mM sodium HEPES-MOPS buffer [pH 7.5]<br>20% (v/v) ethylene glycol<br>10% (w/v) PEG8000 |
| 8RSI | Crystallisation<br>*Mor*Macro: 1.2 mM | 10 mM TrisHCl [pH 8]<br>100 mM NaCl<br>1 mM DTT | 46.8 mM MES monohydrate<br>53.2 mM imidazole<br>20% (w/v) ethylene glycol<br>10% (w/v) PEG8000 | |
| 8RSJ | Co-Crystallisation<br>*Mor*Macro: 1.2 mM<br>ADPr: 6 mM | 10 mM TrisHCl [pH 8]<br>100 mM NaCl<br>1 mM DTT | 200 mM sodium fluoride<br>1 M sodium tartrate tetrahydrate<br>100 mM BisTris propane [pH 7.5]<br>20% (w/v) PEG3350<br>3% (v/v) glycerol | |
| 8RSK | Co-crystallisation<br>*Mor*Macro: 1.2 mM<br>Asn-ADPr: 6 mM[i] | 10 mM TrisHCl [pH 8]<br>100 mM NaCl<br>1 mM DTT | 5 mM zinc acetate<br>9% (w/v) PEG8000<br>100 mM sodium cacodylate [pH 6.5] | |
| 8RSN | Crystallisation<br>*Foc1*Mfs1: 125 µM | 10 mM PIPES [pH 7]<br>75 mM NaCl<br>1 mM TCEP | 200 mM potassium thiocyanate<br>100 mM BisTris propane [pH 7.5]<br>20% (w/v) PEG3350 | [ii]200 mM $MgCl_2$<br>100 mM HEPES [pH 7]<br>20% (w/v) PEG6000 |

(i) Lyophilised Asn-ADPr was directly dissolved into protein solution
(ii) A single protein crystal grew over the course of six months

# NMR data of all compounds

## $^{1}$H-NMR of Compound **4**

1609Qiang.36.fid — liu-26-1 — biosyn1Hfast CDCl3 /opt/DATA nmrafd 11

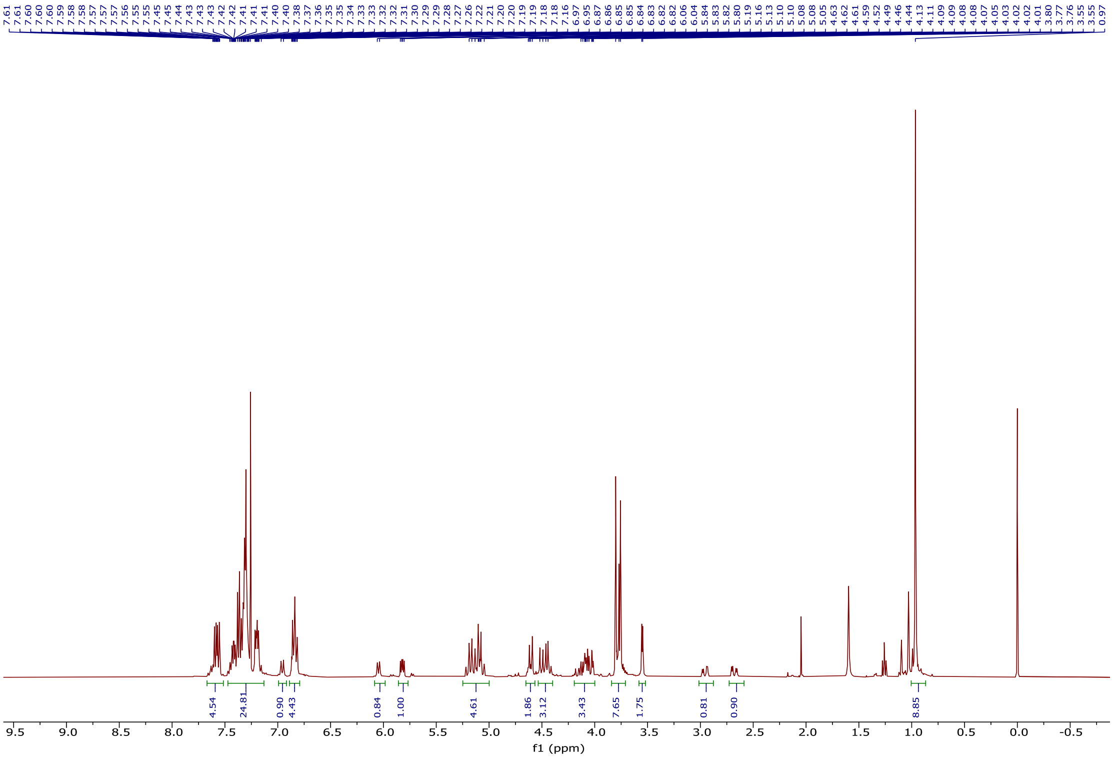

## $^{13}$C-NMR of Compound **4**

1609Qiang.37.fid — liu-26-1 — biosynAPTfast CDCl3 /opt/DATA nmrafd 11

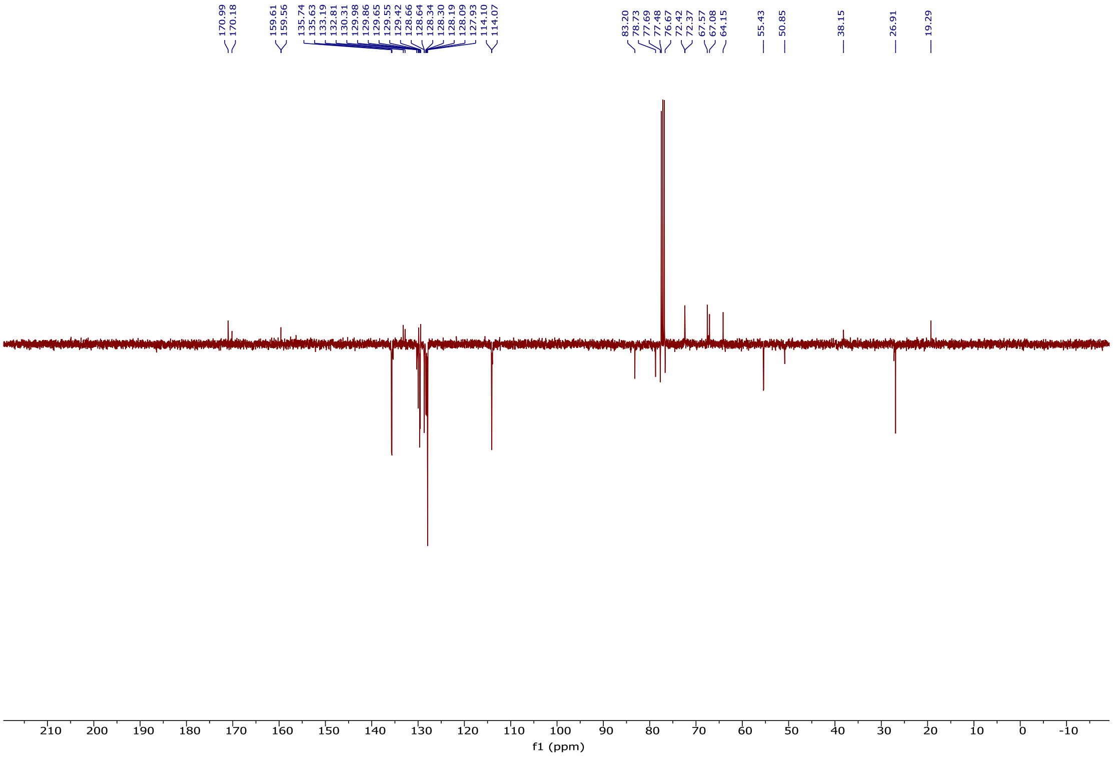

## $^{1}$H-NMR of Compound **5**

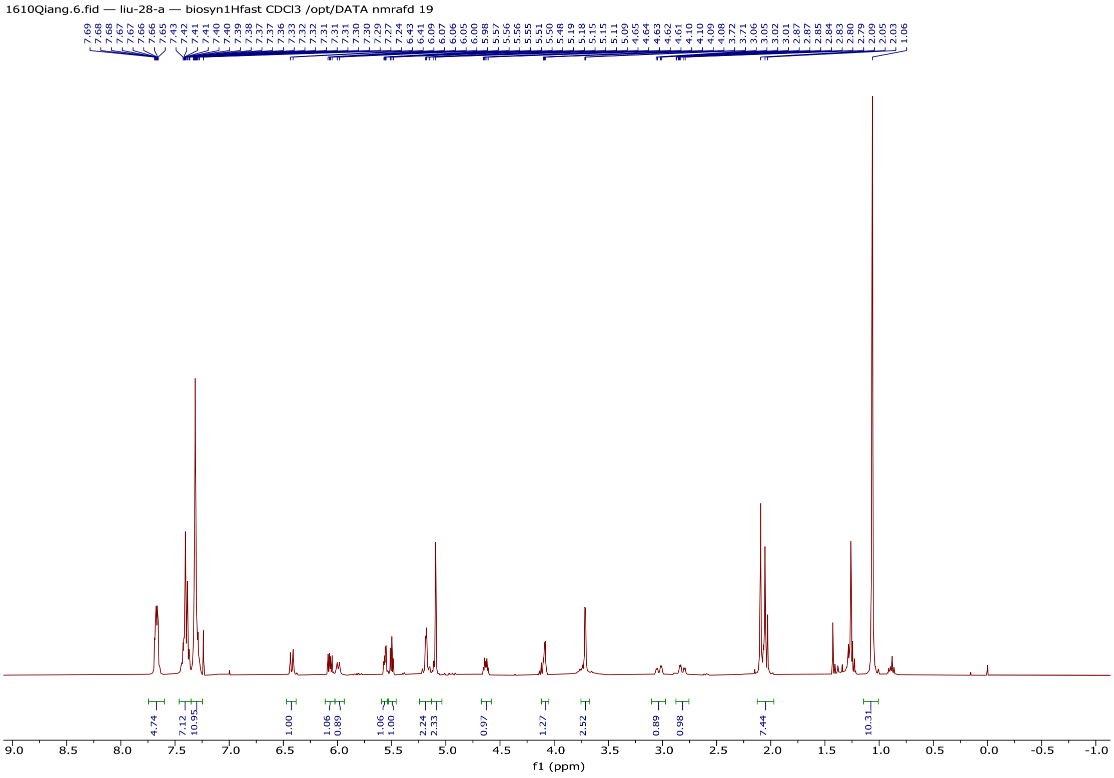

## $^{13}$C-NMR of Compound **5**

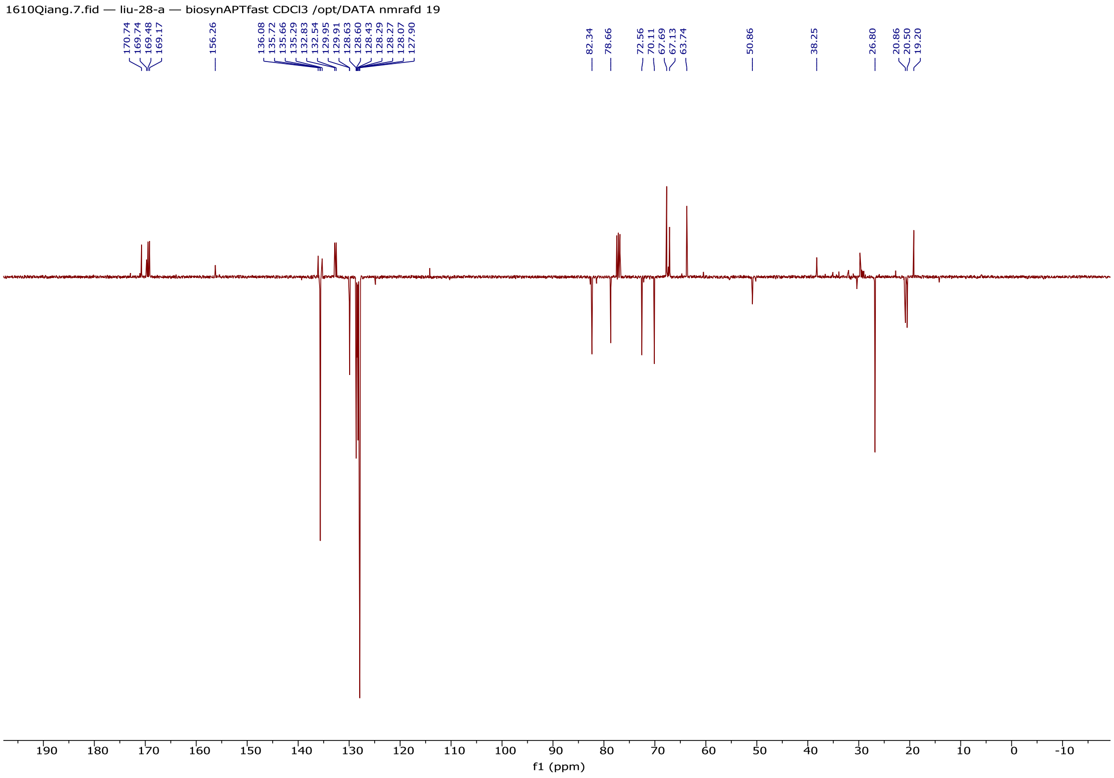

## $^{1}$H-NMR of Compound **6**

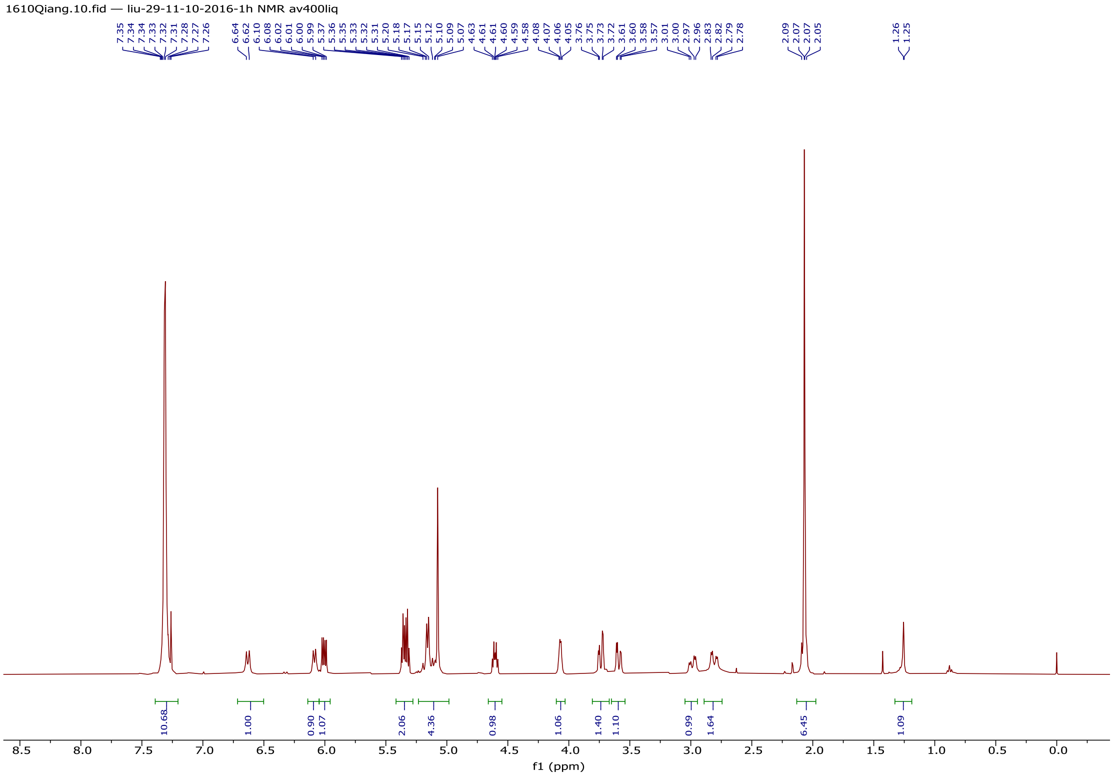


## $^{13}$C-NMR of Compound **6**

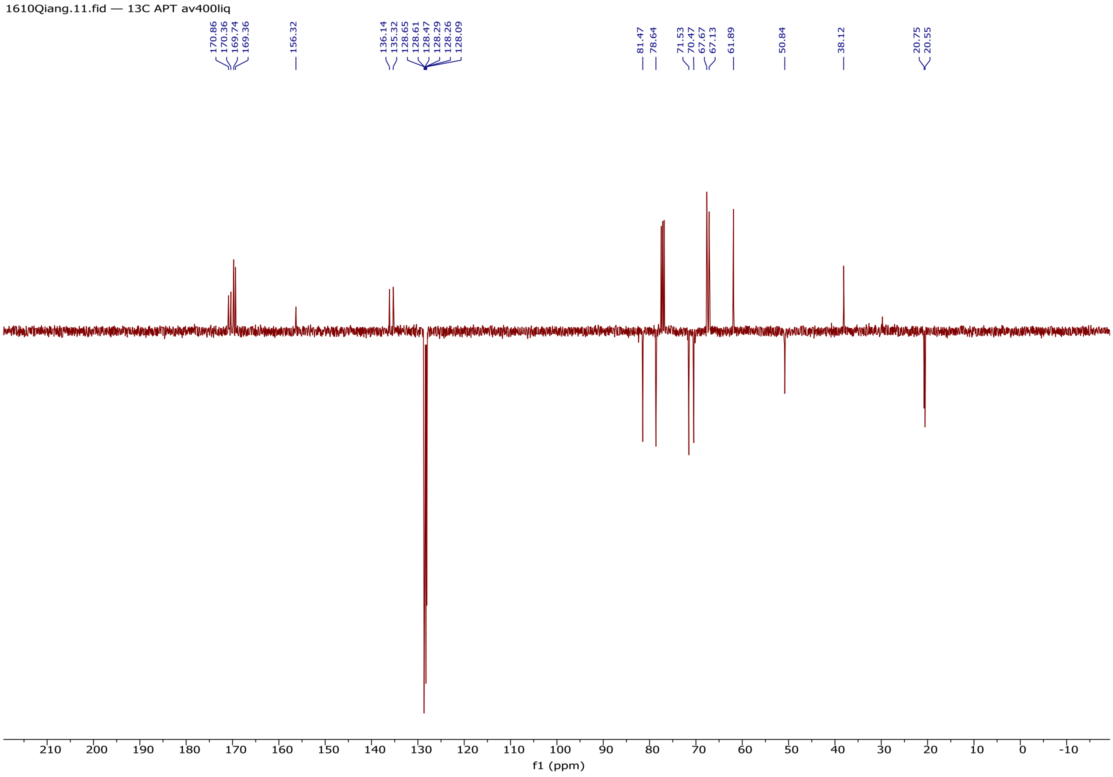

## $^{1}$H-NMR of Compound **7**

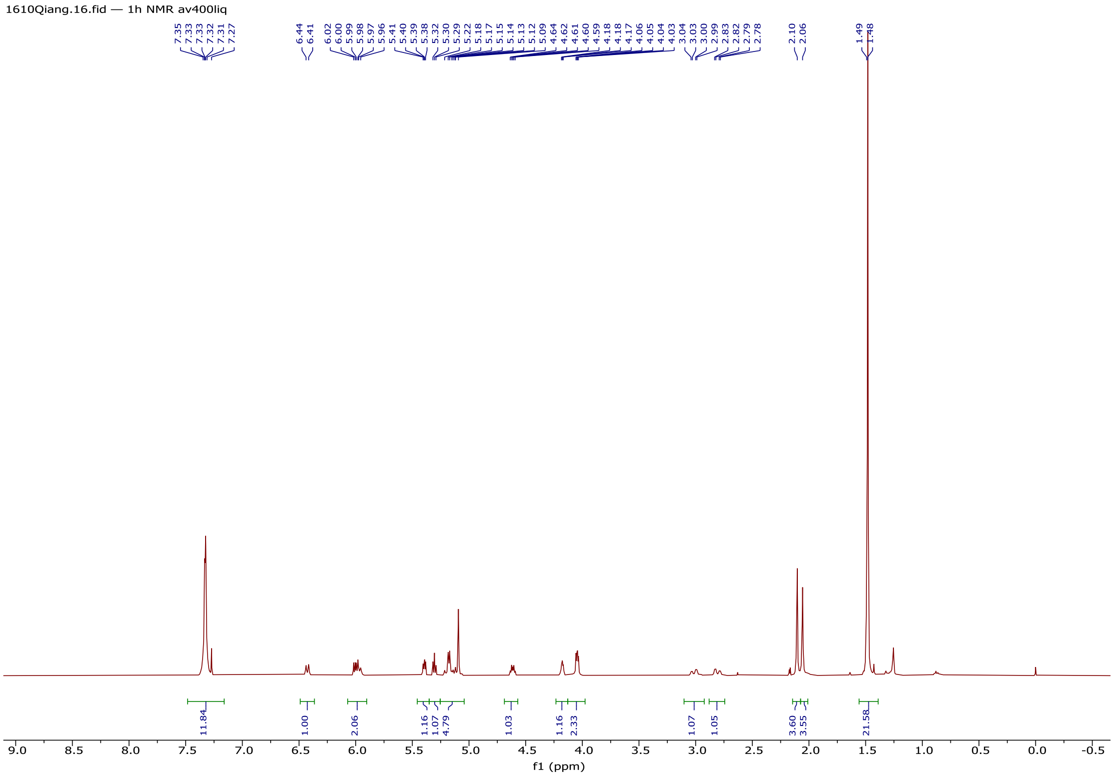


## $^{13}$C-NMR of Compound **7**

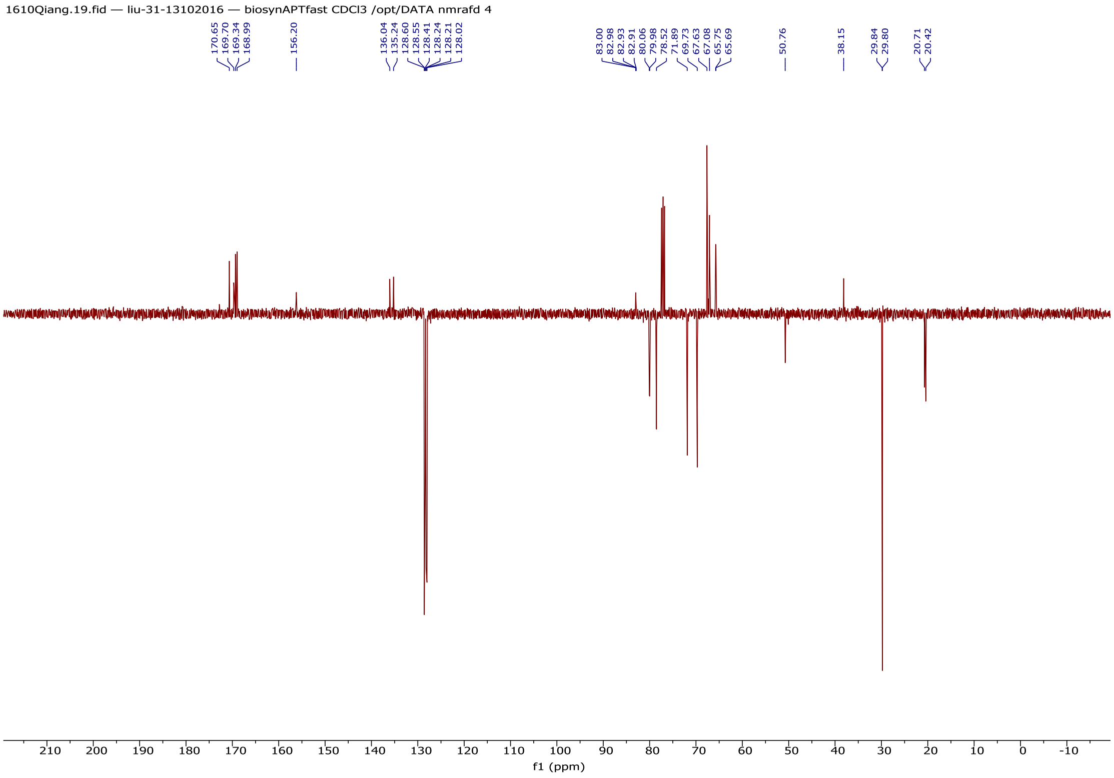

## $^{31}$P-NMR of Compound **7**

1610Qiang.17.fid — liu-31-13102016-31P-nmr-1H-dec. sw=200_-50ppm

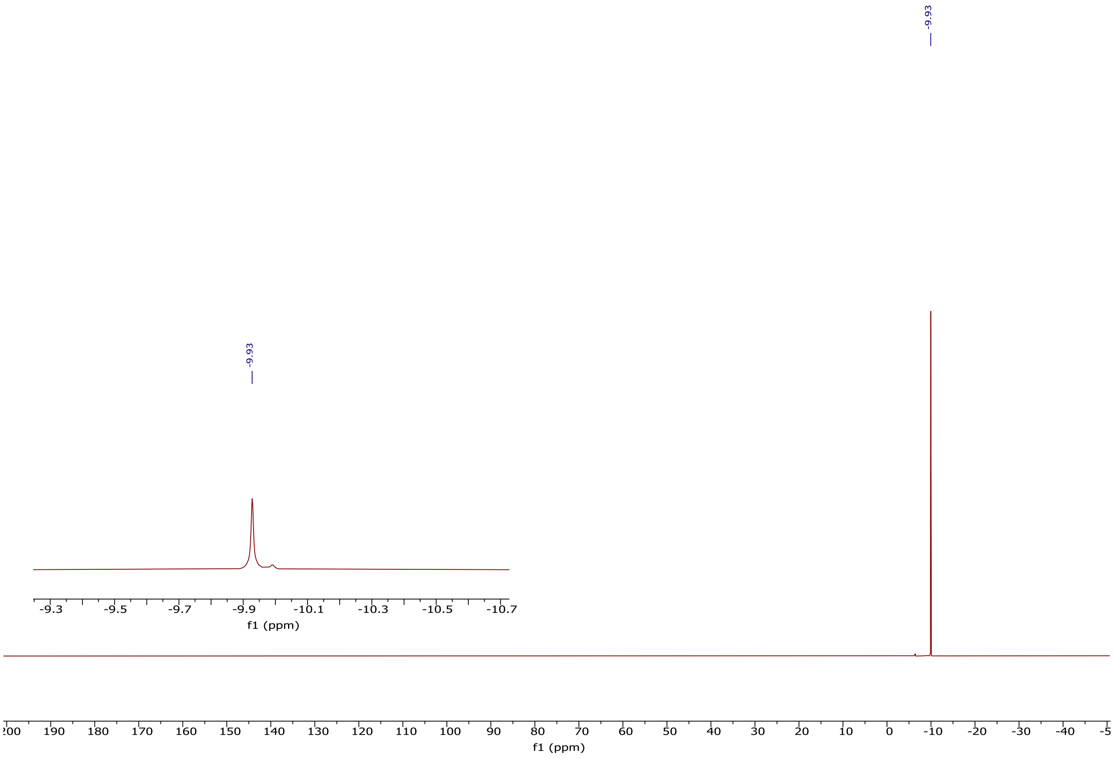


## $^{31}$P-NMR of Compound **9**

1611Qiang.17.fid — liu-39-25112016-31P-nmr-1H-dec. sw=200_-50ppm

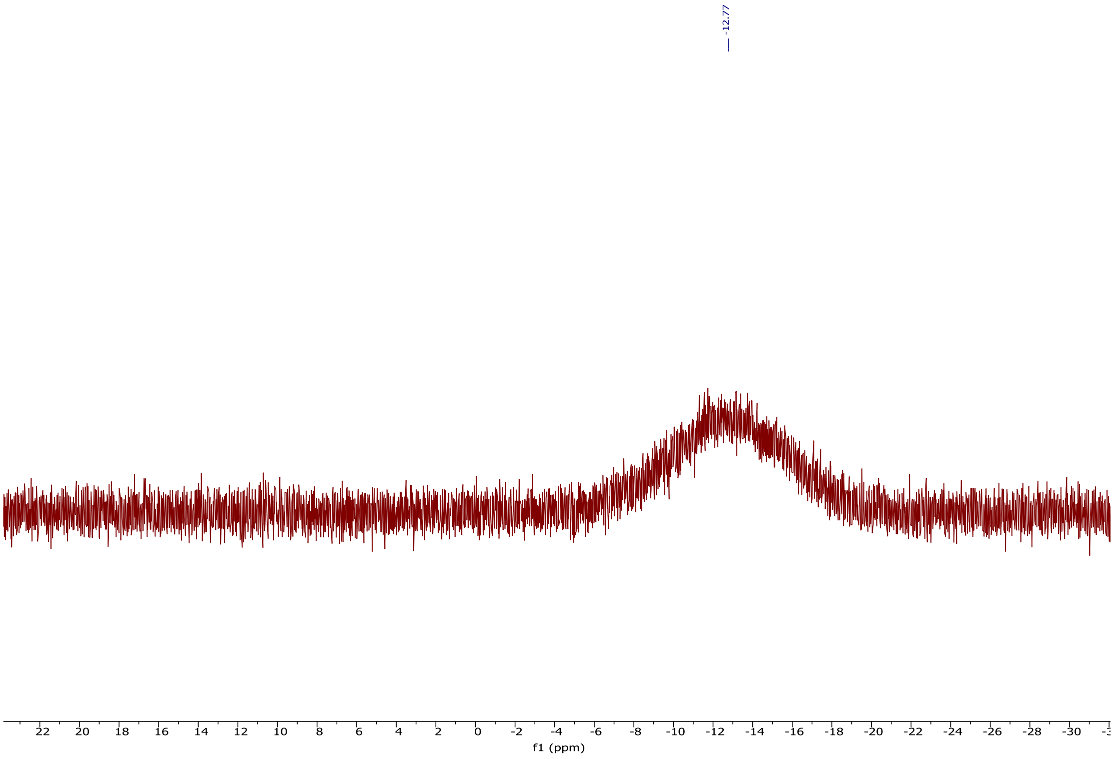

## $^{1}$H-NMR of Compound **1**

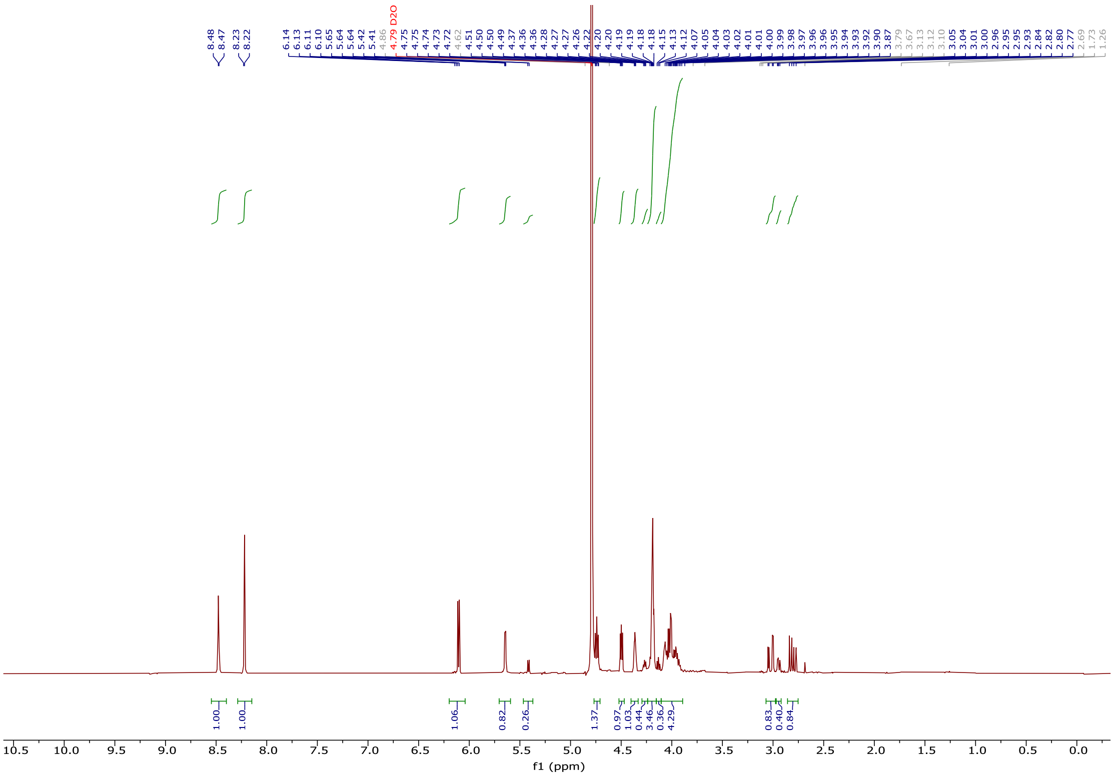

## $^{13}$C-NMR of Compound **1**

1612Qiang.3.fid — liu-41-04122016 — C13APT D2O /opt/DATA nmrafd 60

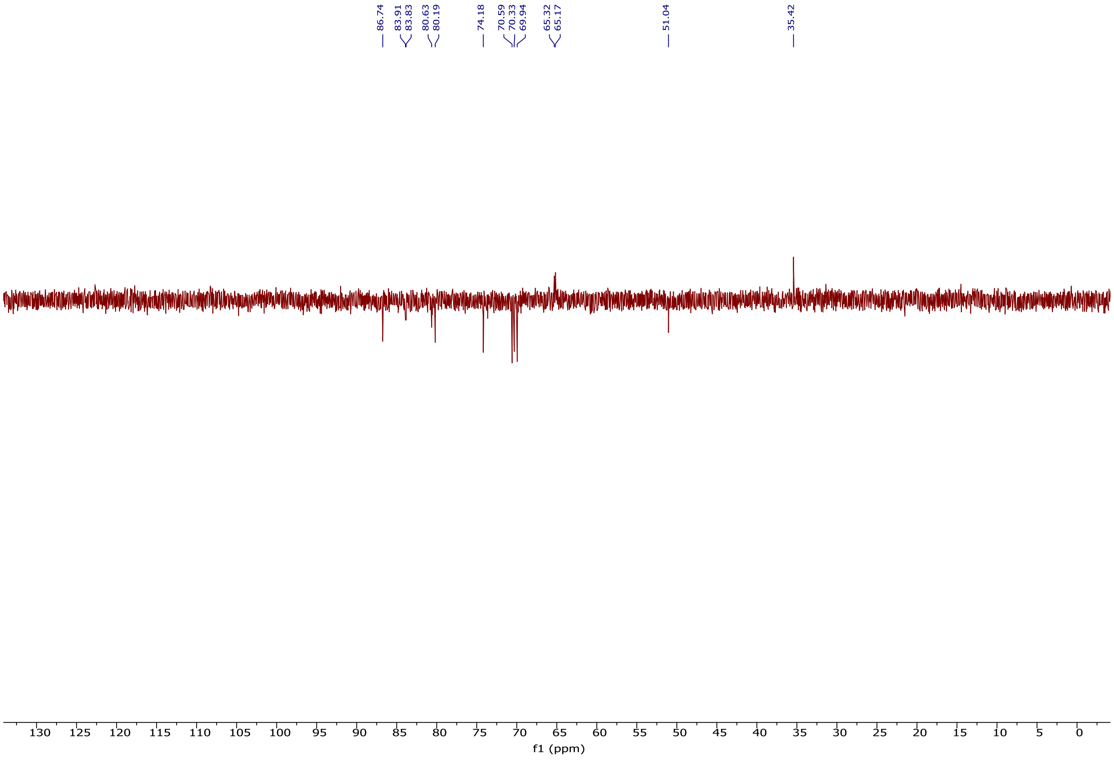

## $^{31}$P-NMR of Compound **1**

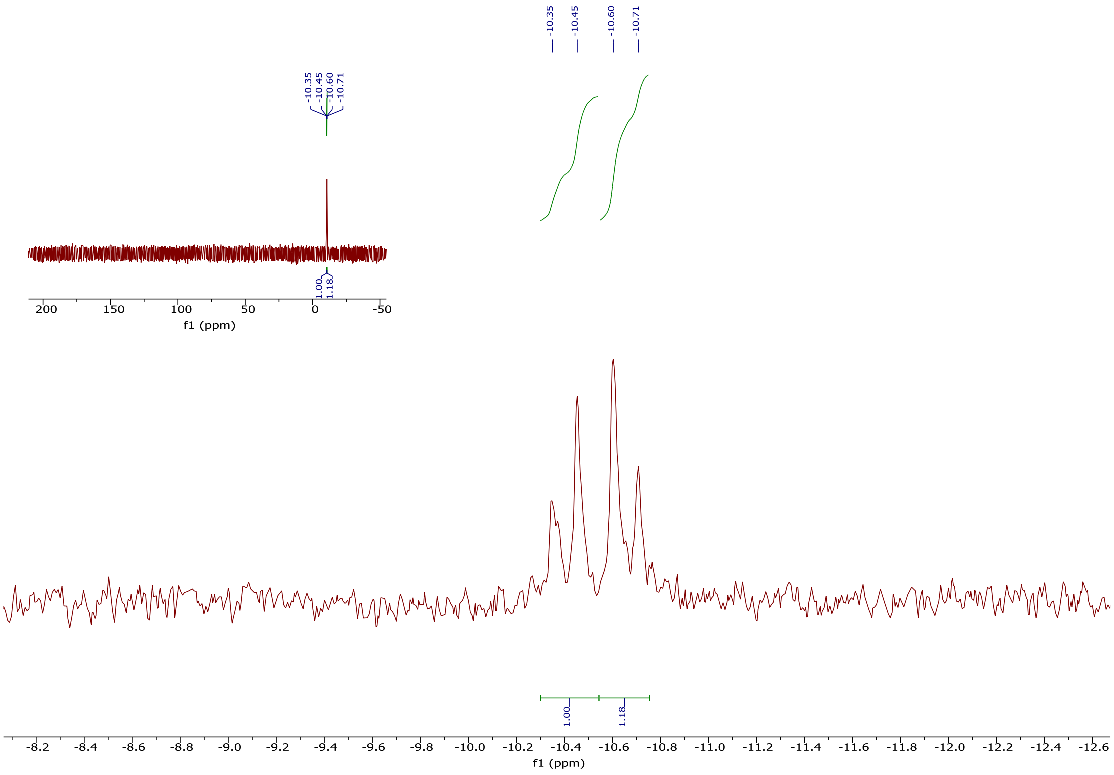

Supplement: Supporting Information [file mmc1.pdf]
